# Structural Variants Create New Topological-Associated Domains and Ectopic Retinal Enhancer-Gene Contact in Dominant Retinitis Pigmentosa

**DOI:** 10.1016/j.ajhg.2020.09.002

**Published:** 2020-10-05

**Authors:** Suzanne E. de Bruijn, Alessia Fiorentino, Daniele Ottaviani, Stephanie Fanucchi, Uirá S. Melo, Julio C. Corral-Serrano, Timo Mulders, Michalis Georgiou, Carlo Rivolta, Nikolas Pontikos, Gavin Arno, Lisa Roberts, Jacquie Greenberg, Silvia Albert, Christian Gilissen, Marco Aben, George Rebello, Simon Mead, F. Lucy Raymond, Jordi Corominas, Claire E.L. Smith, Hannie Kremer, Susan Downes, Graeme C. Black, Andrew R. Webster, Chris F. Inglehearn, L. Ingeborgh van den Born, Robert K. Koenekoop, Michel Michaelides, Raj S. Ramesar, Carel B. Hoyng, Stefan Mundlos, Musa M. Mhlanga, Frans P.M. Cremers, Michael E. Cheetham, Susanne Roosing, Alison J. Hardcastle

**Affiliations:** 1 Department of Human Genetics, Radboud University Medical Center, Nijmegen, 6500 HB, the Netherlands; 2 Donders Institute for Brain Cognition and Behaviour, Radboud University Medical Center, Nijmegen, 6500 HB, the Netherlands; 3 UCL Institute of Ophthalmology, London, EC1V 9EL, UK; 4 UK Inherited Retinal Disease Consortium; 5 Genomics England Clinical Interpretation Partnership; 6 Gene Expression and Biophysics Group, Division of Chemical, Systems and Synthetic Biology, Department of Integrative Biomedical Science, Institute for Infectious Disease & Molecular Medicine, Faculty of Health Sciences, University of Cape Town, Cape Town, 7935, South Africa; 7 Max Planck Institute for Molecular Genetics, RG Development & Disease, Berlin, 14195, Germany; 8 Institute for Medical and Human Genetics, Charité – Universitätsmedizin, Berlin, 10117, Germany; 9 Department of Ophthalmology, Radboud University Medical Center, Nijmegen, 6500 HB, the Netherlands; 10 Moorfields Eye Hospital, London, EC1V 2PD, UK; 11 Department of Genetics and Genome Biology, University of Leicester, Leicester, LE1 7RH, UK; 12 Clinical Research Center, Institute of Molecular and Clinical Ophthalmology Basel (IOB), Basel, 4031, Switzerland; 13 Department of Ophthalmology, University Hospital Basel, Basel, 4001, Switzerland; 14 University of Cape Town/MRC Genomic and Precision Medicine Research Unit, Division of Human Genetics, Department of Pathology, Institute of Infectious Disease and Molecular Medicine, Faculty of Health Sciences, University of Cape Town, Cape Town, 7935, South Africa; 15 MRC Prion Unit at UCL, UCL Institute of Prion Disease, London, W1W 7FF, UK; 16 NIHR BioResource, Cambridge University Hospitals, Cambridge, CB2 0QQ, UK; 17 Department of Medical Genetics, Cambridge Institute for Medical Research, University of Cambridge, Cambridge, CB2 OXY, UK; 18 Division of Molecular Medicine, Leeds Institute of Medical Research, University of Leeds, Leeds, LS2 9JT, UK; 19 Department of Otorhinolaryngology, Radboud University Medical Center, Nijmegen, 6500 HB, the Netherlands; 20 Oxford Eye Hospital, Oxford University Hospitals NHS Trust and Nuffield Laboratory of Ophthalmology, University of Oxford, Oxford, OX3 9DU, UK; 21 Manchester Centre for Genomic Medicine, St. Mary’s Hospital, Manchester, M13 9WL, UK; 22 The Rotterdam Eye Hospital, Rotterdam, 3011 BH, the Netherlands; 23 Department of Paediatric Surgery, Human Genetics and Ophthalmology, McGill University, Montréal, QC H4A 3J1, Canada; 24 Gene Expression and Biophysics Unit, Instituto de Medicina Molecular, Faculdade de Medicina Universidade de Lisboa, Lisbon, 1649-028, Portugal; 25 Epigenomics & Single Cell Biophysics Group, Radboud Institute for Molecular Life Sciences (RIMLS), Radboud University, Nijmegen, 6525 GA, the Netherlands

**Keywords:** whole-genome sequencing, dominant retinitis pigmentosa, RP17, structural variants, stem cells, retinal organoids, photoreceptor precursors cells, Hi-C, topologically associated domains, ectopic expression, GDPD

## Abstract

The cause of autosomal-dominant retinitis pigmentosa (adRP), which leads to loss of vision and blindness, was investigated in families lacking a molecular diagnosis. A refined locus for adRP on Chr17q22 (RP17) was delineated through genotyping and genome sequencing, leading to the identification of structural variants (SVs) that segregate with disease. Eight different complex SVs were characterized in 22 adRP-affected families with >300 affected individuals. All RP17 SVs had breakpoints within a genomic region spanning *YPEL2* to *LINC01476.* To investigate the mechanism of disease, we reprogrammed fibroblasts from affected individuals and controls into induced pluripotent stem cells (iPSCs) and differentiated them into photoreceptor precursor cells (PPCs) or retinal organoids (ROs). Hi-C was performed on ROs, and differential expression of regional genes and a retinal enhancer RNA at this locus was assessed by qPCR. The epigenetic landscape of the region, and Hi-C RO data, showed that *YPEL2* sits within its own topologically associating domain (TAD), rich in enhancers with binding sites for retinal transcription factors. The Hi-C map of RP17 ROs revealed creation of a neo-TAD with ectopic contacts between *GDPD1* and retinal enhancers, and modeling of all RP17 SVs was consistent with neo-TADs leading to ectopic retinal-specific enhancer-*GDPD1* accessibility. qPCR confirmed increased expression of *GDPD1* and increased expression of the retinal enhancer that enters the neo-TAD. Altered TAD structure resulting in increased retinal expression of *GDPD1* is the likely convergent mechanism of disease, consistent with a dominant gain of function. Our study highlights the importance of SVs as a genomic mechanism in unsolved Mendelian diseases.

## Introduction

Despite recent advances in next-generation sequencing, approximately 30%–40% of individuals with inherited retinal diseases (IRDs) lack a molecular diagnosis. This is probably due to a combination of rare novel disease genes, which require large cohorts for validation, and previously intractable mutation classes, such as intronic variants, structural variants (SVs), and variants in regulatory regions.^1^^,^^2^

The most common form of IRD is retinitis pigmentosa (RP [MIM: 268000]), which is genetically heterogeneous, with a prevalence of 1 in 4,000.^3^ RP is defined as a retinal degeneration that primarily affects rod photoreceptors, resulting in night blindness and progressive loss of peripheral vision, often progressing into the central retina and affecting cone photoreceptors, leading to severe visual impairment or blindness (see “Nonsyndromic Retinitis Pigmentosa Overview” in Web Resources). Autosomal-dominant RP (adRP) accounts for 25%–40% of cases, depending on the population studied, and has been associated with mutations in 30 genes, including *CA4* (MIM: 114760) on Chr17q23.1 (RP17 [MIM: 600852])(see “RetNet” in Web Resources).^4^^,^^5^ Following initial publications defining this locus^6^^,^^7^ a variant in *CA4* was implicated as the cause of adRP in families of South African origin, however pathogenicity of the reported variant has been questioned because it has a population frequency of 4% in healthy controls in northern Sweden.^8^, ^9^, ^10^ Subsequently reported *CA4* variants in individuals with RP were identified by targeted Sanger sequencing and do not fully exclude variants in other genes as a cause of disease (Table S1).

We investigated the cause of adRP in unsolved families, including the first pedigree (GC1, referred to as UK1) drawn up at Moorfields Eye Hospital over 35 years ago and the original Dutch family (W97-079, referred to as NL1) that showed linkage to the RP17 locus but lacked a mutation in *CA4*.^7^

Here, we report identification and characterization of complex SVs on Chr17q22, through whole-genome sequencing (WGS), as the genomic cause of adRP at the RP17 locus in a large number of families, including the families of South African origin. To explore a convergent mechanism of disease, we investigated the effect of RP17 SVs on three-dimensional (3D) chromatin organization that results in the compartmentalization of the genome into topologically associating domains (TADs) and the epigenetic landscape of the region. TADs are chromatin domains within the genome that facilitate enhancer promoter contacts within the nuclear 3D space.^11^ Disruption of TAD structures can lead to loss of chromosomal contact between regulatory regions and their target genes or the formation of novel active domains with ectopic contacts occurring between regulatory regions and a new target gene, resulting in pathogenic alterations in gene expression.^12^, ^13^, ^14^, ^15^ We demonstrate that altered TAD structure at the RP17 locus leads to ectopic retinal enhancer-gene interactions, consistent with a dominant gain of function. Our study highlights the pathogenicity of SVs that alter 3D chromatin organization and gene expression by rearranging TAD structures and the need to revisit rare Mendelian diseases for which genes and variants have not been substantiated in other cohorts.

## Material and Methods

### Study Cohort

The study was approved by the medical ethics committee of the ErasmusMC Rotterdam, Radboudumc Nijmegen, and Moorfields Eye Hospital and was performed in accordance with the principles of the World Medical Association Declaration of Helsinki. Informed consent was obtained from all participants or their legal representatives.

### Genetic Analyses

We performed SNP genotyping for index families NL1 and UK1 to define and refine the RP17 locus. Genomic DNA from affected individuals and their family members was analyzed by whole-exome sequencing (WES) and WGS. Sequence data was aligned to the Human Reference Genome build hg19. Variants were prioritized on the basis of a minor allele frequency (MAF) ≤0.0001 in gnomAD. SVs were called with ExomeDepth, Manta Structural Variant Caller, Canvas Copy Number Variant Caller, and Control-FREEC. Details of genotyping, sequencing, and analysis pipelines are provided in the Supplemental Material and Methods.

### Characterization and Validation of Structural Variants

SV breakpoint junctions were PCR amplified and validated with Sanger sequencing. Primer sequences and coordinates are listed in Table S2. SV breakpoint regions were assessed for the presence of microhomology and repetitive elements. To validate a triplicated region for UK-SV6, we performed quantitative real-time PCR (qPCR) on genomic DNA from affected individuals from family UK13 and unaffected controls (Supplemental Material and Methods).

### Clinical Analysis

Available clinical notes of cases for the pedigrees identified at Radboudumc, Moorfields Eye Hospital, University of Cape Town, and McGill University Health Centre were reviewed, as well as detailed retinal imaging, fundus autofluorescence, and optical coherence tomography. Age of onset is defined as the age at which symptoms were first experienced.

### Interrogation of the Genomic Region

We interrogated chromatin and genome regulation datasets to explore the epigenomic landscape of the region. Available datasets were obtained and analyzed via the UCSC genome browser (details of datasets used are provided in Supplemental Material and Methods).

### Reprogramming Fibroblasts into iPSCs and Differentiation into Photoreceptor Progenitor Cells and 3D Retinal Organoids

Fibroblasts were cultured from skin biopsies of two individuals with NL-SV1, one individual with UK-SV2, and five anonymous control individuals. Cell lines were reprogrammed into induced pluripotent stem cells (iPSCs) and differentiated into photoreceptor progenitor cells (PPCs) following the previously described 60 day protocol (Supplemental Material and Methods).^16^^,^^17^ 3D retinal organoids (ROs) were differentiated for UK-SV2 and controls, as previously described (Supplemental Material and Methods).^18^

### Preparation of Low Input Hi-C Libraries (Low-C)

Hi-C was performed on UK-SV2 and control 3D ROs via a low input protocol (Low-C) with few modifications (Supplemental Material and Methods).^19^ Two libraries per sample were sequenced for 200 million fragments in a 100 bp paired-end run on a NovaSeq 6000 (Illumina). Paired-end sequencing data was processed via Juicer^20^ and the Hi-C maps were created with a bin size with 10 kb resolution. Further information about the bioinformatics pipeline is detailed in Melo et al., 2020.^21^

### Expression Analysis of Genes and Enhancer RNA within the RP17 Locus

To assess expression of genes, we performed qPCR for different human tissues, including retina (Table S3 and Supplemental Material and Methods). Single-cell RNA sequencing data of human^22^ and primate^23^ retinal cell types were obtained and visualized via the Broad Institute Single Cell Portal (Supplemental Material and Methods).

cDNA was synthesized from total RNA extracted from PPCs, ROs, and fibroblasts. Differential expression of genes implicated in the SVs, and control housekeeping and retinal progenitor genes, was assessed by qPCR (Table S3 and Supplemental Material and Methods). We designed primers to the enhancer region containing multiple retinal transcription factor binding sites implicated in all SVs to analyze targeted enhancer RNA expression by qPCR (Table S3 and Supplemental Material and Methods).

## Results

### Refinement of the RP17 Locus in Two Unrelated adRP-Affected Families

**Figure 1:**
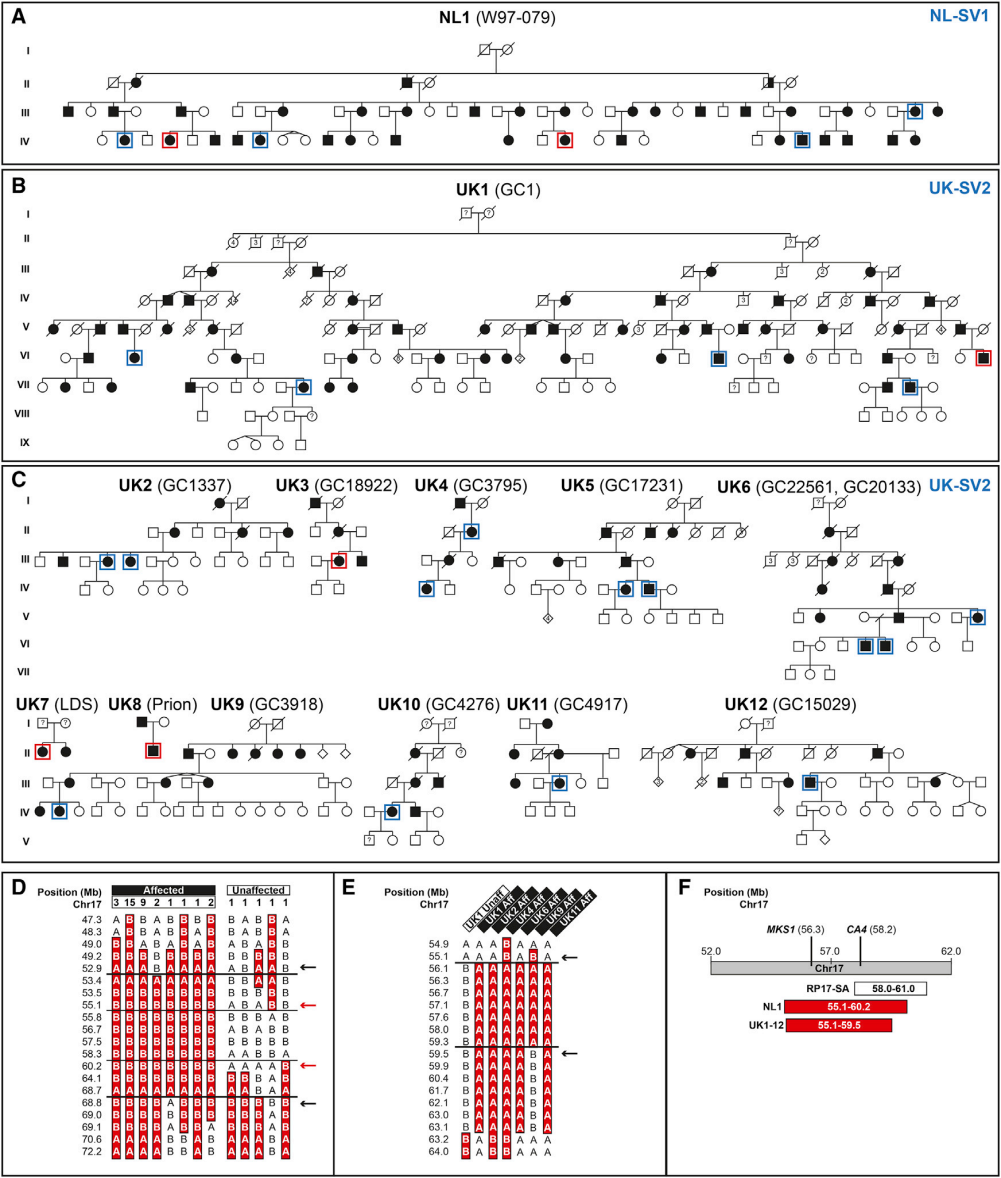
Mapping of the RP17 Locus in Two Unrelated Families (A) Pedigree of Dutch NL1 family. (B) Pedigree of UK1 family. (C) Pedigrees of additional UK families with the founder haplotype on Chr17q. WGS or WES was performed in individuals highlighted in blue or red, respectively. (D) SNP haplotyping results for NL1. The refined RP17 locus (rs8078110–rs9910672) is shared by all affected individuals (n = 35) and not present in unaffected individuals (n = 28, only individuals with recombination close to or refining the critical region are depicted) with a maximum LOD score of 15.0. The horizontal numbers represent the number of individuals with this haplotype. (E) UK founder haplotype refining the RP17 locus in UK families. Representative haplotypes from several unrelated families are shown with affected (aff) individuals compared to an unaffected (unaff) individual. Black lines and arrows indicate recombination events. Shared haplotype in individuals is shaded red. (F) Overlap of refined RP17 loci in UK, NL, and previously described SA families.^8^

The affected haplotype for a Dutch adRP-affected family (NL1) (Figure 1A) was previously mapped to a 7.18 Mb region spanning the RP17 locus on chromosome 17.^7^ The RP17 locus was refined to a 5.16 Mb interval by SNP haplotyping in an extended pedigree (Figure 1D and Supplemental Information). No rare coding or splice site heterozygous variants (MAF ≤ 0.0001) shared between affected individuals were found through WES. Subsequently, WGS was performed, and similarly, no rare candidate coding, splice site, intronic, or intergenic heterozygous single-nucleotide variants were identified (Table S4 and Supplemental Information).

In parallel, WES and WGS were performed for affected individuals from a genetically unexplained UK adRP-affected family (UK1) (Figure 1B). This also failed to identify a rare causative variant; however, a disease-associated haplotype on chromosome 17 was identified (Figure 1E, Table S5, and Supplemental Information). Interrogation of unsolved IRD sequence data generated through the UK IRDC, UCL-Ex, NIHR-Bioresource, and Genomics England identified other adRP probands that shared the same haplotype of Chr17 SNVs and established this as a founder haplotype in eleven additional UK adRP-affected families (Figure 1C). The adRP locus was refined to a 4.4 Mb interval on Chr17q22 (Figure 1E). This genomic interval overlaps the previously described RP17 locus in families of Dutch and South African origin (Figure 1F).

A missense variant in *CA4* [c.40C>T (p.Arg14Trp); GenBank: NM_000717.4] was previously described as the cause of adRP at the RP17 locus in families of South African origin.^8^ No rare coding, intronic, or upstream variants in *CA4* were identified in the Dutch and UK families.

### Identification of Structural Variants within the RP17 Locus

**Figure 2:**
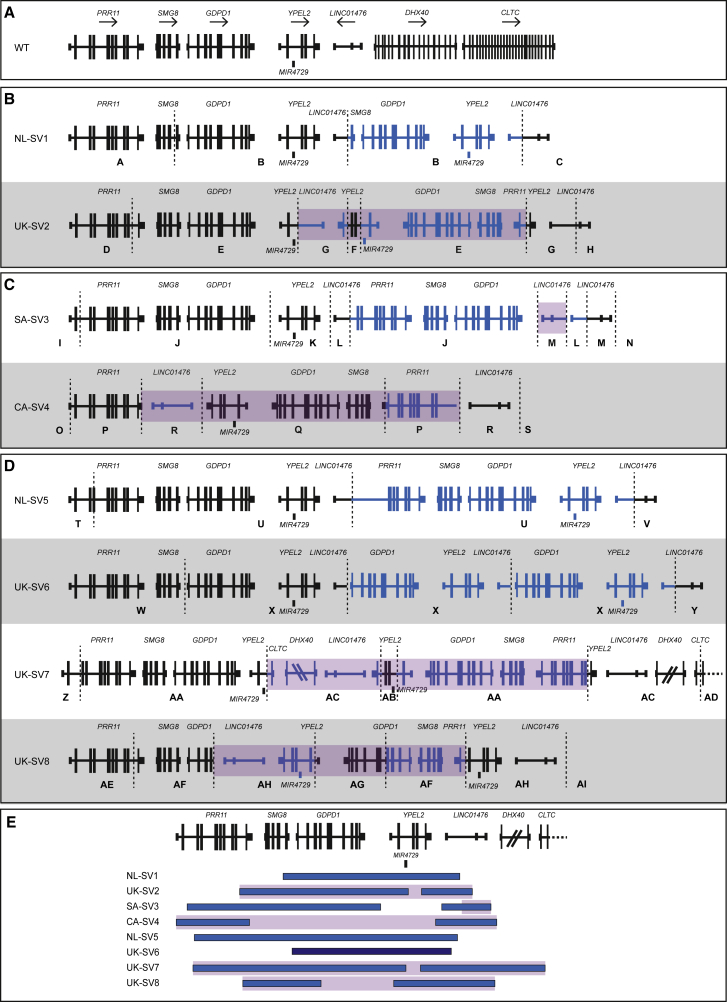
Overview of Structural Variants within the RP17 Locus in adRP-Affected Families Breakpoints are indicated with dashed lines. Blue segments represent duplicated or triplicated regions, whereas inversions are highlighted in purple. (A) Wild-type (WT) chromosomal organization. (B) Structural variants identified in NL1 (NL-SV1) and UK founder haplotype families (UK-SV2). (C) Structural variants identified in adRP-affected families that were previously linked to the RP17 locus; SA-SV3^8^ and CA-SV4 (unpublished data). (D) Structural variants found in a cohort of unsolved adRP-affected families; NL-SV5, UK-SV6, UK-SV7, and UK-SV8. Letters A–AI depict the genomic intervals for each SV used to analyze and annotate SV breakpoints. (E) Overview of all SV breakpoints identified in the RP17 locus. An overlapping genomic region that is duplicated or triplicated in all SVs was identified (chr17: 57,499,214–57,510,765) and is highlighted by a light blue vertical bar. The size of *DHX40* is reduced, and *CLTC* is partially shown for the purpose of this figure.

Next, we analyzed genome and exome data for copy number variants and SVs (Supplemental Information). In family NL1, WGS revealed a 226 kb duplication within the RP17 locus: chr17: 57,291,905_57,518,137dup (NL-SV1). This SV involves two duplicated genes (*GDPD1* [MIM: 616317] and *YPEL2* [MIM: 609723]), an intragenic microRNA (*MIR4729*), and partial duplication of *SMG8* (MIM: 613175) and the long non-coding RNA *LINC01476*. The duplication creates a breakpoint junction (chr17: g.57,518,137–57,291,905) specific for the mutated allele in NL1 (Figures 2A, 2B, and S1), which was used to confirm segregation of the SV with the adRP phenotype in this family. No overlapping SVs in the RP17 locus were observed in WES of ~7,500 individuals without retinal disease generated in-house at the Department of Human Genetics, Radboudumc.

For the twelve UK RP17 founder haplotype families, WGS revealed a duplicated inversion: chr17: 57,456,098–57,468,960delins57,275,839_57,559,114inv (UK-SV2) (Figure 2B). The SV was characterized, and breakpoint junctions were validated (Figure S1 and Supplemental Information). This SV involved four coding genes (*PRR11* [MIM: 615920], *SMG8*, *GDPD1*, and *YPEL2*) and two non-coding RNA genes (*MIR4729* and *LINC01476*) (Figure 2B). UK-SV2 segregated with adRP in all families for which DNA was available for analysis. UK-SV2 was absent in WGS control genome data generated for 58,000 UK individuals (Genomics England).

### Different Structural Variants within the RP17 Locus in Multiple adRP-Affected Families

These data prompted us to investigate whether SVs were present in the two original South African families (SA1 and SA2) that were linked to the RP17 locus (Figure S2A).^6^^,^^8^ In addition, a Canadian adRP-affected family (CA1) was also mapped to the RP17 locus (unpublished data, Figure S2B). WGS was performed for affected individuals from these families, and inversion duplication events were identified in all samples analyzed (Figure 2C). In SA1 and SA2, an identical SV, SA-SV3, was revealed, suggesting this is a founder variant in this population. SA-SV3 was also found by breakpoint PCR in two additional families of South African origin (SA3 and SA4), confirming the founder effect (Figure S2A). In the Canadian family, a different inversion duplication event was identified, CA-SV4. SA-SV3 and CA-SV4 breakpoints were characterized and validated (Figure S1), and segregation of the SVs with the adRP phenotype was confirmed.

Our data suggested that SVs at the RP17 locus are an important cause of adRP. Therefore, WGS and WES data for genetically unexplained adRP-affected families were analyzed for SVs within this locus. In four unrelated families of Dutch or UK origin, four additional unique complex SVs were discovered (Figures 2D and S2C). For individuals that had only undergone WES, we performed WGS to determine the breakpoint junctions and identify potential inversions or other SVs. In all families, breakpoints were validated, and segregation analysis was performed where possible. Triplication for UK-SV6 was confirmed by qPCR in family UK13 (Figure S3 and Supplemental Information).

Details of all SVs identified in this study are shown in Table S6, Figure 2, and Figure S4, and an overview of SV-specific breakpoint junctions is shown in Figure S1. All RP17 SVs share a common duplicated (or triplicated) region of 11.5 kb and harbor unique breakpoints disrupting the genomic region spanning *YPEL2* to *LINC01476* (chr17: 57,499,214–57,510,765) (Figure S4). We analyzed all breakpoint junction sequences to investigate the potential mechanism(s) that created RP17 SVs. No single mechanism could account for the RP17 SVs because a combination of (micro)homology-mediated repair and non-homologous end joining events were identified (Table S7 and S8, Figure S5, and Supplemental Information).

### Consistent Autosomal-Dominant Retinitis Pigmentosa Phenotype for RP17-Affected Families

The SVs identified were fully penetrant in all families. Available clinical data are presented in Table S9. Twenty-four affected individuals from seventeen pedigrees were evaluated. There is significant correlation of phenotype across all genotypes with relatively mild disease, decreased visual acuity, visual field constriction, nyctalopia, and slow progression consistent with adRP. Many affected individuals have preserved central visual function and acuity until the 6^th^–7^th^ decade. Foveal sparing and cystoid macular edema were a common finding in individuals with UK-SV2. On the basis of a small number of affected individuals (n = 2), UK-SV6 (with a triplicated SV) may be associated with an earlier age of onset and more severe phenotype (Figure S6).

### Topologically Associating Domain Structure and Epigenetic Landscape of the RP17 Genomic Region

All of the RP17 SVs lead to disruption of the genomic region spanning *YPEL2* to *LINC01476* (Figure 2E). SVs that interfere with genome structure can have distinct effects on gene regulation depending on the type and extent of the SV and landscape of the genomic region.^15^ TADs are separated by boundaries, regions of low chromatin interaction that insulate the regulatory activities of neighboring TADs. The transcription factor CTCF (CCTC-binding factor) typically binds in these regions where it plays a pivotal role in the maintenance of boundaries. SVs can cause loss of function by disconnecting enhancers from their target genes; however, disruption of TAD structures and boundaries can also exert a gain-of-function effect. Deletions, for example, can lead to the fusing of two previously separated TADs (TAD-fusion), inversions can result in the exchange of regulatory material between TADs (TAD-shuffling), whereas duplications can give rise to the generation of novel domains, so-called neo-TADs.^12^^,^^13^ In each case, SVs result in the generation of ectopic contacts of enhancers with the promoters of novel target genes resulting in aberrant gene activation. The human limb malformations caused by SVs that alter the CTCF-associated boundary of the WNT6/IHH/EPHA4/PAX3 locus are a prominent example. The SVs result in ectopic interactions between *EPHA4* (MIM: 602188) limb enhancers and the neighboring developmental genes that are normally insulated, driving ectopic expression in the limb.^14^ Similarly, the deletion of a CTCF site located between the *Xist* (MIM: 314670) and *Tsix* (MIM: 300181) TADs on the X chromosome resulted in a novel domain by fusion of the adjacent TADs (fused-TAD).^24^ As a consequence, previously insulated enhancers activated genes in the adjacent TAD, leading to the dysregulation of these genes.

**Figure 3:**
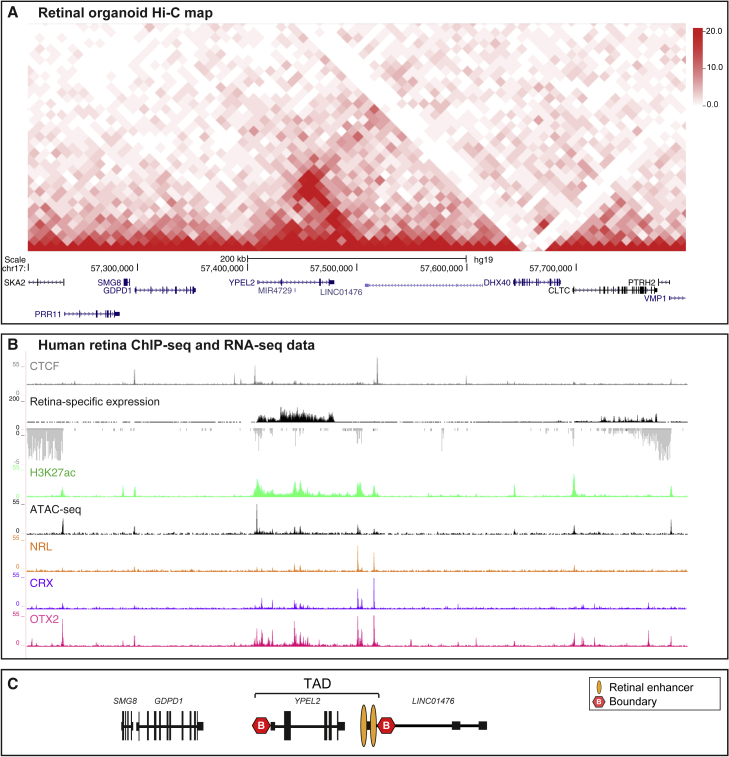
*YPEL2* is Located within a Structured Active Compartment that Contains Retinal-Specific Enhancers (A) The TAD landscape of the genomic region disrupted by the RP17 SVs. Hi-C map of control retinal organoids revealed a structured domain containing *YPEL2*. (B) *YPEL2* TAD boundaries correspond with CTCF sites identified in human retina. Analysis of RNA-seq and assay for transposase-accessible chromatin using seqencing (ATAC-seq) data across the *YPEL2* region shows *YPEL2* retinal expression and an accessible chromatin configuration. Analysis of H3K27Ac ChIP-seq data in the same region revealed several active enhancers located within the *YPEL2* TAD, which are enriched for retinal transcription factor binding sites, including NRL, CRX, and OTX2.^25^ These enhancers were located 5′ of the CTCF boundary site within *LINC01476.* (C) Schematic representation of the *YPEL2* TAD structure.

Hi-C data were not available for human retina, and therefore, we generated Hi-C maps of control human 3D ROs to obtain maps of the chromatin organization of our region of interest. Hi-C revealed a structured domain containing *YPEL2* (*YPEL2* TAD) flanked by less structured neighboring domains (Figure 3A). CTCF binding is present on both boundaries (Figure 3B) supporting the TAD structure at this locus. CTCF ChIA-PET data highlighted interactions between the CTCF binding sites at the 5′ and the 3′ boundary of the *YPEL2* TAD (Figure S7B).

Assay for transposase-accessible chromatin using seqencing (ATAC-seq) data from human retina show that the chromatin in the *YPEL2* TAD is accessible, and H3K27Ac ChIP-seq data revealed that there are several active enhancers located within the *YPEL2* TAD that are expected to drive *YPEL2* expression in the retina (Figure 3B).^25^ Importantly, the *YPEL2* TAD harbors two regions of active enhancers with binding sites for transcription factor (TFs) known to be required for photoreceptor function, including NRL, CRX, and OTX2 (Figure 3B). NRL is a TF that is preferentially expressed in rod photoreceptors. These TF binding sites correlated with H3K27Ac and ATAC-seq peaks in retina. The published GeneHancer dataset shows that these regulatory elements have interactions with the *YPEL2* promoter (Figure S7C).^26^ Collectively, these analyses revealed that *YPEL2* is located within an active compartment that contains retinal-specific enhancers (Figure 3C).

### Expression of *YPEL2* and *GDPD1*

Expression of *YPEL2* and *GDPD1* was assessed by qPCR in multiple healthy human tissues, including retina (Figure S8). *YPEL2* is ubiquitously expressed in the tissues studied, including retina, with highest relative expression in brain. Single-cell retina RNA-seq datasets revealed *YPEL2* is expressed at higher levels in rod photoreceptor cells, which is the primary cell type affected in retinitis pigmentosa, compared to cone photoreceptors (Figure S8).^23^ *GDPD1* is detected at low expression in all tissue types but has higher expression in testis and the brain. These data support the hypothesis that *YPEL2* expression is regulated by retinal enhancers within the *YPEL2* TAD.

### RP17 SVs Create New Topologically Associating Domains and Ectopic Enhancer-Gene Interactions

**Figure 4:**
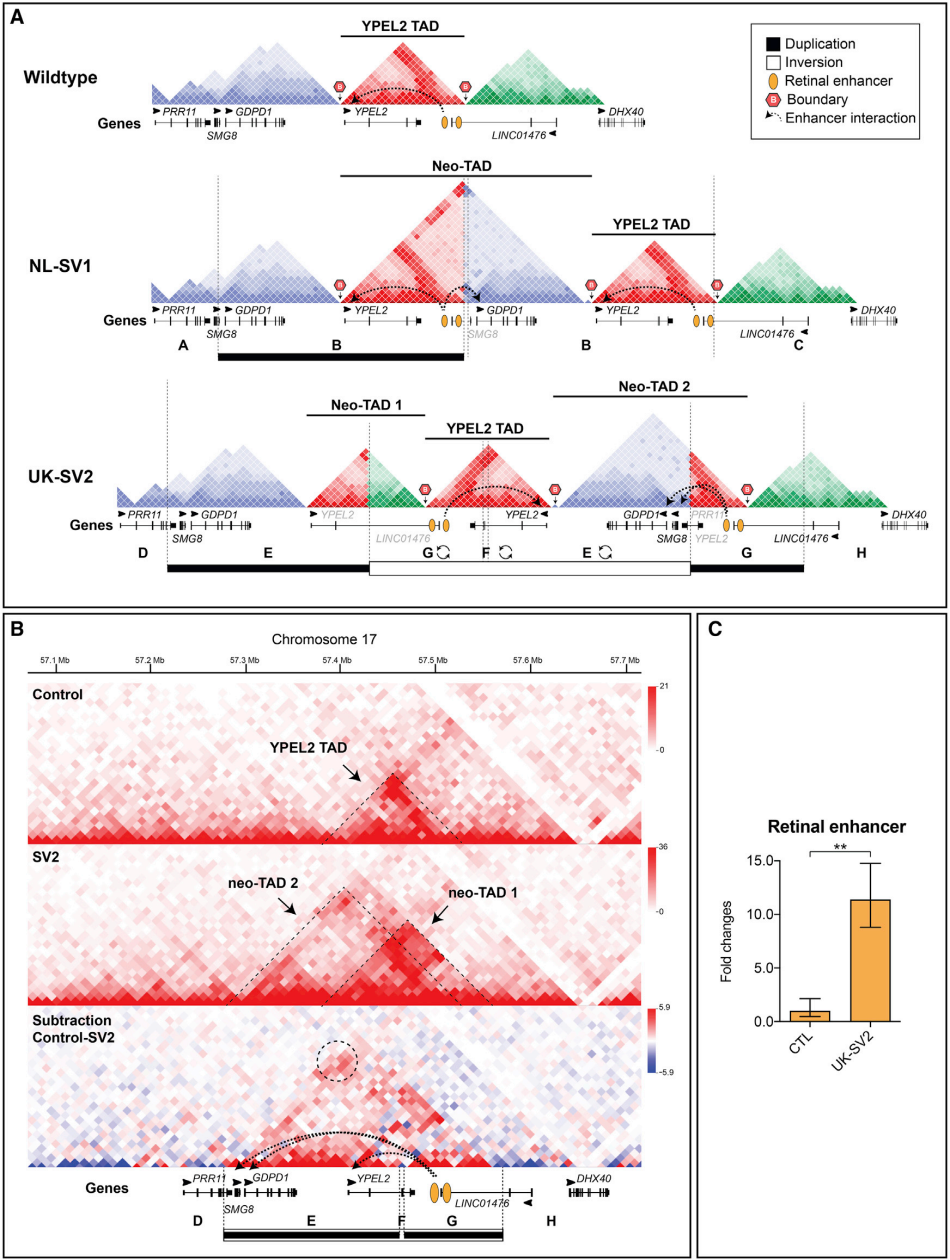
RP17 SVs Create Novel Domains (neo-TADs) and Hyper-activation of Retinal Enhancers (A) Schematic modeling of the genome architecture spanning the RP17 region using Hi-C maps. The wild-type Hi-C map derived from neuronal tissue shows a TAD with CTCF boundaries containing *YPEL2* and retinal enhancers, flanked by unstructured domains. TAD models of NL-SV1 and UK-SV2 (dotted vertical lines represent SV breakpoints) predict the formation of neo-TADs and ectopic interactions of the retinal enhancer with *GDPD1*. (B) Hi-C performed on retinal organoids (ROs) derived from control (top) and RP17 UK-SV2 individuals (bottom) (10 kb resolution; raw count map). The chromatin organization in control ROs shows the *YPEL2* TAD (indicated by dashed lines). Two novel domains (neo-TAD 1 and 2) are visible in the UK-SV2 ROs, and neo-TAD 2 allows ectopic retinal enhancer contacts to *GDPD1* and *SMG8*. The dashed circle indicates the strong chromatin contact between retinal enhancers and the *GDPD1* promoter. (C) qPCR revealed significantly upregulated retinal enhancer RNA expression in UK-SV2 ROs compared to controls (n = 3, mean ± standard error of the mean, **p ≤ 0.01).

Using the wild-type retinal organoid Hi-C map, we modeled the TAD boundaries, CTCF site orientation, and retinal TF binding site positions for each unique RP17 SV (Figures 4A and S9). In NL-SV1, the duplication contains part of the *YPEL2* TAD, the boundary to the neighboring region and *GDPD1*. This results in the creation of a neo-TAD that now contains the previously separated *YPEL2* enhancers and *GDPD1* in one domain. To directly investigate the effect of the SVs in retinal cells, we reprogrammed dermal fibroblasts from UK-SV2 to iPSC and differentiated to 3D ROs, thus creating an *in vitro* model (Supplemental Information). In this case, the duplicated regions are also inverted. Hi-C of RP17 ROs (UK-SV2) revealed the creation of two neo-TADs compared to control ROs (Figure 4B). The rearrangement of CTCF sites caused by the SV creates boundaries for two novel domains (neo-TAD 1 and 2) where neo-TAD 2 contains a duplicated copy of *GDPD1* and *SMG8* and the retinal enhancers, confirming the modeling for this SV (Figure 4A). Furthermore, on the basis of our predictions, neo-TADs are created in each of the RP17 cases and *GDPD1* is predicted to gain ectopic access to the retinal-specific enhancers (Figures 4A and S9). Therefore, the potential convergent mechanism for retinal degeneration is transcriptional activation and expression of *GDPD1* through juxtaposition of retinal TF binding sites within active compartments bounded by CTCF sites. This model would also fit with a dominant gain-of-function mechanism of disease.

Next, we assessed retinal enhancer expression in control and UK-SV2 ROs by enhancer RNA qPCR (Supplemental Information). A significant increase of the retinal enhancer was detected in RP17 ROs (Figure 4C), demonstrating that this transcriptionally active retinal enhancer in the neo-TAD could drive retinal expression of *GDPD1*.

### Differential Expression of *GDPD1* in RP17 iPSC-Derived Photoreceptor Precursors and 3D Retinal Organoids

**Figure 5:**
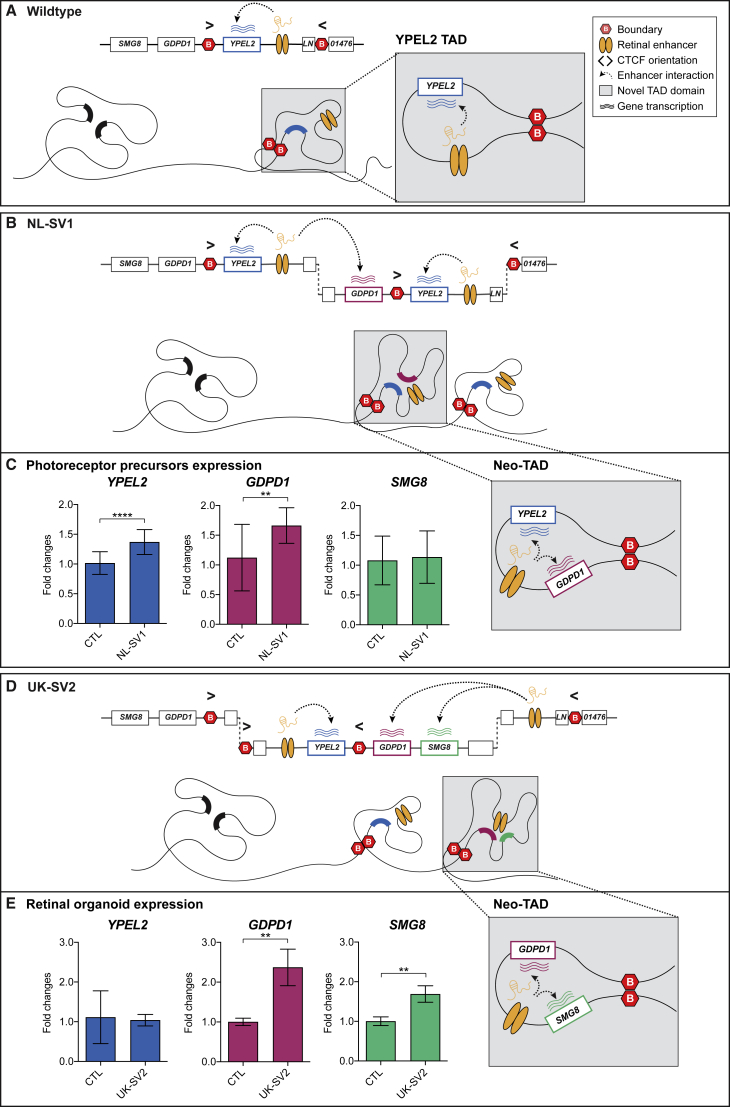
Convergent Mechanism of Ectopic Retinal Enhancer-*GDPD1* Interaction Caused by RP17 SVs (A) In wild-type genomic context, *YPEL2* expression in retina is driven by retinal enhancers in a TAD with CTCF boundaries. Neighboring genes are insulated from retinal enhancer activation. (B) The NL-SV1 duplication creates a neo-TAD with a full-length copy of *YPEL2*, *GDPD1*, and the retinal enhancers. This enables retinal-specific enhancers to ectopically interact with *GDPD1*, which drives its misexpression. (C) qPCR analysis of photoreceptor precursor cells (PPCs) revealed a significant upregulation of *GDPD1* in NL-SV1 PPCs compared to controls. (D) The UK-SV2 duplication and inversion creates a neo-TAD with a full-length copy of *GDPD1* and *SMG8* and the retinal enhancers bounded by CTFC sites. (E) qPCR analysis ROs revealed a significant upregulation of *GDPD1* expression in UK-SV2 ROs compared to controls (n = 3 independent ROs, mean ± standard error of the mean, **p ≤ 0.01, ****p ≤ 0.0001).

Our experimental data and modeling predict *GDPD1* enters a neo-TAD with retinal enhancers in all RP17 SVs. An extra copy of *YPEL2* enters the neo-TAD of NL-SV1, and *SMG8* enters this domain in UK-SV2 (Figures 5 and S9).

To experimentally validate the consequence of RP17 SVs in genomic and cellular context, we performed qPCR to assess differential expression in PPCs (NL-SV1) and ROs (UK-SV2). The expression of *GDPD1*, *YPEL2*, and *SMG8* was compared to controls (Supplemental Information).

In both experimental models, the expression of *GDPD1* was significantly increased compared to controls. *YPEL2* was increased in NL-SV1 only (Figure 5C), whereas *SMG8* was increased in UK-SV2 (Figure 5E), which correlates with our TAD modeling and Hi-C experimental data for UK-SV2 ROs (Figured 5B, 5D, and S9). To further explore the tissue-specific effect of this transcriptional upregulation, we performed the same qPCR assays on fibroblasts of the same individuals. None of these genes had increased expression in affected individuals compared to controls (data not shown).

## Discussion

Previous genetic studies of adRP-affected families mapping to the RP17 locus have implicated missense variants in *CA4* as the cause of disease or have been unable to confirm pathogenicity.^7^^,^^8^ Here, we describe the discovery of SVs as the cause of adRP at the RP17 locus in a large number of families, suggesting this is a previously unrecognized major locus for adRP. Our results show how complex rearrangements can result in the disruption of 3D genome architecture, the re-wiring of enhancer-promoter interactions, and consequent gene misexpression.

Following the identification of SVs in NL1 and UK1 via short-read WGS, our search for similar complex SVs in the RP17 genomic interval of genetically unexplained adRP-affected families identified six other complex SVs that segregated with disease. SVs are a major source of normal variation in the human genome and are often benign;^27^^,^^28^ however, none of the RP17 SVs are found in the population database gnomAD^29^ or the Database of Genomic Variants (DGV).^30^ Although overlapping canonical SVs (deletions and duplications) have been identified, they do not have breakpoints within the *YPEL2*-*LINC01476* region, as observed for all RP17 SVs reported in this study. This is in line with observations that different SVs can have different consequences depending on the characteristics of specific SVs in local 3D chromatin and epigenetic context.^31^^,^^32^

Base level resolution of breakpoint junctions and interrogation of the DNA sequence signatures revealed the mechanisms of the chromosomal rearrangements. Repetitive elements are key factors in facilitating unequal crossover of genomic segments or providing microhomology that induces fork stalling and subsequent template switching.^33^^,^^34^ Consistent with this model, repetitive elements were present in the flanking sequences of breakpoint junctions. In addition, microhomology, larger stretches of homology, and small insertions-deletions were found at all breakpoints.^34^, ^35^, ^36^ Therefore, repetitive elements may explain why the RP17 locus is prone to such structural variation, which is supported by the presence of breakpoint “hotspots,” as seen in *LINC01476* intron 2 and *YPEL2* intron 4; some breakpoints only differ by a small number of base pairs (e.g., for UK-SV2 and UK-SV7).

None of the genes implicated in the RP17 SVs have been previously associated with retinal disease. *YPEL2* is expressed in retina, and single-cell RNA sequencing of human and primate retina revealed expression in photoreceptors; the highest expression was in rod photoreceptors.^22^^,^^23^ Although the function of YPEL2 in the retina is unknown, we show that retinal expression is controlled by a number of retinal TF binding sites, including NRL, which is predominantly expressed in rod photoreceptors. Furthermore, Hi-C data show that *YPEL2* and the retinal enhancer binding sites are insulated from the surrounding region in a structured *YPEL2* TAD in control ROs and other tissues.

Hi-C analyses of UK-SV2 ROs revealed the generation of neo-TADs, and altered structure and repositioning of the boundaries enabled *GDPD1* promoter-retinal enhancer contacts and consequent *GDPD1* misexpression in the retina. The molecular disease mechanism in these cases is similar to the reported duplications at the SOX9/KCNJ2 locus.^13^ As described for the rearrangements reported here, the duplications at the *SOX9* (MIM: 608160) locus also encompass a regulatory domain (of *SOX9*), a boundary (between the *SOX9* and the *KCNJ2* [MIM: 600681] TADs), and the neighboring gene (*KCNJ2*). This results in the formation of a neo-TAD containing the *SOX9* regulatory elements and the new target gene (*KCNJ2*) that are now free to interact. In the *SOX9* case, this leads to misexpression of *KCNJ2* in a *SOX9* pattern and consecutive limb malformation, whereas in the RP-affected individuals, the interaction of *GDPD1* with *YPEL2* enhancers leads to misexpression in the retina. However, in some of the RP-affected individuals, such as UK-SV2, the situation is more complex because the duplications are inverted. Inversions can lead to the exchange of regulatory material from one end of the breakpoint to the other (also called TAD-shuffling).^12^ In UK-SV2, the duplication creates two neo-TADs, but the content is reorganized by the inversion. Again, *GDPD1* and retinal enhancers are brought together in one new TAD. Thus, the pathogenetic principle remains the same because all the RP17 SVs are predicted to create new TADs allowing access of the retinal enhancers to *GDPD1.* This suggests that increased expression of *GDPD1* in photoreceptors is the convergent mechanism of disease. Consistent with this hypothesis, PPCs from NL-SV1 and ROs from UK-SV2 showed significant increased expression of *GDPD1* in RP17-affected families with different SVs compared to controls. In UK-SV2 ROs, an increased expression of *SMG8*, which is also introduced into the active neo-TAD of UK-SV2, was observed. Conversely, *YPEL2* shows upregulation in NL-SV1, which is in line with the complete duplication of *YPEL2* in NL-SV1. Importantly, qPCR provided evidence for the increased expression of the retinal enhancer in UK-SV2 ROs and TF binding sites for NRL, which is preferentially expressed in rod photoreceptors, the primary cell type affected in RP.

Although increased expression of *SMG8*, *YPEL2*, or the retinal enhancer cannot be excluded from contributing to the phenotype in individual families, these experimental data support the hypothesis of a convergent mechanism of *GDPD1* entry into the active neo-TAD with retinal enhancers for all eight complex RP17 SVs. This is further supported by the observation that the two affected individuals in family UK13, who had an earlier age of onset and more severe phenotype compared to all other families, have a triplication (UK-SV6) where two copies of *GDPD1* are predicted to enter the active neo-TAD.

Our data implicate increased retinal expression of *GDPD1* as a dominant gain-of-function mechanism leading to adRP. *GDPD1* encodes a glycerophosphodiesterase, which can hydrolyze lysophosphatidylcholine (lyso-PC) to lysophosphatidic acid (LPA)^37^ with lysophospholipase D (lysoPLD) activity on various lysophospholipids.^38^ *GPDP1* is detected at low expression in the healthy retina, and therefore, increased expression of *GDPD1* could lead to dysregulation of lipid metabolism, which is known to be critical for photoreceptor function, although the exact mechanisms of photoreceptor cell death are not known.^39^, ^40^, ^41^ Disruption of lipid metabolism leading to adRP, combined with the adult age of onset, opens avenues for therapeutic intervention to preserve vision by restoring lipid homeostasis.

## Declaration of Interests

The authors declare no competing interests.

## Data and Code Availability

Extensive data are presented in the Supplemental Information to enable others to perform similar studies and replicate our findings. WES and WGS data have not been deposited in a public repository because of consent and ethical considerations. Hi-C data are available on request.

## Web Resources

GeneReviews, Fahim, A.T., Daiger, S.P., and Weleber, R.G. (2017). Nonsyndromic Retinitis Pigmentosa Overview, https://www.ncbi.nlm.nih.gov/books/NBK1417/
RetNet, https://sph.uth.edu/RetNet

## Supplemental Data

Document S1. Figures S1–S9, Tables S1–S8, and Supplemental Material and Methods

Table S9. Clinical Findings

Document S2. Article plus Supplemental Information
