# Supplementary material for: Structural Variants Create New Topological-Associated Domains and Ectopic Retinal Enhancer-Gene Contact in Dominant Retinitis Pigmentosa
Source: Am J Hum Genet. 2020 Oct 5;107(5):802–14. doi: 10.1016/j.ajhg.2020.09.002 (PMC7675008; doi:10.1016/j.ajhg.2020.09.002)
Supplement: Document S2. Article plus Supplemental Information [file mmc3.pdf]

ARTICLE

Structural Variants Create New Topological-Associated Domains and Ectopic Retinal Enhancer-Gene Contact in Dominant Retinitis Pigmentosa

Suzanne E. de Bruijn,[1,2,26] Alessia Fiorentino,[3,4,5,26] Daniele Ottaviani,[3] Stephanie Fanucchi,[6] Uirá S. Melo,[7,8] Julio C. Corral-Serrano,[3] Timo Mulders,[2,9] Michalis Georgiou,[3,10] Carlo Rivolta,[11,12,13] Nikolas Pontikos,[3,4,5] Gavin Arno,[3,4,5,10] Lisa Roberts,[14] Jacquie Greenberg,[14] Silvia Albert,[1] Christian Gilissen,[1] Marco Aben,[1] George Rebello,[14] Simon Mead,[15] F. Lucy Raymond,[16,17] Jordi Corominas,[1] Claire E.L. Smith,[18] Hannie Kremer,[1,2,19] Susan Downes,[4,5,20] Graeme C. Black,[4,5,21] Andrew R. Webster,[3,4,5,10] Chris F. Inglehearn,[4,5,18] L. Ingeborgh van den Born,[22] Robert K. Koenekoop,[23] Michel Michaelides,[3,4,5,10] Raj S. Ramesar,[14] Carel B. Hoyng,[2,9] Stefan Mundlos,[7,8] Musa M. Mhlanga,[1,6,24,25] Frans P.M. Cremers,[1,2] Michael E. Cheetham,[3,4,5] Susanne Roosing,[1,2,27,*] and Alison J. Hardcastle[3,4,5,27]

Summary

The cause of autosomal-dominant retinitis pigmentosa (adRP), which leads to loss of vision and blindness, was investigated in families lacking a molecular diagnosis. A refined locus for adRP on Chr17q22 (RP17) was delineated through genotyping and genome sequencing, leading to the identification of structural variants (SVs) that segregate with disease. Eight different complex SVs were characterized in 22 adRP-affected families with >300 affected individuals. All RP17 SVs had breakpoints within a genomic region spanning *YPEL2* to *LINC01476*. To investigate the mechanism of disease, we reprogrammed fibroblasts from affected individuals and controls into induced pluripotent stem cells (iPSCs) and differentiated them into photoreceptor precursor cells (PPCs) or retinal organoids (ROs). Hi-C was performed on ROs, and differential expression of regional genes and a retinal enhancer RNA at this locus was assessed by qPCR. The epigenetic landscape of the region, and Hi-C RO data, showed that *YPEL2* sits within its own topologically associating domain (TAD), rich in enhancers with binding sites for retinal transcription factors. The Hi-C map of RP17 ROs revealed creation of a neo-TAD with ectopic contacts between *GDPD1* and retinal enhancers, and modeling of all RP17 SVs was consistent with neo-TADs leading to ectopic retinal-specific enhancer-*GDPD1* accessibility. qPCR confirmed increased expression of *GDPD1* and increased expression of the retinal enhancer that enters the neo-TAD. Altered TAD structure resulting in increased retinal expression of *GDPD1* is the likely convergent mechanism of disease, consistent with a dominant gain of function. Our study highlights the importance of SVs as a genomic mechanism in unsolved Mendelian diseases.

Introduction

Despite recent advances in next-generation sequencing, approximately 30%–40% of individuals with inherited retinal diseases (IRDs) lack a molecular diagnosis. This is probably due to a combination of rare novel disease genes, which require large cohorts for validation, and previously intractable mutation classes, such as intronic variants, structural variants (SVs), and variants in regulatory regions.[1,2]

[1]Department of Human Genetics, Radboud University Medical Center, Nijmegen, 6500 HB, the Netherlands; [2]Donders Institute for Brain Cognition and Behaviour, Radboud University Medical Center, Nijmegen, 6500 HB, the Netherlands; [3]UCL Institute of Ophthalmology, London, EC1V 9EL, UK; [4]UK Inherited Retinal Disease Consortium; [5]Genomics England Clinical Interpretation Partnership; [6]Gene Expression and Biophysics Group, Division of Chemical, Systems and Synthetic Biology, Department of Integrative Biomedical Science, Institute for Infectious Disease & Molecular Medicine, Faculty of Health Sciences, University of Cape Town, Cape Town, 7935, South Africa; [7]Max Planck Institute for Molecular Genetics, RG Development & Disease, Berlin, 14195, Germany; [8]Institute for Medical and Human Genetics, Charité – Universitätsmedizin, Berlin, 10117, Germany; [9]Department of Ophthalmology, Radboud University Medical Center, Nijmegen, 6500 HB, the Netherlands; [10]Moorfields Eye Hospital, London, EC1V 2PD, UK; [11]Department of Genetics and Genome Biology, University of Leicester, Leicester, LE1 7RH, UK; [12]Clinical Research Center, Institute of Molecular and Clinical Ophthalmology Basel (IOB), Basel, 4031, Switzerland; [13]Department of Ophthalmology, University Hospital Basel, Basel, 4001, Switzerland; [14]University of Cape Town/MRC Genomic and Precision Medicine Research Unit, Division of Human Genetics, Department of Pathology, Institute of Infectious Disease and Molecular Medicine, Faculty of Health Sciences, University of Cape Town, Cape Town, 7935, South Africa; [15]MRC Prion Unit at UCL, UCL Institute of Prion Disease, London, W1W 7FF, UK; [16]NIHR BioResource, Cambridge University Hospitals, Cambridge, CB2 0QQ, UK; [17]Department of Medical Genetics, Cambridge Institute for Medical Research, University of Cambridge, Cambridge, CB2 OXY, UK; [18]Division of Molecular Medicine, Leeds Institute of Medical Research, University of Leeds, Leeds, LS2 9JT, UK; [19]Department of Otorhinolaryngology, Radboud University Medical Center, Nijmegen, 6500 HB, the Netherlands; [20]Oxford Eye Hospital, Oxford University Hospitals NHS Trust and Nuffield Laboratory of Ophthalmology, University of Oxford, Oxford, OX3 9DU, UK; [21]Manchester Centre for Genomic Medicine, St. Mary's Hospital, Manchester, M13 9WL, UK; [22]The Rotterdam Eye Hospital, Rotterdam, 3011 BH, the Netherlands; [23]Department of Paediatric Surgery, Human Genetics and Ophthalmology, McGill University, Montréal, QC H4A 3J1, Canada; [24]Gene Expression and Biophysics Unit, Instituto de Medicina Molecular, Faculdade de Medicina Universidade de Lisboa, Lisbon, 1649-028, Portugal; [25]Epigenomics & Single Cell Biophysics Group, Radboud Institute for Molecular Life Sciences (RIMLS), Radboud University, Nijmegen, 6525 GA, the Netherlands
[26]These authors contributed equally
[27]These authors contributed equally
*Correspondence: susanne.roosing@radboudumc.nl
https://doi.org/10.1016/j.ajhg.2020.09.002.
© 2020 The Authors. This is an open access article under the CC BY license (http://creativecommons.org/licenses/by/4.0/).

The most common form of IRD is retinitis pigmentosa (RP [MIM: 268000]), which is genetically heterogeneous, with a prevalence of 1 in 4,000.[3] RP is defined as a retinal degeneration that primarily affects rod photoreceptors, resulting in night blindness and progressive loss of peripheral vision, often progressing into the central retina and affecting cone photoreceptors, leading to severe visual impairment or blindness (see "Nonsyndromic Retinitis Pigmentosa Overview" in Web Resources). Autosomal-dominant RP (adRP) accounts for 25%–40% of cases, depending on the population studied, and has been associated with mutations in 30 genes, including *CA4* (MIM: 114760) on Chr17q23.1 (RP17 [MIM: 600852])(see "RetNet" in Web Resources).[4,5] Following initial publications defining this locus[6,7] a variant in *CA4* was implicated as the cause of adRP in families of South African origin, however pathogenicity of the reported variant has been questioned because it has a population frequency of 4% in healthy controls in northern Sweden.[8–10] Subsequently reported *CA4* variants in individuals with RP were identified by targeted Sanger sequencing and do not fully exclude variants in other genes as a cause of disease (Table S1).

We investigated the cause of adRP in unsolved families, including the first pedigree (GC1, referred to as UK1) drawn up at Moorfields Eye Hospital over 35 years ago and the original Dutch family (W97-079, referred to as NL1) that showed linkage to the RP17 locus but lacked a mutation in *CA4*.[7]

Here, we report identification and characterization of complex SVs on Chr17q22, through whole-genome sequencing (WGS), as the genomic cause of adRP at the RP17 locus in a large number of families, including the families of South African origin. To explore a convergent mechanism of disease, we investigated the effect of RP17 SVs on three-dimensional (3D) chromatin organization that results in the compartmentalization of the genome into topologically associating domains (TADs) and the epigenetic landscape of the region. TADs are chromatin domains within the genome that facilitate enhancer promoter contacts within the nuclear 3D space.[11] Disruption of TAD structures can lead to loss of chromosomal contact between regulatory regions and their target genes or the formation of novel active domains with ectopic contacts occurring between regulatory regions and a new target gene, resulting in pathogenic alterations in gene expression.[12–15] We demonstrate that altered TAD structure at the RP17 locus leads to ectopic retinal enhancer-gene interactions, consistent with a dominant gain of function. Our study highlights the pathogenicity of SVs that alter 3D chromatin organization and gene expression by rearranging TAD structures and the need to revisit rare Mendelian diseases for which genes and variants have not been substantiated in other cohorts.

Material and Methods

Study Cohort

The study was approved by the medical ethics committee of the ErasmusMC Rotterdam, Radboudumc Nijmegen, and Moorfields Eye Hospital and was performed in accordance with the principles of the World Medical Association Declaration of Helsinki. Informed consent was obtained from all participants or their legal representatives.

Genetic Analyses

We performed SNP genotyping for index families NL1 and UK1 to define and refine the RP17 locus. Genomic DNA from affected individuals and their family members was analyzed by whole-exome sequencing (WES) and WGS. Sequence data was aligned to the Human Reference Genome build hg19. Variants were prioritized on the basis of a minor allele frequency (MAF) $\leq$ 0.0001 in gnomAD. SVs were called with ExomeDepth, Manta Structural Variant Caller, Canvas Copy Number Variant Caller, and Control-FREEC. Details of genotyping, sequencing, and analysis pipelines are provided in the Supplemental Material and Methods.

Characterization and Validation of Structural Variants

SV breakpoint junctions were PCR amplified and validated with Sanger sequencing. Primer sequences and coordinates are listed in Table S2. SV breakpoint regions were assessed for the presence of microhomology and repetitive elements. To validate a triplicated region for UK-SV6, we performed quantitative real-time PCR (qPCR) on genomic DNA from affected individuals from family UK13 and unaffected controls (Supplemental Material and Methods).

Clinical Analysis

Available clinical notes of cases for the pedigrees identified at Radboudumc, Moorfields Eye Hospital, University of Cape Town, and McGill University Health Centre were reviewed, as well as detailed retinal imaging, fundus autofluorescence, and optical coherence tomography. Age of onset is defined as the age at which symptoms were first experienced.

Interrogation of the Genomic Region

We interrogated chromatin and genome regulation datasets to explore the epigenomic landscape of the region. Available datasets were obtained and analyzed via the UCSC genome browser (details of datasets used are provided in Supplemental Material and Methods).

Reprogramming Fibroblasts into iPSCs and Differentiation into Photoreceptor Progenitor Cells and 3D Retinal Organoids

Fibroblasts were cultured from skin biopsies of two individuals with NL-SV1, one individual with UK-SV2, and five anonymous control individuals. Cell lines were reprogrammed into induced pluripotent stem cells (iPSCs) and differentiated into photoreceptor progenitor cells (PPCs) following the previously described 60 day protocol (Supplemental Material and Methods).[16,17] 3D retinal organoids (ROs) were differentiated for UK-SV2 and controls, as previously described (Supplemental Material and Methods).[18]

Preparation of Low Input Hi-C Libraries (Low-C)

Hi-C was performed on UK-SV2 and control 3D ROs via a low input protocol (Low-C) with few modifications (Supplemental Material and Methods).[19] Two libraries per sample were sequenced for 200 million fragments in a 100 bp paired-end run on a NovaSeq 6000 (Illumina). Paired-end sequencing data was processed via Juicer[20] and the Hi-C maps were created with a bin size with 10 kb resolution. Further information about the bioinformatics pipeline is detailed in Melo et al., 2020.[21]

Expression Analysis of Genes and Enhancer RNA within the RP17 Locus

To assess expression of genes, we performed qPCR for different human tissues, including retina (Table S3 and Supplemental Material and Methods). Single-cell RNA sequencing data of human[22] and primate[23] retinal cell types were obtained and visualized via the Broad Institute Single Cell Portal (Supplemental Material and Methods).

cDNA was synthesized from total RNA extracted from PPCs, ROs, and fibroblasts. Differential expression of genes implicated in the SVs, and control housekeeping and retinal progenitor genes, was assessed by qPCR (Table S3 and Supplemental Material and Methods). We designed primers to the enhancer region containing multiple retinal transcription factor binding sites implicated in all SVs to analyze targeted enhancer RNA expression by qPCR (Table S3 and Supplemental Material and Methods).

Results

Refinement of the RP17 Locus in Two Unrelated adRP-Affected Families

The affected haplotype for a Dutch adRP-affected family (NL1) (Figure 1A) was previously mapped to a 7.18 Mb region spanning the RP17 locus on chromosome 17.[7] The RP17 locus was refined to a 5.16 Mb interval by SNP haplotyping in an extended pedigree (Figure 1D and Supplemental Information). No rare coding or splice site heterozygous variants (MAF $\leq$ 0.0001) shared between affected individuals were found through WES. Subsequently, WGS was performed, and similarly, no rare candidate coding, splice site, intronic, or intergenic heterozygous single-nucleotide variants were identified (Table S4 and Supplemental Information).

In parallel, WES and WGS were performed for affected individuals from a genetically unexplained UK adRP-affected family (UK1) (Figure 1B). This also failed to identify a rare causative variant; however, a disease-associated haplotype on chromosome 17 was identified (Figure 1E, Table S5, and Supplemental Information). Interrogation of unsolved IRD sequence data generated through the UK IRDC, UCL-Ex, NIHR-Bioresource, and Genomics England identified other adRP probands that shared the same haplotype of Chr17 SNVs and established this as a founder haplotype in eleven additional UK adRP-affected families (Figure 1C). The adRP locus was refined to a 4.4 Mb interval on Chr17q22 (Figure 1E). This genomic interval overlaps the previously described RP17 locus in families of Dutch and South African origin (Figure 1F).

A missense variant in *CA4* [c.40C>T (p.Arg14Trp); GenBank: NM_000717.4] was previously described as the cause of adRP at the RP17 locus in families of South African origin.[8] No rare coding, intronic, or upstream variants in *CA4* were identified in the Dutch and UK families.

Identification of Structural Variants within the RP17 Locus

Next, we analyzed genome and exome data for copy number variants and SVs (Supplemental Information). In family NL1, WGS revealed a 226 kb duplication within the RP17 locus: chr17: 57,291,905_57,518,137dup (NL-SV1). This SV involves two duplicated genes (*GDPD1* [MIM: 616317] and *YPEL2* [MIM: 609723]), an intragenic microRNA (*MIR4729*), and partial duplication of *SMG8* (MIM: 613175) and the long non-coding RNA *LINC01476*. The duplication creates a breakpoint junction (chr17: g.57,518,137–57,291,905) specific for the mutated allele in NL1 (Figures 2A, 2B, and S1), which was used to confirm segregation of the SV with the adRP phenotype in this family. No overlapping SVs in the RP17 locus were observed in WES of ~7,500 individuals without retinal disease generated in-house at the Department of Human Genetics, Radboudumc.

For the twelve UK RP17 founder haplotype families, WGS revealed a duplicated inversion: chr17: 57,456,098–57,468,960delins57,275,839_57,559,114inv (UK-SV2) (Figure 2B). The SV was characterized, and breakpoint junctions were validated (Figure S1 and Supplemental Information). This SV involved four coding genes (*PRR11* [MIM: 615920], *SMG8*, *GDPD1*, and *YPEL2*) and two non-coding RNA genes (*MIR4729* and *LINC01476*) (Figure 2B). UK-SV2 segregated with adRP in all families for which DNA was available for analysis. UK-SV2 was absent in WGS control genome data generated for 58,000 UK individuals (Genomics England).

Different Structural Variants within the RP17 Locus in Multiple adRP-Affected Families

These data prompted us to investigate whether SVs were present in the two original South African families (SA1 and SA2) that were linked to the RP17 locus (Figure S2A).[6,8] In addition, a Canadian adRP-affected family (CA1) was also mapped to the RP17 locus (unpublished data, Figure S2B). WGS was performed for affected individuals from these families, and inversion duplication events were identified in all samples analyzed (Figure 2C). In SA1 and SA2, an identical SV, SA-SV3, was revealed, suggesting this is a founder variant in this population. SA-SV3 was also found by breakpoint PCR in two additional families of South African origin (SA3 and SA4), confirming the founder effect (Figure S2A). In the Canadian family, a different inversion duplication event was identified, CA-SV4. SA-SV3 and CA-SV4 breakpoints were characterized and validated (Figure S1), and segregation of the SVs with the adRP phenotype was confirmed.

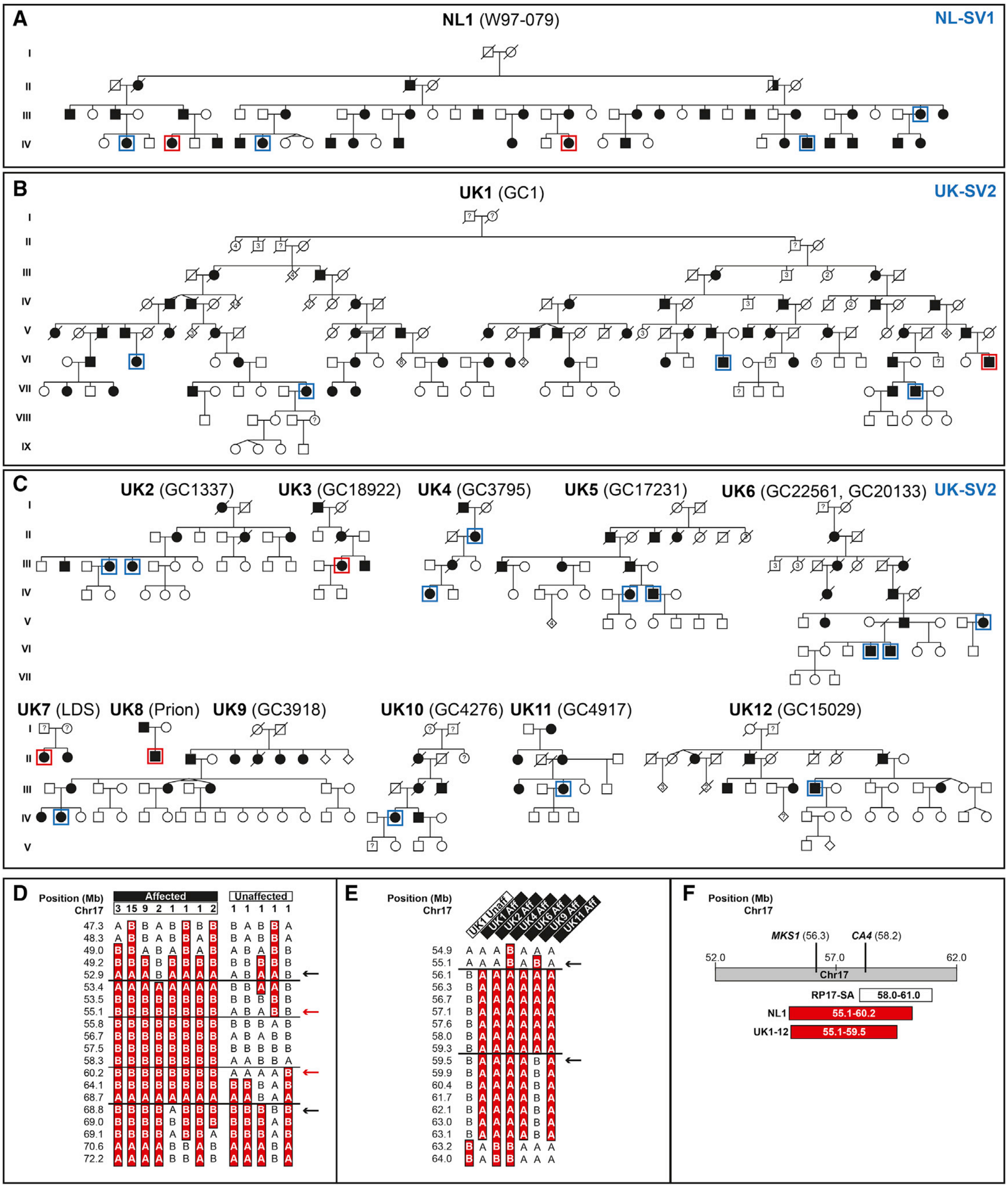

Figure 1. Mapping of the RP17 Locus in Two Unrelated Families

(A) Pedigree of Dutch NL1 family.

(B) Pedigree of UK1 family.

(C) Pedigrees of additional UK families with the founder haplotype on Chr17q. WGS or WES was performed in individuals highlighted in blue or red, respectively.

(D) SNP haplotyping results for NL1. The refined RP17 locus (rs8078110–rs9910672) is shared by all affected individuals (n = 35) and not present in unaffected individuals (n = 28, only individuals with recombination close to or refining the critical region are depicted) with a maximum LOD score of 15.0. The horizontal numbers represent the number of individuals with this haplotype.

(E) UK founder haplotype refining the RP17 locus in UK families. Representative haplotypes from several unrelated families are shown with affected (aff) individuals compared to an unaffected (unaff) individual. Black lines and arrows indicate recombination events. Shared haplotype in individuals is shaded red.

(F) Overlap of refined RP17 loci in UK, NL, and previously described SA families.[8]

Our data suggested that SVs at the RP17 locus are an important cause of adRP. Therefore, WGS and WES data for genetically unexplained adRP-affected families were analyzed for SVs within this locus. In four unrelated families of Dutch or UK origin, four additional unique complex SVs were discovered (Figures 2D and S2C). For

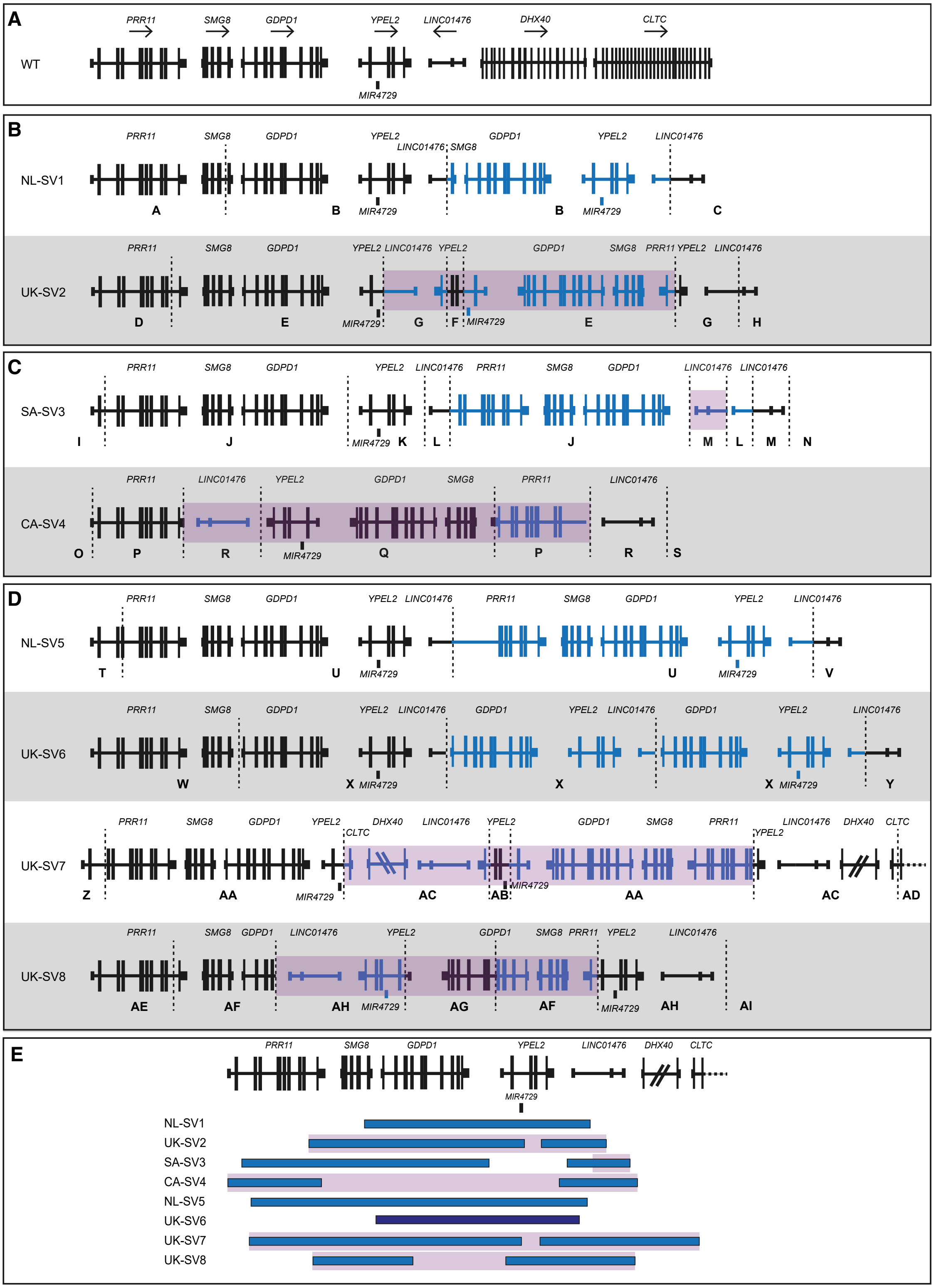

Figure 2. Overview of Structural Variants within the RP17 Locus in adRP-Affected Families
Breakpoints are indicated with dashed lines. Blue segments represent duplicated or triplicated regions, whereas inversions are highlighted in purple.
(A) Wild-type (WT) chromosomal organization.
(B) Structural variants identified in NL1 (NL-SV1) and UK founder haplotype families (UK-SV2).
(C) Structural variants identified in adRP-affected families that were previously linked to the RP17 locus; SA-SV3[8] and CA-SV4 (unpublished data).

(legend continued on next page)

individuals that had only undergone WES, we performed WGS to determine the breakpoint junctions and identify potential inversions or other SVs. In all families, breakpoints were validated, and segregation analysis was performed where possible. Triplication for UK-SV6 was confirmed by qPCR in family UK13 (Figure S3 and Supplemental Information).

Details of all SVs identified in this study are shown in Table S6, Figure 2, and Figure S4, and an overview of SV-specific breakpoint junctions is shown in Figure S1. All RP17 SVs share a common duplicated (or triplicated) region of 11.5 kb and harbor unique breakpoints disrupting the genomic region spanning *YPEL2* to *LINC01476* (chr17: 57,499,214–57,510,765) (Figure S4). We analyzed all breakpoint junction sequences to investigate the potential mechanism(s) that created RP17 SVs. No single mechanism could account for the RP17 SVs because a combination of (micro)homology-mediated repair and non-homologous end joining events were identified (Table S7 and S8, Figure S5, and Supplemental Information).

Consistent Autosomal-Dominant Retinitis Pigmentosa Phenotype for RP17-Affected Families

The SVs identified were fully penetrant in all families. Available clinical data are presented in Table S9. Twenty-four affected individuals from seventeen pedigrees were evaluated. There is significant correlation of phenotype across all genotypes with relatively mild disease, decreased visual acuity, visual field constriction, nyctalopia, and slow progression consistent with adRP. Many affected individuals have preserved central visual function and acuity until the 6th–7th decade. Foveal sparing and cystoid macular edema were a common finding in individuals with UK-SV2. On the basis of a small number of affected individuals ($n = 2$), UK-SV6 (with a triplicated SV) may be associated with an earlier age of onset and more severe phenotype (Figure S6).

Topologically Associating Domain Structure and Epigenetic Landscape of the RP17 Genomic Region

All of the RP17 SVs lead to disruption of the genomic region spanning *YPEL2* to *LINC01476* (Figure 2E). SVs that interfere with genome structure can have distinct effects on gene regulation depending on the type and extent of the SV and landscape of the genomic region.[15] TADs are separated by boundaries, regions of low chromatin interaction that insulate the regulatory activities of neighboring TADs. The transcription factor CTCF (CCTC-binding factor) typically binds in these regions where it plays a pivotal role in the maintenance of boundaries. SVs can cause loss of function by disconnecting enhancers from their target genes; however, disruption of TAD structures and boundaries can also exert a gain-of-function effect. Deletions, for example, can lead to the fusing of two previously separated TADs (TAD-fusion), inversions can result in the exchange of regulatory material between TADs (TAD-shuffling), whereas duplications can give rise to the generation of novel domains, so-called neo-TADs.[12,13] In each case, SVs result in the generation of ectopic contacts of enhancers with the promoters of novel target genes resulting in aberrant gene activation. The human limb malformations caused by SVs that alter the CTCF-associated boundary of the WNT6/IHH/EPHA4/PAX3 locus are a prominent example. The SVs result in ectopic interactions between *EPHA4* (MIM: 602188) limb enhancers and the neighboring developmental genes that are normally insulated, driving ectopic expression in the limb.[14] Similarly, the deletion of a CTCF site located between the *Xist* (MIM: 314670) and *Tsix* (MIM: 300181) TADs on the X chromosome resulted in a novel domain by fusion of the adjacent TADs (fused-TAD).[24] As a consequence, previously insulated enhancers activated genes in the adjacent TAD, leading to the dysregulation of these genes.

Hi-C data were not available for human retina, and therefore, we generated Hi-C maps of control human 3D ROs to obtain maps of the chromatin organization of our region of interest. Hi-C revealed a structured domain containing *YPEL2* (*YPEL2* TAD) flanked by less structured neighboring domains (Figure 3A). CTCF binding is present on both boundaries (Figure 3B) supporting the TAD structure at this locus. CTCF ChIA-PET data highlighted interactions between the CTCF binding sites at the 5′ and the 3′ boundary of the *YPEL2* TAD (Figure S7B).

Assay for transposase-accessible chromatin using seqencing (ATAC-seq) data from human retina show that the chromatin in the *YPEL2* TAD is accessible, and H3K27Ac ChIP-seq data revealed that there are several active enhancers located within the *YPEL2* TAD that are expected to drive *YPEL2* expression in the retina (Figure 3B).[25] Importantly, the *YPEL2* TAD harbors two regions of active enhancers with binding sites for transcription factor (TFs) known to be required for photoreceptor function, including NRL, CRX, and OTX2 (Figure 3B). NRL is a TF that is preferentially expressed in rod photoreceptors. These TF binding sites correlated with H3K27Ac and ATAC-seq peaks in retina. The published GeneHancer data-set shows that these regulatory elements have interactions with the *YPEL2* promoter (Figure S7C).[26] Collectively, these analyses revealed that *YPEL2* is located within an active compartment that contains retinal-specific enhancers (Figure 3C).

(D) Structural variants found in a cohort of unsolved adRP-affected families; NL-SV5, UK-SV6, UK-SV7, and UK-SV8. Letters A–AI depict the genomic intervals for each SV used to analyze and annotate SV breakpoints.
(E) Overview of all SV breakpoints identified in the RP17 locus. An overlapping genomic region that is duplicated or triplicated in all SVs was identified (chr17: 57,499,214–57,510,765) and is highlighted by a light blue vertical bar. The size of *DHX40* is reduced, and *CLTC* is partially shown for the purpose of this figure.

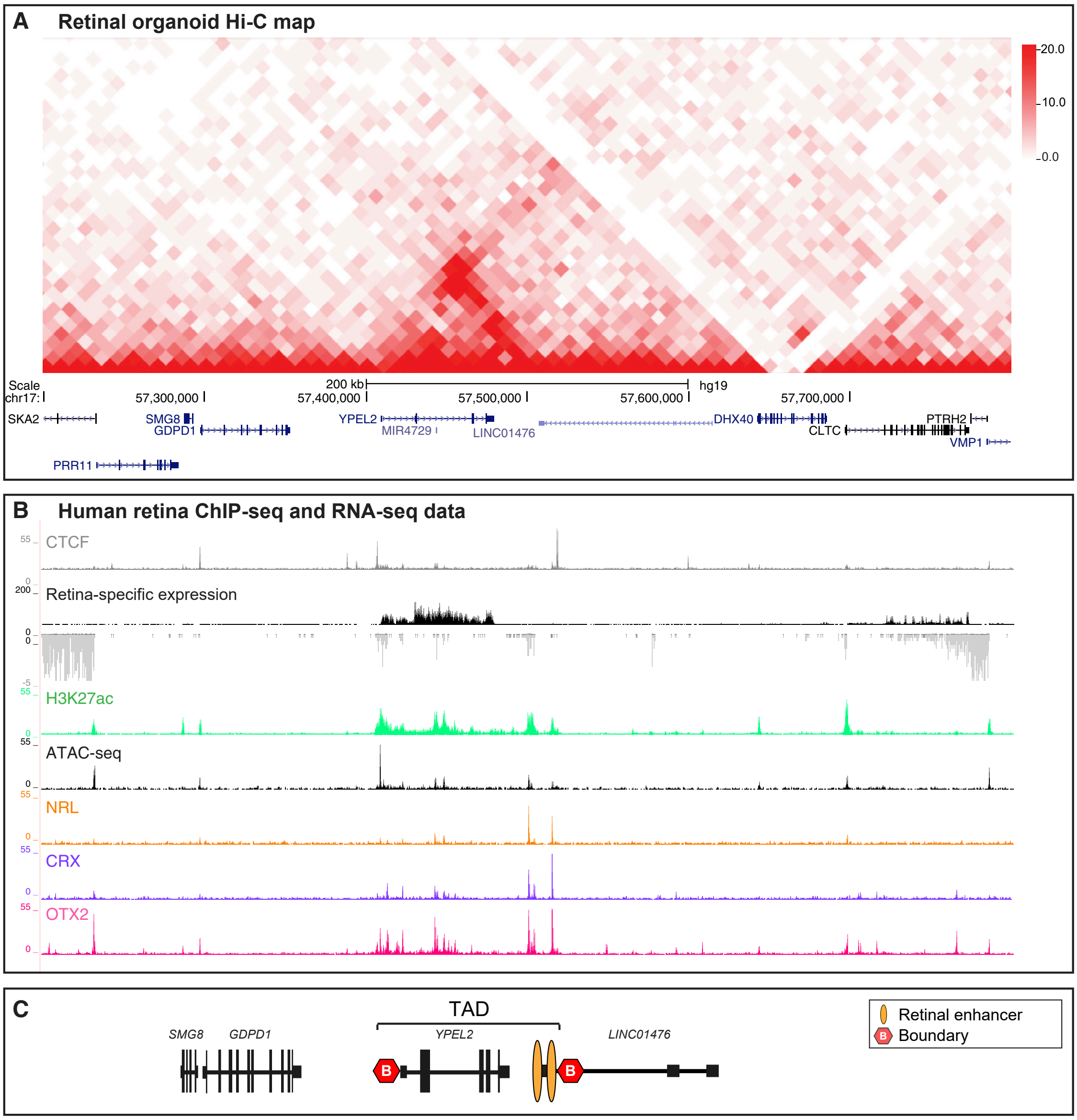

Figure 3. ***YPEL2* is Located within a Structured Active Compartment that Contains Retinal-Specific Enhancers**
(A) The TAD landscape of the genomic region disrupted by the RP17 SVs. Hi-C map of control retinal organoids revealed a structured domain containing *YPEL2*.
(B) *YPEL2* TAD boundaries correspond with CTCF sites identified in human retina. Analysis of RNA-seq and assay for transposase-accessible chromatin using seqencing (ATAC-seq) data across the *YPEL2* region shows *YPEL2* retinal expression and an accessible chromatin configuration. Analysis of H3K27Ac ChIP-seq data in the same region revealed several active enhancers located within the *YPEL2* TAD, which are enriched for retinal transcription factor binding sites, including NRL, CRX, and OTX2.[25] These enhancers were located 5′ of the CTCF boundary site within *LINC01476*.
(C) Schematic representation of the *YPEL2* TAD structure.

Expression of *YPEL2* and *GDPD1*

Expression of *YPEL2* and *GDPD1* was assessed by qPCR in multiple healthy human tissues, including retina (Figure S8). *YPEL2* is ubiquitously expressed in the tissues studied, including retina, with highest relative expression in brain. Single-cell retina RNA-seq datasets revealed *YPEL2* is expressed at higher levels in rod photoreceptor cells, which is the primary cell type affected in retinitis pigmentosa, compared to cone photoreceptors (Figure S8).[23] *GDPD1* is detected at low expression in all tissue types but has higher expression in testis and the brain. These data support the hypothesis that *YPEL2* expression is regulated by retinal enhancers within the *YPEL2* TAD.

RP17 SVs Create New Topologically Associating Domains and Ectopic Enhancer-Gene Interactions

Using the wild-type retinal organoid Hi-C map, we modeled the TAD boundaries, CTCF site orientation, and retinal TF binding site positions for each unique RP17 SV

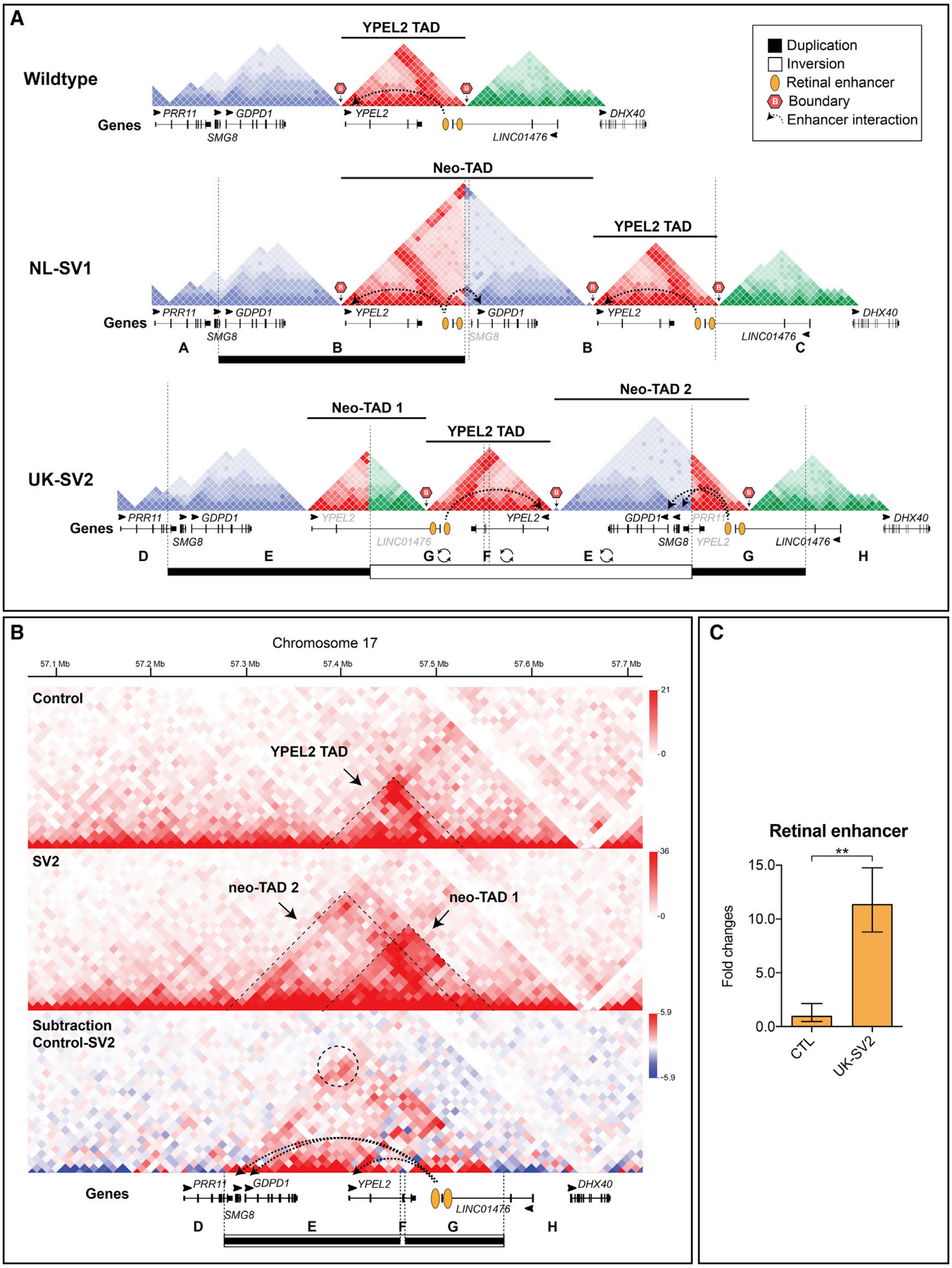

Figure 4. RP17 SVs Create Novel Domains (neo-TADs) and Hyper-activation of Retinal Enhancers
(A) Schematic modeling of the genome architecture spanning the RP17 region using Hi-C maps. The wild-type Hi-C map derived from neuronal tissue shows a TAD with CTCF boundaries containing *YPEL2* and retinal enhancers, flanked by unstructured domains. TAD models of NL-SV1 and UK-SV2 (dotted vertical lines represent SV breakpoints) predict the formation of neo-TADs and ectopic interactions of the retinal enhancer with *GDPD1*.
(B) Hi-C performed on retinal organoids (ROs) derived from control (top) and RP17 UK-SV2 individuals (bottom) (10 kb resolution; raw count map). The chromatin organization in control ROs shows the *YPEL2* TAD (indicated by dashed lines). Two novel domains (neo-TAD 1 and 2) are visible in the UK-SV2 ROs, and neo-TAD 2 allows ectopic retinal enhancer contacts to *GDPD1* and *SMG8*. The dashed circle indicates the strong chromatin contact between retinal enhancers and the *GDPD1* promoter.
(C) qPCR revealed significantly upregulated retinal enhancer RNA expression in UK-SV2 ROs compared to controls (n = 3, mean ± standard error of the mean, **p ≤ 0.01).

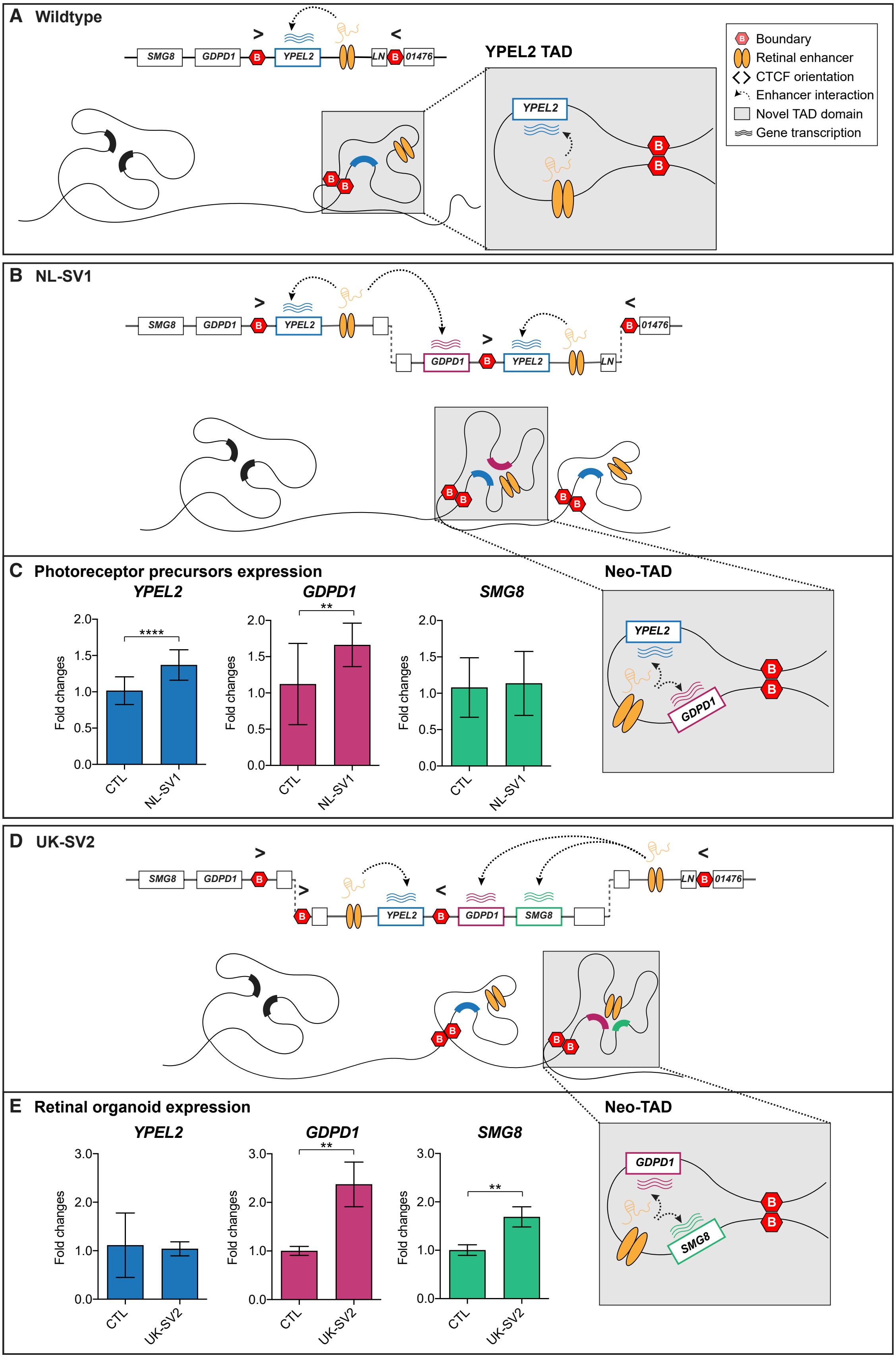

(legend on next page)

(Figures 4A and S9). In NL-SV1, the duplication contains part of the *YPEL2* TAD, the boundary to the neighboring region and *GDPD1*. This results in the creation of a neo-TAD that now contains the previously separated *YPEL2* enhancers and *GDPD1* in one domain. To directly investigate the effect of the SVs in retinal cells, we reprogrammed dermal fibroblasts from UK-SV2 to iPSC and differentiated to 3D ROs, thus creating an *in vitro* model (Supplemental Information). In this case, the duplicated regions are also inverted. Hi-C of RP17 ROs (UK-SV2) revealed the creation of two neo-TADs compared to control ROs (Figure 4B). The rearrangement of CTCF sites caused by the SV creates boundaries for two novel domains (neo-TAD 1 and 2) where neo-TAD 2 contains a duplicated copy of *GDPD1* and *SMG8* and the retinal enhancers, confirming the modeling for this SV (Figure 4A). Furthermore, on the basis of our predictions, neo-TADs are created in each of the RP17 cases and *GDPD1* is predicted to gain ectopic access to the retinal-specific enhancers (Figures 4A and S9). Therefore, the potential convergent mechanism for retinal degeneration is transcriptional activation and expression of *GDPD1* through juxtaposition of retinal TF binding sites within active compartments bounded by CTCF sites. This model would also fit with a dominant gain-of-function mechanism of disease.

Next, we assessed retinal enhancer expression in control and UK-SV2 ROs by enhancer RNA qPCR (Supplemental Information). A significant increase of the retinal enhancer was detected in RP17 ROs (Figure 4C), demonstrating that this transcriptionally active retinal enhancer in the neo-TAD could drive retinal expression of *GDPD1*.

Differential Expression of *GDPD1* in RP17 iPSC-Derived Photoreceptor Precursors and 3D Retinal Organoids

Our experimental data and modeling predict *GDPD1* enters a neo-TAD with retinal enhancers in all RP17 SVs. An extra copy of *YPEL2* enters the neo-TAD of NL-SV1, and *SMG8* enters this domain in UK-SV2 (Figures 5 and S9).

To experimentally validate the consequence of RP17 SVs in genomic and cellular context, we performed qPCR to assess differential expression in PPCs (NL-SV1) and ROs (UK-SV2). The expression of *GDPD1*, *YPEL2*, and *SMG8* was compared to controls (Supplemental Information).

In both experimental models, the expression of *GDPD1* was significantly increased compared to controls. *YPEL2* was increased in NL-SV1 only (Figure 5C), whereas *SMG8* was increased in UK-SV2 (Figure 5E), which correlates with our TAD modeling and Hi-C experimental data for UK-SV2 ROs (Figured 5B, 5D, and S9). To further explore the tissue-specific effect of this transcriptional upregulation, we performed the same qPCR assays on fibroblasts of the same individuals. None of these genes had increased expression in affected individuals compared to controls (data not shown).

Discussion

Previous genetic studies of adRP-affected families mapping to the RP17 locus have implicated missense variants in *CA4* as the cause of disease or have been unable to confirm pathogenicity.[7,8] Here, we describe the discovery of SVs as the cause of adRP at the RP17 locus in a large number of families, suggesting this is a previously unrecognized major locus for adRP. Our results show how complex rearrangements can result in the disruption of 3D genome architecture, the re-wiring of enhancer-promoter interactions, and consequent gene misexpression.

Following the identification of SVs in NL1 and UK1 via short-read WGS, our search for similar complex SVs in the RP17 genomic interval of genetically unexplained adRP-affected families identified six other complex SVs that segregated with disease. SVs are a major source of normal variation in the human genome and are often benign;[27,28] however, none of the RP17 SVs are found in the population database gnomAD[29] or the Database of Genomic Variants (DGV).[30] Although overlapping canonical SVs (deletions and duplications) have been identified, they do not have breakpoints within the *YPEL2-LINC01476* region, as observed for all RP17 SVs reported in this study. This is in line with observations that different SVs can have different consequences depending on the characteristics of specific SVs in local 3D chromatin and epigenetic context.[31,32]

Base level resolution of breakpoint junctions and interrogation of the DNA sequence signatures revealed the mechanisms of the chromosomal rearrangements. Repetitive elements are key factors in facilitating unequal crossover of genomic segments or providing microhomology that induces fork stalling and subsequent template switching.[33,34] Consistent with this model, repetitive elements were present in the flanking sequences of breakpoint

Figure 5. Convergent Mechanism of Ectopic Retinal Enhancer-*GDPD1* Interaction Caused by RP17 SVs
(A) In wild-type genomic context, *YPEL2* expression in retina is driven by retinal enhancers in a TAD with CTCF boundaries. Neighboring genes are insulated from retinal enhancer activation.
(B) The NL-SV1 duplication creates a neo-TAD with a full-length copy of *YPEL2*, *GDPD1*, and the retinal enhancers. This enables retinal-specific enhancers to ectopically interact with *GDPD1*, which drives its misexpression.
(C) qPCR analysis of photoreceptor precursor cells (PPCs) revealed a significant upregulation of *GDPD1* in NL-SV1 PPCs compared to controls.
(D) The UK-SV2 duplication and inversion creates a neo-TAD with a full-length copy of *GDPD1* and *SMG8* and the retinal enhancers bounded by CTFC sites.
(E) qPCR analysis ROs revealed a significant upregulation of *GDPD1* expression in UK-SV2 ROs compared to controls ($n = 3$ independent ROs, mean $\pm$ standard error of the mean, $^{**}p \leq 0.01$, $^{****}p \leq 0.0001$).

junctions. In addition, microhomology, larger stretches of homology, and small insertions-deletions were found at all breakpoints.[34–36] Therefore, repetitive elements may explain why the RP17 locus is prone to such structural variation, which is supported by the presence of breakpoint "hotspots," as seen in *LINC01476* intron 2 and *YPEL2* intron 4; some breakpoints only differ by a small number of base pairs (e.g., for UK-SV2 and UK-SV7).

None of the genes implicated in the RP17 SVs have been previously associated with retinal disease. *YPEL2* is expressed in retina, and single-cell RNA sequencing of human and primate retina revealed expression in photoreceptors; the highest expression was in rod photoreceptors.[22,23] Although the function of YPEL2 in the retina is unknown, we show that retinal expression is controlled by a number of retinal TF binding sites, including NRL, which is predominantly expressed in rod photoreceptors. Furthermore, Hi-C data show that *YPEL2* and the retinal enhancer binding sites are insulated from the surrounding region in a structured *YPEL2* TAD in control ROs and other tissues.

Hi-C analyses of UK-SV2 ROs revealed the generation of neo-TADs, and altered structure and repositioning of the boundaries enabled *GDPD1* promoter-retinal enhancer contacts and consequent *GDPD1* misexpression in the retina. The molecular disease mechanism in these cases is similar to the reported duplications at the SOX9/KCNJ2 locus.[13] As described for the rearrangements reported here, the duplications at the *SOX9* (MIM: 608160) locus also encompass a regulatory domain (of *SOX9*), a boundary (between the *SOX9* and the *KCNJ2* [MIM: 600681] TADs), and the neighboring gene (*KCNJ2*). This results in the formation of a neo-TAD containing the *SOX9* regulatory elements and the new target gene (*KCNJ2*) that are now free to interact. In the *SOX9* case, this leads to misexpression of *KCNJ2* in a *SOX9* pattern and consecutive limb malformation, whereas in the RP-affected individuals, the interaction of *GDPD1* with *YPEL2* enhancers leads to misexpression in the retina. However, in some of the RP-affected individuals, such as UK-SV2, the situation is more complex because the duplications are inverted. Inversions can lead to the exchange of regulatory material from one end of the breakpoint to the other (also called TAD-shuffling).[12] In UK-SV2, the duplication creates two neo-TADs, but the content is reorganized by the inversion. Again, *GDPD1* and retinal enhancers are brought together in one new TAD. Thus, the pathogenetic principle remains the same because all the RP17 SVs are predicted to create new TADs allowing access of the retinal enhancers to *GDPD1*. This suggests that increased expression of *GDPD1* in photoreceptors is the convergent mechanism of disease. Consistent with this hypothesis, PPCs from NL-SV1 and ROs from UK-SV2 showed significant increased expression of *GDPD1* in RP17-affected families with different SVs compared to controls. In UK-SV2 ROs, an increased expression of *SMG8*, which is also introduced into the active neo-TAD of UK-SV2, was observed. Conversely, *YPEL2* shows upregulation in NL-SV1, which is in line with the complete duplication of *YPEL2* in NL-SV1. Importantly, qPCR provided evidence for the increased expression of the retinal enhancer in UK-SV2 ROs and TF binding sites for NRL, which is preferentially expressed in rod photoreceptors, the primary cell type affected in RP.

Although increased expression of *SMG8*, *YPEL2*, or the retinal enhancer cannot be excluded from contributing to the phenotype in individual families, these experimental data support the hypothesis of a convergent mechanism of *GDPD1* entry into the active neo-TAD with retinal enhancers for all eight complex RP17 SVs. This is further supported by the observation that the two affected individuals in family UK13, who had an earlier age of onset and more severe phenotype compared to all other families, have a triplication (UK-SV6) where two copies of *GDPD1* are predicted to enter the active neo-TAD.

Our data implicate increased retinal expression of *GDPD1* as a dominant gain-of-function mechanism leading to adRP. *GDPD1* encodes a glycerophosphodiesterase, which can hydrolyze lysophosphatidylcholine (lyso-PC) to lysophosphatidic acid (LPA)[37] with lysophospholipase D (lysoPLD) activity on various lysophospholipids.[38] *GPDP1* is detected at low expression in the healthy retina, and therefore, increased expression of *GDPD1* could lead to dysregulation of lipid metabolism, which is known to be critical for photoreceptor function, although the exact mechanisms of photoreceptor cell death are not known.[39–41] Disruption of lipid metabolism leading to adRP, combined with the adult age of onset, opens avenues for therapeutic intervention to preserve vision by restoring lipid homeostasis.

Data and Code Availability

Extensive data are presented in the Supplemental Information to enable others to perform similar studies and replicate our findings. WES and WGS data have not been deposited in a public repository because of consent and ethical considerations. Hi-C data are available on request.

Supplemental Data

Supplemental Data can be found online at https://doi.org/10.1016/j.ajhg.2020.09.002.

Acknowledgments

We thank the affected individuals and their families for participating in this study and our funding bodies. We thank Saskia D. van de Velde-Visser for cell culture work; Marijke N. Zonneveld-Vrieling, Mubeen Khan, Rolph Pfundt, and M. Imran Khan for experimental contributions; Alex Hoischen for expert contribution; Galuh Dyah Nur Astuti for bioinformatics support; Edward Bloch for taking skin biopsies; and Karin W. Littink, Suzanne IJzer, Irma Lopez, Anneke I. den Hollander, and Samantha Malka for their effort in sample collection. This research was supported by Retina UK; Fight for Sight; Moorfields Eye Charity; the National

Institute for Health Research Biomedical Research Centre at Moorfields Eye Hospital National Health Service Foundation Trust; UCL Institute of Ophthalmology; GOSH; Cambridge; The Wellcome Trust (205041/Z/16/Z grant 538842); a DCN Radboudumc grant (to F.P.M.C. and H.K.); Foundation Fighting Blindness (PPA-0517-0717-RAD to S.R. and F.P.M.C.); ANVTVVB, Oogfonds, and LSBS; Rotterdamse Stichting Blindenbelangen, Stichting Blindenhulp, Stichting tot Verbetering van het Lot der Blinden, and Stichting Blinden-Penning (to S.R. and F.P.M.C.); Foundation Fighting Blindness, Reseau de Vision and MCH Foundation (to R.K.K.); Swiss National Science Foundation (176097 to C.R.); the MRC (UK to S.M.); and Retina South Africa and the South African Medical Research Council (to L.R.). The Steunfonds RP17 financially supported experiments for this project (to S.R. and F.P.M.C.). This research accessed 100,000 Genomes Project data, managed by Genomics England Limited (a wholly owned company of the Department of Health). The 100,000 Genomes Project is funded by the National Institute for Health Research and NHS England. The Wellcome Trust, Cancer Research UK, and the Medical Research Council have also funded research infrastructure.

Declaration of Interests

The authors declare no competing interests.

Received: July 25, 2020
Accepted: September 2, 2020
Published: October 5, 2020

Web Resources

GeneReviews, Fahim, A.T., Daiger, S.P., and Weleber, R.G. (2017). Nonsyndromic Retinitis Pigmentosa Overview, https://www.ncbi.nlm.nih.gov/books/NBK1417/

RetNet, https://sph.uth.edu/RetNet

References

1. Haer-Wigman, L., van Zelst-Stams, W.A., Pfundt, R., van den Born, L.I., Klaver, C.C., Verheij, J.B., Hoyng, C.B., Breuning, M.H., Boon, C.J., Kievit, A.J., et al. (2017). Diagnostic exome sequencing in 266 Dutch patients with visual impairment. Eur. J. Hum. Genet. *25*, 591–599.
2. Verbakel, S.K., van Huet, R.A.C., Boon, C.J.F., den Hollander, A.I., Collin, R.W.J., Klaver, C.C.W., Hoyng, C.B., Roepman, R., and Klevering, B.J. (2018). Non-syndromic retinitis pigmentosa. Prog. Retin. Eye Res. *66*, 157–186.
3. Hamel, C. (2006). Retinitis pigmentosa. Orphanet J. Rare Dis. *1*, 40.
4. Sullivan, L.S., Bowne, S.J., Reeves, M.J., Blain, D., Goetz, K., Ndifor, V., Vitez, S., Wang, X., Tumminia, S.J., and Daiger, S.P. (2013). Prevalence of mutations in eyeGENE probands with a diagnosis of autosomal dominant retinitis pigmentosa. Invest. Ophthalmol. Vis. Sci. *54*, 6255–6261.
5. Daiger, S.P., Bowne, S.J., and Sullivan, L.S. (2014). Genes and Mutations Causing Autosomal Dominant Retinitis Pigmentosa. Cold Spring Harb. Perspect. Med. *5*, a017129.
6. Bardien, S., Ebenezer, N., Greenberg, J., Inglehearn, C.F., Bartmann, L., Goliath, R., Beighton, P., Ramesar, R., and Bhattacharya, S.S. (1995). An eighth locus for autosomal dominant retinitis pigmentosa is linked to chromosome 17q. Hum. Mol. Genet. *4*, 1459–1462.
7. den Hollander, A.I., van der Velde-Visser, S.D., Pinckers, A.J.L.G., Hoyng, C.B., Brunner, H.G., and Cremers, F.P.M. (1999). Refined mapping of the gene for autosomal dominant retinitis pigmentosa (RP17) on chromosome 17q22. Hum. Genet. *104*, 73–76.
8. Rebello, G., Ramesar, R., Vorster, A., Roberts, L., Ehrenreich, L., Oppon, E., Gama, D., Bardien, S., Greenberg, J., Bonapace, G., et al. (2004). Apoptosis-inducing signal sequence mutation in carbonic anhydrase IV identified in patients with the RP17 form of retinitis pigmentosa. Proc. Natl. Acad. Sci. USA *101*, 6617–6622.
9. Köhn, L., Burstedt, M.S.I., Jonsson, F., Kadzhaev, K., Haamer, E., Sandgren, O., and Golovleva, I. (2008). Carrier of R14W in carbonic anhydrase IV presents Bothnia dystrophy phenotype caused by two allelic mutations in RLBP1. Invest. Ophthalmol. Vis. Sci. *49*, 3172–3177.
10. Golovleva, I., Köhn, L., Burstedt, M., Daiger, S., and Sandgren, O. (2010). Mutation spectra in autosomal dominant and recessive retinitis pigmentosa in northern Sweden. Adv. Exp. Med. Biol. *664*, 255–262.
11. Rao, S.S.P., Huntley, M.H., Durand, N.C., Stamenova, E.K., Bochkov, I.D., Robinson, J.T., Sanborn, A.L., Machol, I., Omer, A.D., Lander, E.S., and Aiden, E.L. (2014). A 3D map of the human genome at kilobase resolution reveals principles of chromatin looping. Cell *159*, 1665–1680.
12. Spielmann, M., Lupiáñez, D.G., and Mundlos, S. (2018). Structural variation in the 3D genome. Nat. Rev. Genet. *19*, 453–467.
13. Franke, M., Ibrahim, D.M., Andrey, G., Schwarzer, W., Heinrich, V., Schöpflin, R., Kraft, K., Kempfer, R., Jerković, I., Chan, W.-L., et al. (2016). Formation of new chromatin domains determines pathogenicity of genomic duplications. Nature *538*, 265–269.
14. Lupiáñez, D.G., Kraft, K., Heinrich, V., Krawitz, P., Brancati, F., Klopocki, E., Horn, D., Kayserili, H., Opitz, J.M., Laxova, R., et al. (2015). Disruptions of topological chromatin domains cause pathogenic rewiring of gene-enhancer interactions. Cell *161*, 1012–1025.
15. Ibrahim, D.M., and Mundlos, S. (2020). Three-dimensional chromatin in disease: What holds us together and what drives us apart? Curr. Opin. Cell Biol. *64*, 1–9.
16. Albert, S., Garanto, A., Sangermano, R., Khan, M., Bax, N.M., Hoyng, C.B., Zernant, J., Lee, W., Allikmets, R., Collin, R.W.J., and Cremers, F.P.M. (2018). Identification and Rescue of Splice Defects Caused by Two Neighboring Deep-Intronic ABCA4 Mutations Underlying Stargardt Disease. Am. J. Hum. Genet. *102*, 517–527.
17. Sangermano, R., Bax, N.M., Bauwens, M., van den Born, L.I., De Baere, E., Garanto, A., et al. (2016). Photoreceptor Progenitor mRNA Analysis Reveals Exon Skipping Resulting from the ABCA4 c.5461-10T>C Mutation in Stargardt Disease. Ophthalmology *123*, 1375–1385.
18. Schwarz, N., Lane, A., Jovanovic, K., Parfitt, D.A., Aguila, M., Thompson, C.L., da Cruz, L., Coffey, P.J., Chapple, J.P., Hardcastle, A.J., and Cheetham, M.E. (2017). Arl3 and RP2 regulate the trafficking of ciliary tip kinesins. Hum. Mol. Genet. *26*, 2480–2492.
19. Díaz, N., Kruse, K., Erdmann, T., Staiger, A.M., Ott, G., Lenz, G., and Vaquerizas, J.M. (2018). Chromatin conformation analysis of primary patient tissue using a low input Hi-C method. Nat. Commun. *9*, 4938.

20. Durand, N.C., Shamim, M.S., Machol, I., Rao, S.S.P., Huntley, M.H., Lander, E.S., and Aiden, E.L. (2016). Juicer Provides a One-Click System for Analyzing Loop-Resolution Hi-C Experiments. Cell Syst. *3*, 95–98.
21. Melo, U.S., Schöpflin, R., Acuna-Hidalgo, R., Mensah, M.A., Fischer-Zirnsak, B., Holtgrewe, M., Klever, M.K., Türkmen, S., Heinrich, V., Pluym, I.D., et al. (2020). Hi-C identifies complex genomic rearrangements and TAD-shuffling in developmental diseases. Am. J. Hum. Genet. *106*, 872–884.
22. Lukowski, S.W., Lo, C.Y., Sharov, A.A., Nguyen, Q., Fang, L., Hung, S.S., Zhu, L., Zhang, T., Grünert, U., Nguyen, T., et al. (2019). A single-cell transcriptome atlas of the adult human retina. EMBO J. *38*, e100811.
23. Peng, Y.-R., Shekhar, K., Yan, W., Herrmann, D., Sappington, A., Bryman, G.S., van Zyl, T., Do, M.T.H., Regev, A., and Sanes, J.R. (2019). Molecular Classification and Comparative Taxonomics of Foveal and Peripheral Cells in Primate Retina. Cell *176*, 1222–1237.e22.
24. Dixon, J.R., Selvaraj, S., Yue, F., Kim, A., Li, Y., Shen, Y., Hu, M., Liu, J.S., and Ren, B. (2012). Topological domains in mammalian genomes identified by analysis of chromatin interactions. Nature *485*, 376–380.
25. Cherry, T.J., Yang, M.G., Harmin, D.A., Tao, P., Timms, A.E., Bauwens, M., Allikmets, R., Jones, E.M., Chen, R., De Baere, E., et al. (2020). Mapping the cis-regulatory architecture of the human retina reveals noncoding genetic variation in disease. Proceedings of the National Academy of Sciences *117*, 9001–9012.
26. Fishilevich, S., Nudel, R., Rappaport, N., Hadar, R., Plaschkes, I., Iny Stein, T., Rosen, N., Kohn, A., Twik, M., Safran, M., et al. (2017). GeneHancer: genome-wide integration of enhancers and target genes in GeneCards. Database (Oxford) *2017*, bax028.
27. Conrad, D.F., Bird, C., Blackburne, B., Lindsay, S., Mamanova, L., Lee, C., Turner, D.J., and Hurles, M.E. (2010). Mutation spectrum revealed by breakpoint sequencing of human germline CNVs. Nat. Genet. *42*, 385–391.
28. Sudmant, P.H., Rausch, T., Gardner, E.J., Handsaker, R.E., Abyzov, A., Huddleston, J., Zhang, Y., Ye, K., Jun, G., Fritz, M.H.-Y., et al.; 1000 Genomes Project Consortium (2015). An integrated map of structural variation in 2,504 human genomes. Nature *526*, 75–81.
29. Karczewski, K.J., Francioli, L.C., Tiao, G., Cummings, B.B., Alföldi, J., Wang, Q., et al. (2020). Variation across 141,456 human exomes and genomes reveals the spectrum of loss-of-function intolerance across human protein-coding genes. Nature *581*, 434–443.
30. MacDonald, J.R., Ziman, R., Yuen, R.K.C., Feuk, L., and Scherer, S.W. (2014). The Database of Genomic Variants: a curated collection of structural variation in the human genome. Nucleic Acids Res. *42*, D986–D992.
31. Lohan, S., Spielmann, M., Doelken, S.C., Flöttmann, R., Muhammad, F., Baig, S.M., Wajid, M., Hülsemann, W., Habenicht, R., Kjaer, K.W., et al. (2014). Microduplications encompassing the Sonic hedgehog limb enhancer ZRS are associated with Haas-type polysyndactyly and Laurin-Sandrow syndrome. Clin. Genet. *86*, 318–325.
32. Ngcungcu, T., Oti, M., Sitek, J.C., Haukanes, B.I., Linghu, B., Bruccoleri, R., Stokowy, T., Oakeley, E.J., Yang, F., Zhu, J., et al. (2017). Duplicated Enhancer Region Increases Expression of CTSB and Segregates with Keratolytic Winter Erythema in South African and Norwegian Families. Am. J. Hum. Genet. *100*, 737–750.
33. Gu, S., Yuan, B., Campbell, I.M., Beck, C.R., Carvalho, C.M.B., Nagamani, S.C.S., Erez, A., Patel, A., Bacino, C.A., Shaw, C.A., et al. (2015). Alu-mediated diverse and complex pathogenic copy-number variants within human chromosome 17 at p13.3. Hum. Mol. Genet. *24*, 4061–4077.
34. Zhang, F., Khajavi, M., Connolly, A.M., Towne, C.F., Batish, S.D., and Lupski, J.R. (2009). The DNA replication FoSTeS/MMBIR mechanism can generate genomic, genic and exonic complex rearrangements in humans. Nat. Genet. *41*, 849–853.
35. Lupski, J.R. (1998). Genomic disorders: structural features of the genome can lead to DNA rearrangements and human disease traits. Trends Genet. *14*, 417–422.
36. Lieber, M.R. (2010). The mechanism of double-strand DNA break repair by the nonhomologous DNA end-joining pathway. Annu. Rev. Biochem. *79*, 181–211.
37. Ohshima, N., Kudo, T., Yamashita, Y., Mariggiò, S., Araki, M., Honda, A., Nagano, T., Isaji, C., Kato, N., Corda, D., et al. (2015). New members of the mammalian glycerophosphodiester phosphodiesterase family: GDE4 and GDE7 produce lysophosphatidic acid by lysophospholipase D activity. J. Biol. Chem. *290*, 4260–4271.
38. Tsuboi, K., Okamoto, Y., Rahman, I.A.S., Uyama, T., Inoue, T., Tokumura, A., and Ueda, N. (2015). Glycerophosphodiesterase GDE4 as a novel lysophospholipase D: a possible involvement in bioactive N-acylethanolamine biosynthesis. Biochim. Biophys. Acta *1851*, 537–548.
39. Friedman, J.S., Chang, B., Krauth, D.S., Lopez, I., Waseem, N.H., Hurd, R.E., Feathers, K.L., Branham, K.E., Shaw, M., Thomas, G.E., et al. (2010). Loss of lysophosphatidylcholine acyltransferase 1 leads to photoreceptor degeneration in rd11 mice. Proc. Natl. Acad. Sci. USA *107*, 15523–15528.
40. Kmoch, S., Majewski, J., Ramamurthy, V., Cao, S., Fahiminiya, S., Ren, H., MacDonald, I.M., Lopez, I., Sun, V., Keser, V., et al.; Care4Rare Canada (2015). Mutations in PNPLA6 are linked to photoreceptor degeneration and various forms of childhood blindness. Nat. Commun. *6*, 5614.
41. Lidgerwood, G.E., Morris, A.J., Conquest, A., Daniszewski, M., Rooney, L.A., Lim, S.Y., Hernández, D., Liang, H.H., Allen, P., Connell, P.P., et al. (2018). Role of lysophosphatidic acid in the retinal pigment epithelium and photoreceptors. Biochim. Biophys. Acta Mol. Cell Biol. Lipids *1863*, 750–761.

The American Journal of Human Genetics, Volume *107*

Supplemental Data

Structural Variants Create New Topological-Associated Domains and Ectopic Retinal Enhancer-Gene Contact in Dominant Retinitis Pigmentosa

Suzanne E. de Bruijn, Alessia Fiorentino, Daniele Ottaviani, Stephanie Fanucchi, Uirá S. Melo, Julio C. Corral-Serrano, Timo Mulders, Michalis Georgiou, Carlo Rivolta, Nikolas Pontikos, Gavin Arno, Lisa Roberts, Jacquie Greenberg, Silvia Albert, Christian Gilissen, Marco Aben, George Rebello, Simon Mead, F. Lucy Raymond, Jordi Corominas, Claire E.L. Smith, Hannie Kremer, Susan Downes, Graeme C. Black, Andrew R. Webster, Chris F. Inglehearn, L. Ingeborgh van den Born, Robert K. Koenekoop, Michel Michaelides, Raj S. Ramesar, Carel B. Hoyng, Stefan Mundlos, Musa M. Mhlanga, Frans P.M. Cremers, Michael E. Cheetham, Susanne Roosing, and Alison J. Hardcastle

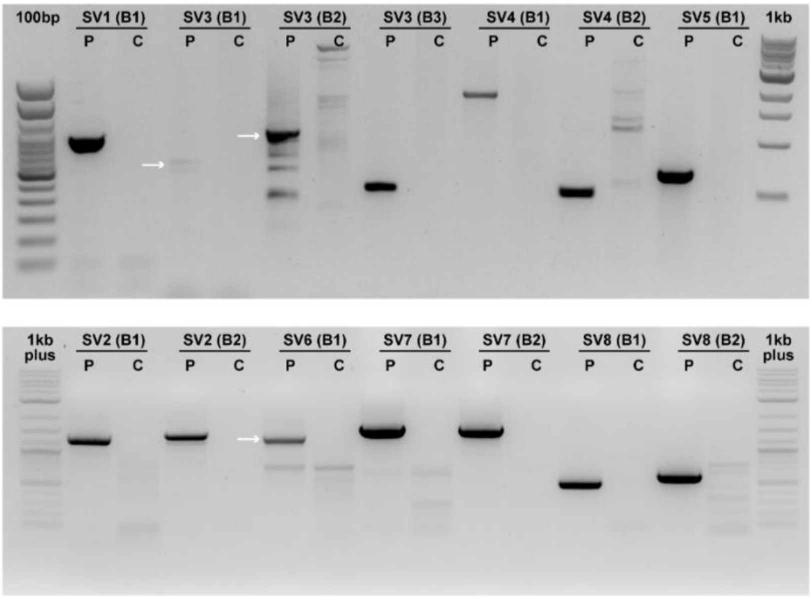

Figure S1: Agarose gels of of allele-specific breakpoint junction PCR amplications. PCR amplification of allele-specific breakpoints was performed, and gel electrophoresis was used for visualization. For each structural variant, allele-specific breakpoints (B) could be amplified in affected individual DNA (P) but not in DNA obtained from anonymous controls (C). Breakpoint junction nomenclature corresponds to those illustrated in Figure 2. Primer sequences used for PCR amplifcation are listed in Table S2.

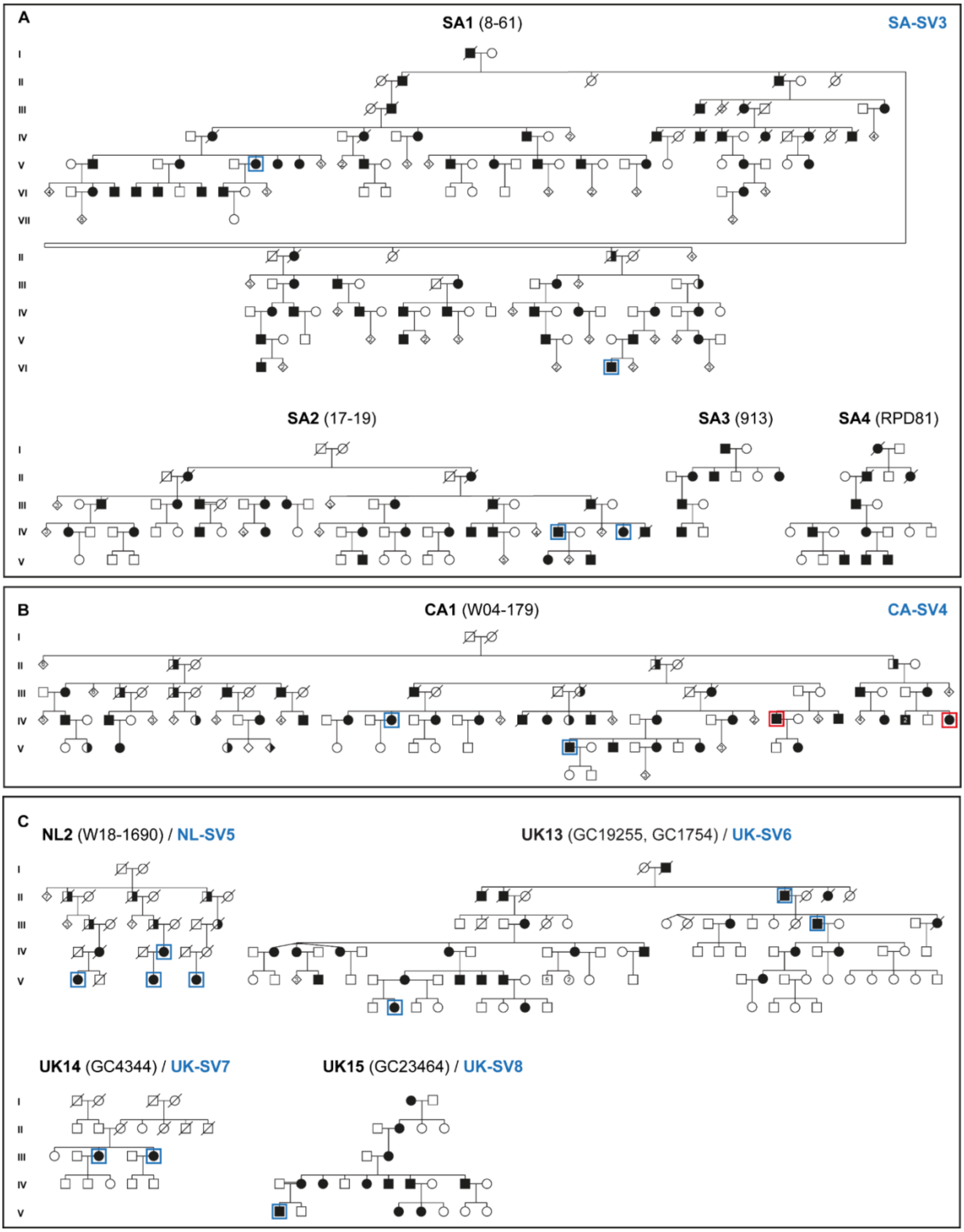

Figure S2: Additional adRP families with structural variants within the RP17-locus. (A) Pedigrees of South African origin with structural variant 3 (SA-SV3) **(B)** Canadian family with structral variant 4 (CA-SV4). **(C)** Dutch family (NL2) with structural variant 5 (NL-SV5), UK family (GC19255/GC1754) with structural variant 6 (UK-SV6), UK family (GC4344) with structural variant 7 (UK-SV7) and UK family (GC23464) with structural variant 8 (UK-SV8). WGS or WES was performed in individuals in blue or red, respectively.

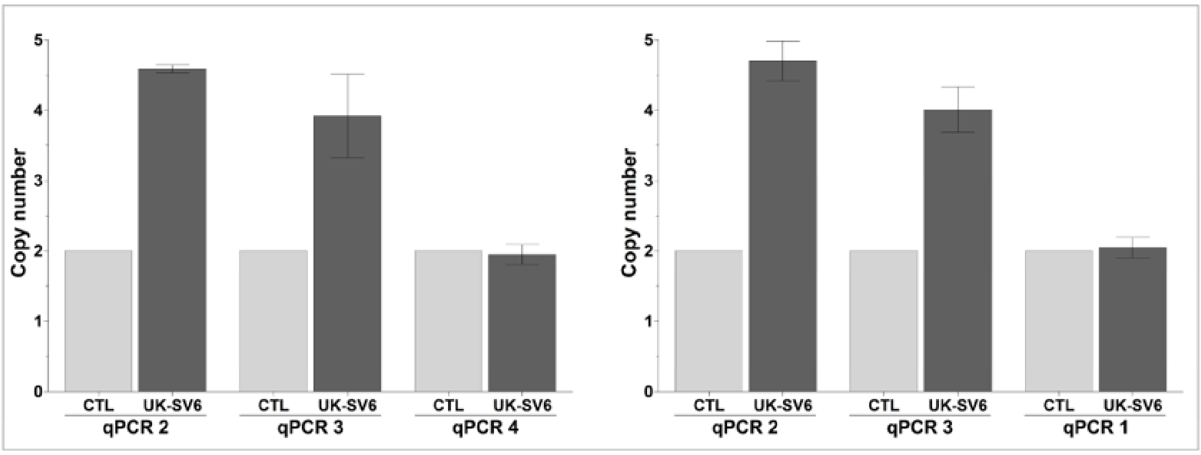

Figure S3: Triplication of region in UK-SV6. Quantitative real-time PCR for individuals from family UK13, UK-SV6. Primer pairs qPCR 2 (first intron of *GDPD1*) and qPCR3 (downstream of *YPEL2*) confirmed triplication (four copies in the genome) of UK-SV6, compared to control unaffected DNA samples and additional control qPCR assays for genomic regions distal and proximal to this structural variant qPCR4 (last intron of *LINC01476*) and qPCR1 (exon 3 of *SMG8*). CTL, unaffected control DNA sample; UK-SV6, affected individual DNA sample. Primer sequences are listed in Table S3.

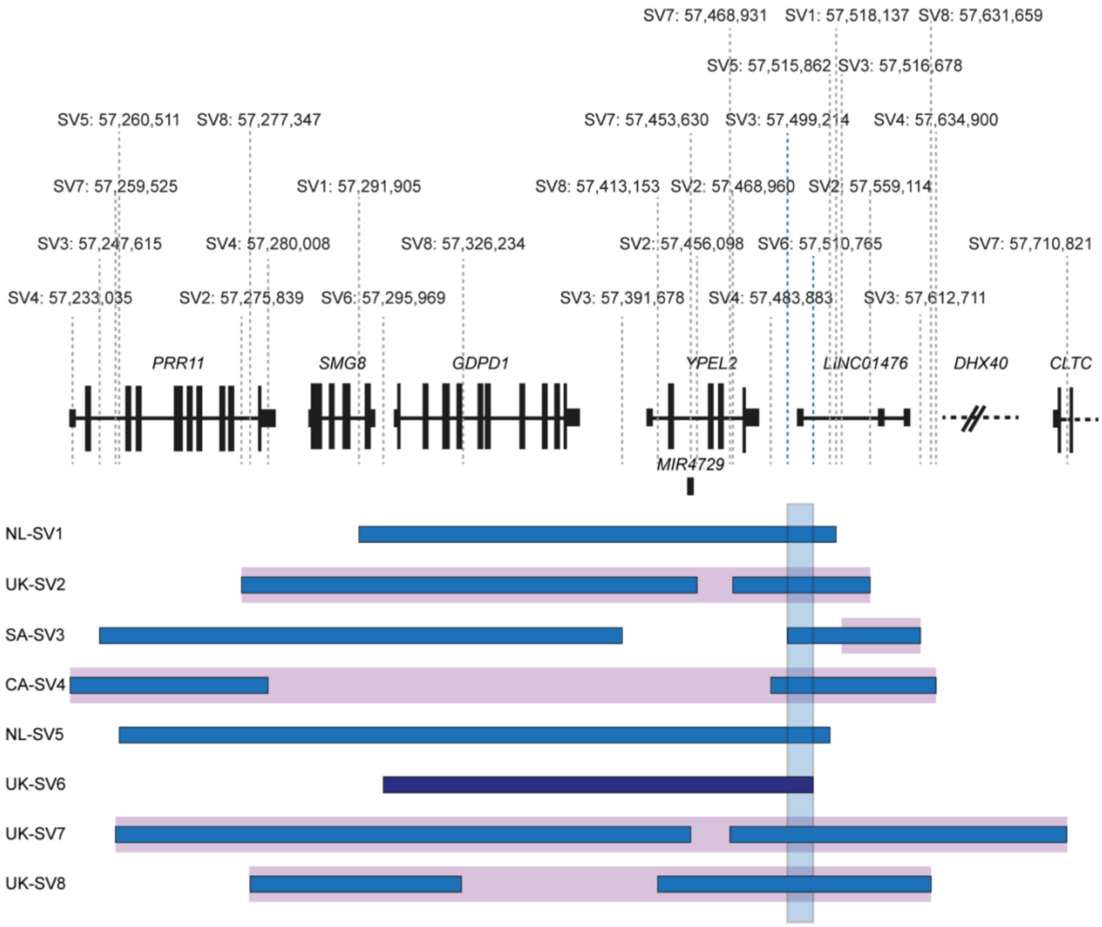

Figure S4: Overview of all SV breakpoints identified in the RP17-locus. Breakpoints are indicated with dashed lines. Duplicated, triplicated or inverted genomic regions for each SV are illustrated with blue, dark blue or purple bars, respectively. An overlapping genomic region that is duplicated or triplicated in all SVs was identified (chr17:57,499,214-57,510,765) and is highlighted by a light-blue vertical bar. The size of *DHX40* is reduced and *CLTC* is only partially shown in this figure.

A

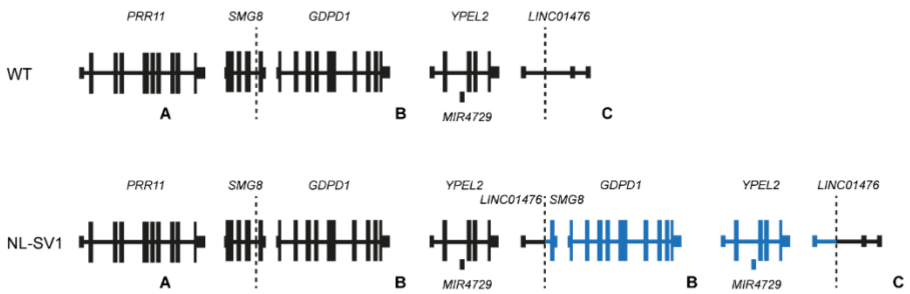


```
A-B   ---TATATATTCTTTTATTTATTTATTTGTTTGTTTTTTTGAGACAGTCTCGCTCTGTTG
B-B   -----AACCCTGTCTCAAAAAAAAAAAAATAAATAAATAAAGGAGAGTCA-----AGTCT
B-C   -----AACCCTGTCTCAAAAAAAAAAAAATAAATAAATAAAGGAGAGTCA-----AGTCT
            *   * * * *   *   *    *   *   *    ** ****       **

A-B   CACAGGCTGGAGGGCAGTGGTGC-AATCTCAGCTCACTGCACCTCCGCCTCCTGGGTTCA
B-B   C-------TCAGGGCCTTGGTTTACTTATCTGTAAAATGAAGGT-----ATTGGGCTAGA
B-C   C-------TCAGGGCCTTGGTTTACTTATCTGTAAAATGAAGGT-----ATTGGGCTAGA
      *         *****  ****     * ** *   * ** *  *         ** *  *

A-B   AGCTGTTCTCCTGCCCCAGCC---------TCCCCAGTAACAGATTACAGGCACACGCCA
B-B   AGATTCCTAGCAGCCCTAACAGGTTAACTAACAGCCAACTTGGGAGAAAGGCACACGCCA
B-C   AGATTCCTAGCAGCCCTAACAGGTTAACTAACAGCCAACTTGGGAGAAAGGCAAAGAGAC
      ** *      * **** * *           *  *       *   * ***** *    *

A-B   CCACACCTGACTAATTTTTTGTATTTTTAGTAGACATGGGGTT----TCTCCATGTTGGC
B-B   CCACACCTGACTAATTTTTTGTATTTTTAGTAGACATGGGGTT----TCTCCATGTTGGC
B-C   AAGAATTCCAACGCTGGCTACTTGAC---AGCACAATGTGACTGGAGTCTCTTGGCTGAC
          *    *   *  *             *** *  *    ****   * ** *

A-B   CAGGCTGGTCTCGAACTCCT--GACCTCGTGATTCACCTGCCTCAGCCTC-CCAAAGTGC
B-B   CAGGCTGGTCTCGAACTCCT--GACCTCGTGATTCACCTGCCTCAGCCTC-CCAAAGTGC
B-C   TTGTATTCTCTCACTGGTCTCAGCCCAAATGCTTGCCCTGATATGGTACTAAGTTATCCC
        *  *  ****      **  * **   ** **  ****     *         *   *

A-B   TGGAATTACAGGCGTGAGCCA-----
B-B   TGGAATTACAGGCGTGAGCCA-----
B-C   TTCCTAGAAAAGTTGGTGCCTCACAT
      *      * * *   * ***
```

Figure continues on next page

B

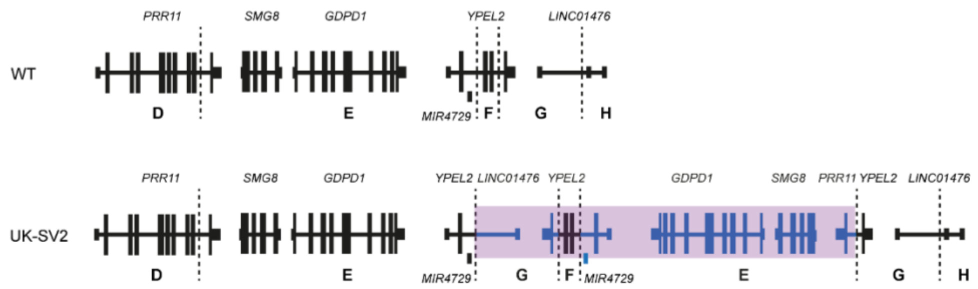


```
E-F    -CCAG-GAGTTTGAGACCAGCGTGGCCAACATGACAAAACCCTGTCTCCA
E-[G]  -CCAGGAGTTTGAGACCA-GCGTGGCCAACATGACAAAACCCTGTCTCCA
[G-H]  GGCAATGGTATAAATAAATGAACTCACTCAAACTTAATTTCCTAATTATG
         **       *        *      *   *     **   ***   *

E-F    CTAAAAAAATACAAAAATGGCCGGGCACGGTGGCTCACGCCTGTAATCCC
E-[G]  CTAAAAAAATACAAAAATGGCCGGGCACGGTGGCTCACGCCTGTAATCCC
[G-H]  GAGGAAATCTAGCACAGAGATTAAAAGCATAGGCTCTGGACTTAAC-TCT
           ***  **  * *  *        *   *****  * **  *   *

E-F    AGCACTTTGGAAGGCCGAGGCAGGTGAATCATGAGCTCAGGAGTTTGAGA
E-[G]  AGCACTTTGGAAGGCCGAGGCAGGTGAATCATGAGCTCAGGAGTTTGAGA
[G-H]  AGTTTGTGGGGAGTCCTGGACTT--TCCAGTGACTTGATAGGTGGCCTTA
       **    * ** ** **  * *                     *       *

E-F    CCAGCCTGACCAACATGGTGAAACCCCGTCTCTACCCCAAATACAAAAAT
E-[G]  CCATCCTCATTTGTGAAATAGGGCAAATAATTATGCGGTATC---AACCT
[G-H]  GTTTCCTCATTTGTGAAATAGGGCAAATAATTATGCGGTATC---AACCT
           *** *         *    *      *    *   *     **  *

E-F    TAGCAAGGTGTGGTGGCGGAT-------GCCTGTAGTCCCAGCTACTCGG
E-[G]  AATAAGGTTGTGGTGAGCACTGGATGAGAAATGCATAGAAAGCTTCTAAA
[G-H]  AATAAGGTTGTGGTGAGCACTGGATGAGAAATGCATAGAAAGCTTCTAAA
        *  * * *******     *          ** *     **** **

E-F    GAGGCTGAGGCAGGAGAATCACTTGAACCT-------GGGAGGCAGAGGT
E-[G]  ACAGTGCCTGGCAGTCCATAAATGTTAACTATTACTATCACGAAGGCTGT
[G-H]  ACAGTGCCTGGCAGTCCATAAATGTTAACTATTACTATCACGAAGGCTGT
          *     *   *   ** * *   * **           *   *  **

E-F    TGCAGTGAGCCAAGATT
E-[G]  TATTCTG----------
[G-H]  TATTCTG----------
       *    **
```

```
[D-E]  AATCTTGGCTCACTGCAACCTCTGCCTCCCAGGTTCAAGTGATTCTCCTG
[E]-G  AATCTTGGCTCACTGCAACCTCTGCCTCCCAGGTTCAAGTGATTCTCCTG
F-G    ---ATG-----TGAGCAGTACATGCCAGGCACTGAATTATGATTCCATTG
           *         ***      ****    **         ******   **

[D-E]  CCTCAGCCTCCCGAGT------AGCTGGGACTACAGG-CATCCGCCACCA
[E]-G  CCTCAGCCTCCCGA------GTAGCTGGGACTACAGG-CATCCGCCACCA
F-G    AAATAGTTTGAAAATGTGGAAATCATTAAAAAAAAAAATTGTCCCAGGTG
           **   *    *           *    *   * *       * *

[D-E]  CACCTTGCTAATTTTTGTATTTGGGGTAGAGACGGGGTTTCACCATGTTG
[E]-G  CACCTTGCTAATTTTTGTATTTGGGGTAGAGACGGGGTTTCACCATGTTG
F-G    GAGCCTGGTAGCACTGCCTTTAGATTCCATTTTCCCCATCTCCCTGGAAA
         * * ** **     *    ** *                *   **  *

[D-E]  GTCAGGCTGGTCTCAAACTCCTGAGCTCATGATTCACCTGCCTCGGCCTT
[E]-G  GTCAGGCTATTACAAACAAAAATGGGGCCAGGCGTAATAGCTCAGGCCTA
F-G    CATTCAGGATTACAAACAAAAATGGGGCCAGGCGTAATAGCTCAGGCCTA
                *   **        *  *  *    *   **   *****

[D-E]  CCAAAGTGCTGGGATTACAGGCGTGAGCCACCGTGCCCGGCCATTTTTGT
[E]-G  TAACCCCAGCAGTTTGGGAGGCCGAGGTG--GGTGGATCACCTGAGGTCA
F-G    TAACCCCAGCAGTTTGGGAGGCCGAGGTG--GGTGGATCACCTGAGGTCA
         *        *  *   ****    *      ***      **     *

[D-E]  ATTTTTTTAGTGGAGACAGGGTTTTGTCATGTTGGCCACGCTGGTCTCAA
[E]-G  AGAGTTCGAGAC---------------CCGCCTGGCCAACATATAGTGAA
F-G    AGAGTTCGAGAC---------------CCGCCTGGCCAACATATAGTGAA
       *   **  **               *     ******   *    * **

[D-E]  ACTCCTGG------------------
[E]-G  ACTCCGTCTCTACCAAAAATAAAAAT
F-G    ACTCCGTCTCTACCAAAAATAAAAAT
       *****
```

Figure continues on next page

C

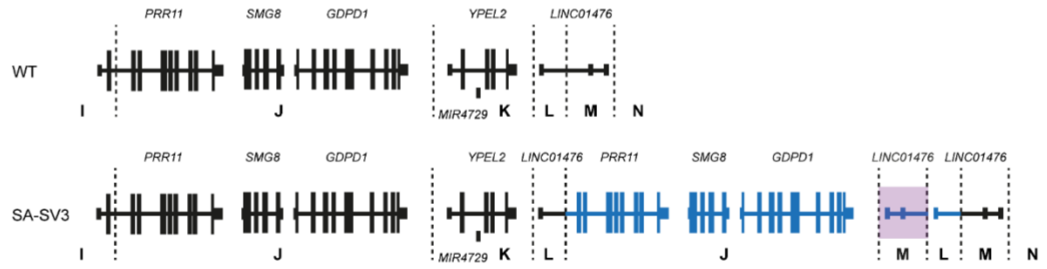


```
I-J     ----------TTAG----CCTGCGTGGTGGCGGGCAACTGTAATCCCAGC
L-J     CAGAGCTGATTTAGTAGGAATGGGTTGGGCCTGAGATCCTGCATTTCTTT
L-M     CAGAGCTGATTTAGTAGGAATGGGTTGGGCCTGAGATCCTGCATTTCTTT

I-J     TTCTCGGGAGACTTAGGCAGGAGAATTGCTTGAACCAGAGAGGCAGAGGT
L-J     GTCTTTGT----TTCTTCTCTCTCTTTCTTTCTTCCTTTTCTTTCCTTCC
L-M     GTCTTTGT----TTCTTCTCTCTCTTTCTTTCTTCCTTTTCTTTCCTTCC

I-J     TGCAGTGAGCCGAGATCACACCACTGCACTCAAGCCTGGGCAACAAGAGC
L-J     TTCCTTCGCTTTCTTTCTTTCCTTTTTTT---TTTTTTTTTTTCTAAATG
L-M     TTCCTTCGCTTTCTTTCTTTCCTTTTTTT---TTTTTTTTTTTCTAAATG

I-J     GAGACTCCCTCTCAGAAAAAAA-----------AAAGAAGGCCCAGTTAA
L-J     GAGTTTTG------GAAAAAAA-----------AAAGAAGGCCCAGTTAA
L-M     GAGTTTTG------CTCTGTTGTGCAGTGGTGCAGTCACAGCTCACTGCA

I-J     ATTTGAATTTCAGATAAACAATGAATAATTTTTT--CATATAATTATGTC
L-J     ATTTGAATTTCAGATAAACAATGAATAATTTTTT--CATATAATTATGTC
L-M     ATCTCAAACTCCTGGGCTCAAGCAACCCTCCCACCTCAGCTTCTCAAAGG

I-J     CTATGTGACATTTGGAATATATTTATACTAAAATTATGCATGCTTTATCT
L-J     CTATGTGACATTTGGAATATATTTATACTAAAATTATGCATGCTTTATCT
L-M     AGCTGAGACTATAGGCGTCTAT---------CACCATGCCTGG-------

I-J     GAAATTAATTTTTTATTTTAATTTTTAT
L-J     GAAATTAATTTTTTATTTTAATTTTTAT
L-M     ---CTAGATTCTGCATTGCCA-------

J-K     CTGCCTCAGCCTCCTAAAGTGCTGGGATTACAGGCATGAGCCACTGCACCT
J-[M]   CTGCCTCAGCCTCCTAAAGTGCTGGGATTACAGGCATGAGCCACTGCACCT
[M-N]   ------CACTGCACTCCAGTC-TGGGAAACAGAGCAAGAC----TCTGTCT

J-K     GGCAAGAGTAAATATTTTTAAATGAGACATTAAGACCTGGGTTTTGGCTGC
J-[M]   GGCAAGAGTAAATATTTTTAAATGAGACATTAAGACCTGGGTTTTGGCTGC
[M-N]   CCAAAAAATAAAAAATAAAAATAAAAATAAAAGAAAATTCCTCACAAACGA

J-K     GTACAGTGGCTCACGCCTGTCATCTC---------AGCTCTTTGGGAAGTCA
J-[M]   GTACAGTGGCTCACGCCTGTCATCTC---------AGCTCTTTGGGAAGTCA
[M-N]   CTTCTGTAATCACCCCCTCTCCTGATTCATTATTTTTTCTTTAAAAACGTA

J-K     AG--TCAG-------------------------------------------
J-[M]   AG--TCAGCCAGGCTCAGTGGCTTACTCCTGTAATCCCAGCACTTTGGGAG
[M-N]   AGCCTCAGCCAGGCTCAGTGGCTTACTCCTGTAATCCCAGCACTTTGGGAG

J-K     ----------GAGGATCATCAGCAGTCAGGTGTTTGAGACCAGCCTGGCCA
J-[M]   ACTGAGGCGGGCGGATCAACTGAGGTTGGGAGTTCGAGACCACCTTGACCA
[M-N]   ACTGAGGCGGGCGGATCAACTGAGGTTGGGAGTTCGAGACCACCTTGACCA

J-K     ACATGGCAAAACCCTGTCTCTACTAAAAATACAAAAATTAGCCAAGCATAT
J-[M]   ACATGGAGAAACCCCGTCTCTACTAAAAATACAAAATTAGCCGGGTGTGGT
[M-N]   ACATGGAGAAACCCCGTCTCTACTAAAAATACAAAATTAGCCGGGTGTGGT

J-K     TGGGTACCTGTAATCCCAGCTACTCGGGAGGGTGAGGCAGGAGAATTGCTT
J-[M]   GGCACA---------------------------------------------
[M-N]   GGCACA---------------------------------------------
```

```
[L-M]   -----TGGCAATGCAGAATCTAGCCAGGC-----ATGGTGATAGACGCCTATAGTCTCAGCTCCTTTGAGAAGCTGAGGTGGGAGGGTTGCTTGAGCCCAGGAGTTTGAGAT
[M]-L   -----TGGCAATGCAGAATCTAGCCAGGCATGGTG-----ATAGACGCCTATAGTCTCAGCTCCTTTGAGAAGCTGAGGTGGGAGGGTTGCTTGAGCCCAGGAGTTTGAGAT
K-L     CTCAGGTGATCTGCCCACCTCGGCCTCCCAAAGTGCTGAGATTACAGGCATGACCCA---CCACGCCCAGCCTATGAACTATGAGACTTCTGA-------GAACTGTTAACA

[L-M]   TGCAGTGAGCTGTGACTGCACCACTGCACTCCAGCCTGGGCAACAGAGCAAAACTCCATTTAGAAAAAAAAAAAAAAAAAAAAGGAAAGAA------AGAAAGCGAAGGAAGGAA
[M]-L   TGCAGTGAGCTGTGACTGCACCACTGCACTCCAGCCTGGGCAACAGAGCCTGTCACGGTCCAGATTGTGCCACTCTTTGGAATTTCTGAATGGTATGAATCCAGAGGAGAGAA
K-L     GGGAGGATAAATGGAGCCAGGAGACGCCTCACTTTGGCTTCAGTGGAACCTGTCACGGTCCAGATTGTGCCACTCTTTGGAATTTCTGAATGGTATGAATCCAGAGGAGAGAA

[L-M]   GGAAAGAAAAGGAAGAAAGAAAGAGAGAGAAGAAACAAAGACAAAGAAATGCAGGATCTCAGGCCCAACCCATTCCTACTAAATCAGCTCTG
[M]-L   ACAACGTTCTGTTTGTTCTCATG-GGCCCTCATACCACAGACGTTTACATCATGGGCCTGC----ACACTGAGAGGTGCTGGTCATGTTTT-
K-L     ACAACGTTCTGTTTGTTCTCATG-GGCCCTCATACCACAGACGTTTACATCATGGGCCTGC----ACACTGAGAGGTGCTGGTCATGTTTT-
```

Figure continues on next page

D

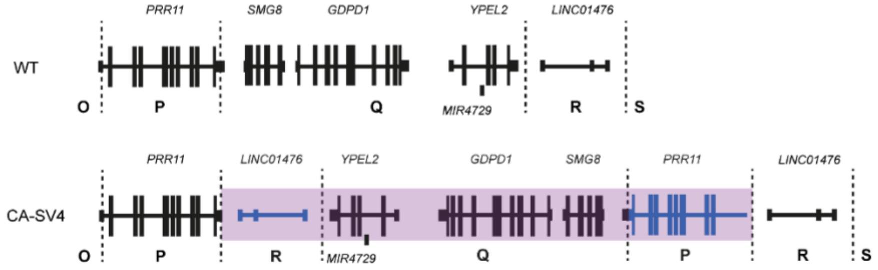


```
P-Q     -CACTATAATTATGCCTGTGAATAGCCACTCTACTCC-AGCCTGGGCAAC
P-[R]   -CACTATAATTATGCCTGTGAATAGCCACTCTACTCC-AGCCTGGGCAAC
[R-S]   AGTGTATAGT-------TTTGTTACCCACACAATGGGTGGGTTTGATCAC
            **** *       *   ** **** * *       *  * *   **

P-Q     ATAGCAAGACCCCATCTCTAAAAATTAAAAAAAAAAT--TTAAATTAGAA
P-[R]   ATAGCAAGACCCCATCTCTAAAAATTAAAAAAAAAA-AT-TTAAATTAGAA
[R-S]   TTAGCCAGTGACAGTCCAATACCACAACCAAGGAAGATTTAACAAGGGGA
         **** **   *  **    *  *  *  **  **     * * *   * *

P-Q     TATCATTTCTA--GCATCTTAGGTAGGTACTTATATCTGGCTTACAGAAG
P-[R]   TATCATTTCTA--GCATCTTAGGTAGGTACTTATATCTGGCTTACAGAAG
[R-S]   TTTTATTACTTACAATAAGTAAGGAGGATATCGGGGATAAATCCCCAAAG
        * * *** **             ** * ***   *      *   *  *  ***

P-Q     TCTAAGGTATTCCTTATTTTTATATCTGCTGTCCACATTTATACAGCTAC
P-[R]   TCTAAGGTCCCTAAACAAAGGTGATAAAAAGGC---TTTTATTAGGCAGG
[R-S]   CAGTGTCTCCCTAAACAAAGGTGATAAAAAGGC---TTTTATTAGGCAGG
               *              **     * *    *****   **

P-Q     ATAAAAATTTTATGACA-----ACTTCAACATGAACCTTATATTTTCGAC
P-[R]   TTAGCTGAGTTATCATATGTAGAGGTGGAGTAGAGCCAGCTCAGGCCCAG
[R-S]   TTAGCTGAGTTATCATATGTAGAGGTGGAGTAGAGCCAGCTCAGGCCCAG
         **      **** * *     *  *  *   ** **   *      * *

P-Q     AATGCCTTGCCAAGGAATCTCTGAAGTCCATAGCAGGTCACTGTGAGACC
P-[R]   TCTGCGATC-----ACGCCTCTACATACAAATGGAGA-ATAGCTCCTTCC
[R-S]   TCTGCGATC-----ACGCCTCTACATACAAATGGAGA-ATAGCTCCTTCC
          *** *         **** *     *  * **        *     **

P-Q     TAGTTCCCTGTT-----
P-[R]   TGGGTGGATTTTTAGTA
[R-S]   TGGGTGGATTTTTAGTA
        * * *   * **
```

```
[O-P]   ------TAATCGTTCCTGCACTTACTAGTCTAAGAGCCGGGGGAGTCGAT
[P]-R   ------TAATCGTTCCTGCACTTACTAGTCTAAGAGCCGGGGGAGTCGAT
Q-R     ATGCACTCATCATCCCTGCACTCTGG--TCTGGCATGCAGGTAACGTGCT
              * *** * ********       ***    *   * **   *    * *

[O-P]   GGAGTCCGGGCTGTGGCCTCTCCCACGCCATAAAGAAAAATTCAAAATTA
[P]-R   GGAGTCCGGGCTGTGGCCTCTCCCACGCCATAAAGAAAAATTCAAAATTA
Q-R     GGAGAGGAGGA--GGCCCAATGCTGTCAAATTCACTCACCTCCCC---AG
        ****     **     * **   * *       **   *    *   * *

[O-P]   AATCCTTGTCAGGGAGAGGCCAATCGTAGACACGGTCTCTGCACGATTCG
[P]-R   AATCCTTGTCAGGGAGAGGCCAATCGTAGACACGGTCTCTGCACGATTCG
Q-R     CTTCCCCTGGACGTGTCTGCCTCAAATTCTGAGCCACTTCGCCAGAGCAA
          ***      * *     ***      *     *     **   **   **

[O-P]   CTCTTCGGATGGTGTAGGAGCACAGCCCGGCTTGTGATTGGCTGAGAT-A
[P]-R   CTCTTCGCAAGCAAAGCAACCAAGCAGTCGAATCGGGGATGTTTTATTTA
Q-R     GGCCACGCAAGCAAAGCAACCAAGCAGTCGAATCGGGGATGTTTTATTTA
          *  ** * *        * **        *   *   *     * *    * *

[O-P]   GCACAGAGCTCCAGCCAATCAGAGCTGAGGCCGTTCTGGTATGAATAAGG
[P]-R   TGGCTTTAATAA---TGAAGCCTCTTGGGGAAATTTTAGTTTCCCATTCC
Q-R     TGGCTTTAATAA---TGAAGCCTCTTGGGGAAATTTTAGTTTCCCATTCC
           *     *        *        ** **    ** * ** *

[O-P]   AGAAGGCGGGAGAGAAGGGGAAGGAAAGCCGG-AG--GGGAAAGAAGGTG
[P]-R   CAGAAGCGAAAATGCGTCTACAGAATGTTCTTAAAATTTATTACTGTTTA
Q-R     CAGAAGCGAAAATGCGTCTACAGAATGTTCTTAAAATTTATTACTGTTTA
           * ***  *  *       ** *    *   *        *     *

[O-P]   TGTTACGTCCC
[P]-R   TGTTTTTTCCT
Q-R     TGTTTTTTCCT
        ****   ***
```

Figure continues on next page

E

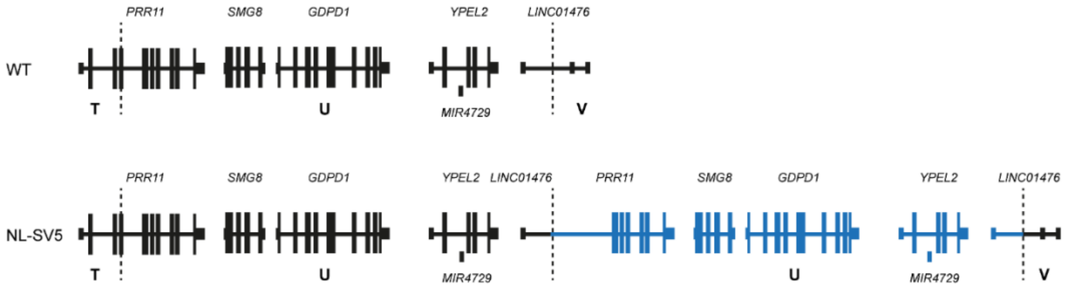


```
T-U    GCAGTTGTCCACTTGAGCTCCCAGATGCCCATGGAGTCCAGTCCTTCCAATCAGGAAGGT
U-U    -CAGATTTGTCCTGATCCGTCCACTTGCAATGCCCTTCTCTGTCTTTCCTTCTCCATTAA
U-V    -CAGATTTGTCCTGATCCGTCCACTTGCAATGCCCTTCTCTGTCTTTCCTTCTCCATTAA
        *** * *   **     *  ***  ***        **      ** *  ** ***

T-U    TGGAATCTCTGATGTCATTGGTCATTCCAACCTGGCAACCAGTTTGAAGAAAAACACATG
U-U    T--ACATGCTTATCACCTTATTCAAGAGCCAAGGAGAATCTCACCACCTGCAGGAAGACT
U-V    T--ACATGCTTATCACCTTATTCAAGAGCCAAGGAGAATCTCACCACCTGCAGGAAGACT
       *  *    ** **  * **  ***         *  ** *           *   * *

T-U    TAACTGCCAGGCTGGTCTCTTGTCCTGGAGATCCTGGGTGAATGGTATCTCCTGCCACTG
U-U    GCCCAGACTGACCCCTCTTAACTGTAATCAATCCTGGGTGAATGGTATCTCCTGCCACTG
U-V    GCCCAGACTGACCCCTCTTAACTGTAATC-ATCCTTTTTTTTTTTTTTCTTTTTTAAGA
          * * * * *   ***    *       *****   *   *  * *    *   *

T-U    TCCCAACCTCAGACCACCATCCAAAAGCATCTT------C-------AGGGTCTCCGCAT
U-U    TCCCAACCTCAGACCACCATCCAAAAGCATCTT------C-------AGGGTCTCCGCAT
U-V    -CAGAATCTCACTCTGTCATCTAGGCTGGAGTGCACTGGCCCGATCTGGGCTCACTGCAA
        *  ** ****  *   **** *                         ** **    **

T-U    CCATCTGTTCCCTGTCCCAGCAGAGGCTGTGTCTTCTCCACTCAAAGCCTGAAGCATTTT
U-U    CCATCTGTTCCCTGTCCCAGCAGAGGCTGTGTCTTCTCCACTCAAAGCCTGAAGCATTTT
U-V    CCTTCGCCTCCTGGGTTCAA---------GCAATTGTCCTGCCTCAGCCTCCAGAGTAGC
       ** **   ***  *   **              ** ***   *  *****  **  *

T-U    TGGGGTCTCC-----
U-U    TGGGGTCTCC-----
U-V    TGAGATTACAGGTGC
        * *  * *
```

Figure continues on next page

F

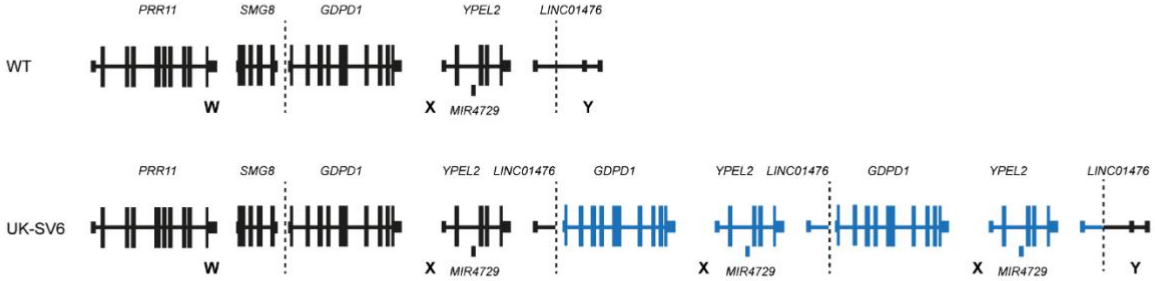


```
W-X   GTTAAGTTCTTCTGGGAGCCTTTGATTTTAGGTTGATAAGGGATTCCAGGAACTTAAATG
X-X   ----------------------TCAAATTAGCAATAAAAACAG-TACTATATATATATAT
X-Y   ----------------------TCAAATTAGCAATAAAAACAG-TACTATATATATATAT
                            * *  ****    * **     * *   *  *  *

W-X   CCTTCAGCTCAATATATATATATATTTTTTAAGAGACAGAGTCTTGCTCTGTCGCCCAGGCT
X-X   ATATTTTTAATTTTTAAAAATTTTTTTTTCAAGATGGAGTCTCGCTCTGTCACCCAGGCT
X-Y   ATATTTTTAATTTTTAAAAATTTTTTTTTCAAGATGGAGTCTCGCTCTGTCACCCAGGCT
         *        * ** * ** ******   ***  ****** ******** ********

W-X   GGGGTGCCGTGGCATGATCTTGGCTCACTGCAAGCTCCGCCTCCCAGGTTCAAGCCATTC
X-X   GGAGTGCAGTGGCACAATCTTGGCTCACTGCAACCTCTGCCCCCCAGGTTCAAGCCATTC
X-Y   GGAGTGCAGTGGCACAATCTTGGCTCACTGCAACCTCTGCCCCGCAGGTTCAAGTGATTC
      ** **** ******  ***************** *** *** * **********  ****

W-X   TCCTGCCTCAGCCTCCCTAGTAGCTGGGACTACAGGCATGCACCACTATGCCTGGCTTAT
X-X   TCCTGCCTCAGCCTCCCTAGTAGCTGGGACTACAGGCATGCACCACTATGCCTGGCTTAT
X-Y   TCCTGCCTCAGCCTCCCAAGTAGCTGGGATTACAGGCGTGCTGTAATT------------
      ***************** *********** ******* ***   * *

W-X   TTTTGTATTTTTAGTAGATACGGGGTTTCACCATGATGGCCAGGCTAGTCTCGAACTCCC
X-X   TTTTGTATTTTTAGTAGATACGGGGTTTCACCATGATGGCCAGGCTAGTCTCGAACTCCC
X-Y   -TTTGTACTTTTAGTAGAGATGGGGTTTCACCATCTTGGCCACGCTGGTCTTGAACTCTG
       ****** ********** * *************  ****** *** **** ******

W-X   G------------------------------------
X-X   G------------------------------------
X-Y   ACCTTGTGAGCCACCCGCCTCGGCTCCCGAAGTGCTC
```

Figure continues on next page

G

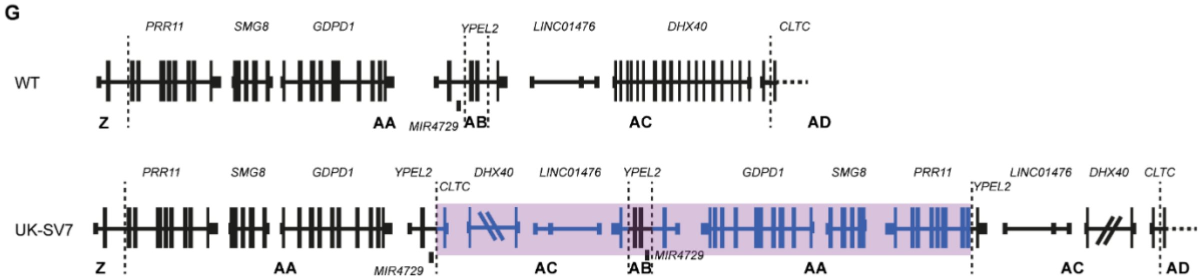


```
AA-AB   -GAAGTCATTGGGATGACACCTTCATGACCCCATCTTCATTCTTCCCTAG
AA-[AC] -GAAGTCATTGGGATGACACCTTCATGACCCCATCTTCATTCTTCCCTAG
[AC-AD] TTATTCTAGTTAGATCAATCAT---TTAAGGCATACTCAGTCTACCAGGT
           *    * *  *** *  * *   * *   ***  *** *** **

AA-AB   ACTTTAGAGCCAGACTGCCTGGAC--CTCTGTCATCATTTATTGATTGTG
AA-[AC] ACTTTAGAGCCAGACTGCCTGGAC--CTCTGTCATCATTTATTGATTGTG
[AC-AD] GCTA-CAAGTTATAAGACTAGAACCATTTTATTTTACAATTGAGTCACTA
          **    **  * *    *  * **   * * *  *     *     *

AA-AB   GGTTTGTTGTTGTTGTTGTTTTGAGATGGAGTCTCTCTCTGTCGCCCAGG
AA-[AC] GGTTTGTTGTTGTTGTTGTTTTGAGATGGAGTCTCTCTCTGTCGCCCAGG
[AC-AD] GAGTATTTTTTTTTCTTTTTTTGAGATGGAGTCTCGCTCTGTCGCCCAGG
        *  *  ** ** ** ** ***************** **************

AA-AB   CTGGCATGCAATAGCGTGATCTGGGCTCACTGCAATCTCTGCCTCCCAGG
AA-[AC] CTGGAGTGCAATGGCATGATCTCAGCTCACCACAACCTCTGCCTCCCGGG
[AC-AD] CTGGAGTGCAATGGCATGATCTCAGCTCACCACAACCTCTGCCTCCCGGG
        ****  ****** ** ******  ******  *** *********** **

AA-AB   TTCAAGTGATTCTCCTGCCTCAACCTCCCTAGTAGCTGGATTA-CAGGCA
AA-[AC] TTCAAGCCATTCTCCTGCCTCAACCTCCTGAGTAGCTGGGACTACAGGCA
[AC-AD] TTCAAGCCATTCTCCTGCCTCAACCTCCTGAGTAGCTGGGACTACAGGCA
        ******  ********************  **********     ******

AA-AB   CCCGCCACCACATCCGGCTAATTTTTGTATTTTTGATAG--AGACGGGGT
AA-[AC] TGCACCACCACGCCCAGCTAATTTTTGTATTTTTTCAGTAGAGACGGGGT
[AC-AD] TGCACCACCACGCCCAGCTAATTTTTGTATTTTTTCAGTAGAGACGGGGT
          * *******  ** ******************         *********

AA-AB   TTCACCA
AA-[AC] TTCAC--
[AC-AD] TTCAC--
        *****
```

```
[Z-AA]  GGCTCACACCTGTAATCCCAGCACTTTGGGAGGCCAAGGCGGGCAGATCA
[AA]-AC GGCTCACACCTGTAATCCCAGCACTTTGGGAGGCCAAGGCGGGCAGATCA
AB-AC   ----AAAAAATATATTTACACACATAT---AAAATAATGTGAGCAGTACA
             * *  * ** *  **   * *   *    ** * * ****  **

[Z-AA]  CTTGAGGCCAGGAGTT-TGAGACCA----GCCTGGTCAACATGGTGAAAC
[AA]-AC CTTGAGGCCAGGAGTT-TGAGACCA----GCCTGGTCAACATGGTGAAAC
AB-AC   TGCCAGGCACTGAATTATGATTCCATTGAAATAGTTTGAAAATGTGGAAA
            ****   ** ** ***  ***        * *  * *  *** **

[Z-AA]  CCCAT--CTCTACTAAAAGTACAAAATTGAGCTGGGCATTATGGCAGATA
[AA]-AC CCCAT--CTCTACTAAAAGTACAAAATTGAGCTGGGCATTATGGCAGATA
AB-AC   TCATTAAAAAAAAAAATTGTCCCAGGTGGAGCCTGGTAGCACTGCCT-TT
          *  *      *  **  ** * *  * ****  ** *  *  **   *

[Z-AA]  CCTGTAATCCCAGCTACCTGAGAGGCTGAGGCATGAGAATAACTGGAATC
[AA]-AC CCTGTAATTTTCCCCATCTCCCTGGAAACATTCAGGATTACAAACAAAA
AB-AC   AGATTCCATTTTCCCCATCTCCCTGGAAACATTCAGGATTACAAACAAAA
           *       *         *   *      ** **     **

[Z-AA]  CGGGAGACAGATGTTGCAGTGAACTGAGATTGCACCACTACACTCCAGCC
[AA]-AC ATGGGGCCAGGCGTAATAGCTCAGGCCTATAACCCCAGCAGTTTGGGA--
AB-AC   ATGGGGCCAGGCGTAATAGCTCAGGCCTATAACCCCAGCAGTTTGGGA--
          ** * ***  **   **   *     **  * ***  *   *

[Z-AA]  CGGGCGACAAAGCAAGATTCTGTCTCGAAACAAAAAAAAAAAAAACCAAT
[AA]-AC -GGCCGAGGTGGGTGGATCACCTGAGGTCA--AGAGTTCGAGACCCGCCT
AB-AC   -GGCCGAGGTGGGTGGATCACCTGAGGTCA--AGAGTTCGAGACCCGCCT
          ** ***    *   ***    *   *  *  * *     * *  *   *

[Z-AA]  AACAAAAA------
[AA]-AC GGCCAACATATAGT
AB-AC   GGCCAACATATAGT
          * ** *
```

Figure continues on next page

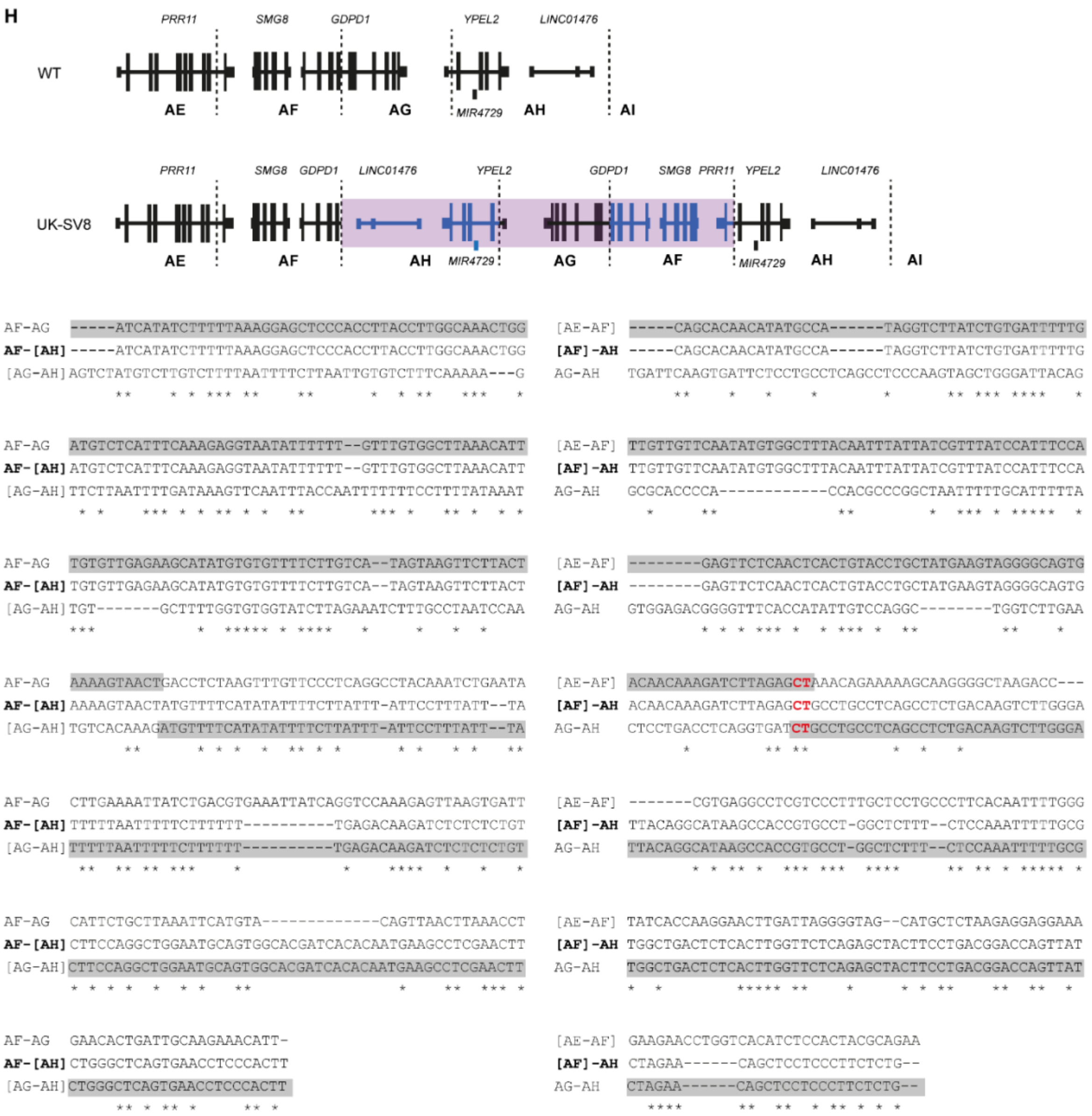

Figure S5: Assessment of microhomology at breakpoint sequences. (A-H) Breakpoint regions of structural variants (SVs) were assessed for presence of microhomology using multiple sequence alignment between the junction fragment and the 5' and 3' breakpoint regions using Clustal Omega. 150 bp reference sequences flanking each side of the breakpoint were used as input. Regions of microhomology are indicated in red. Breakpoint annotation of genomic regions as illustrated in Figure 2.

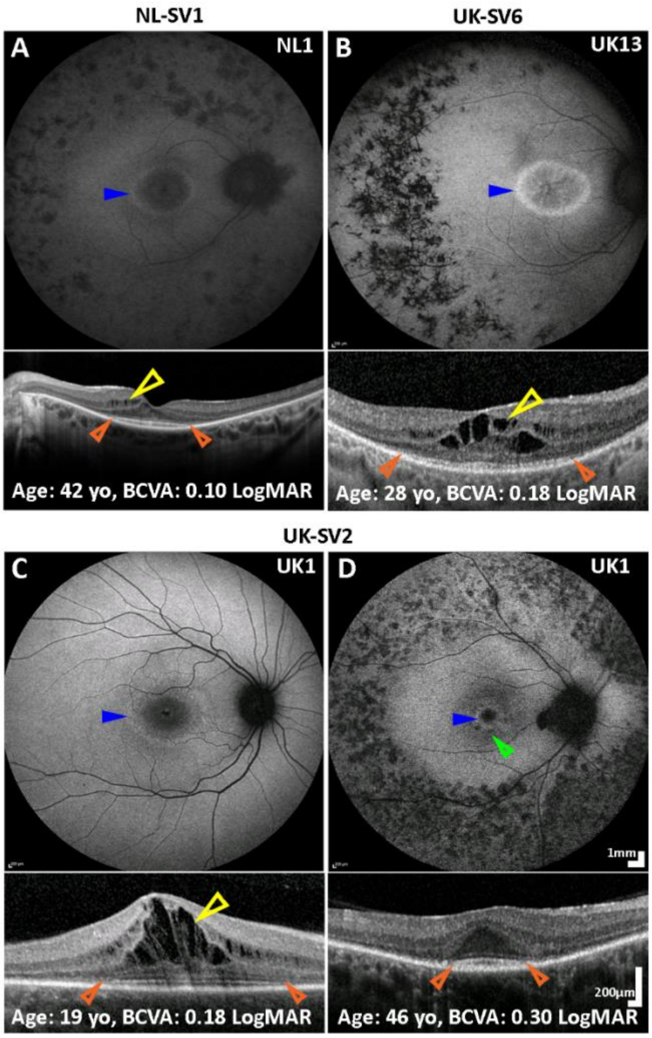

Figure S6: Detailed Retinal Imaging with Fundus Autofluorescence (FAF) and Optical Coherence Tomography (OCT). The blue arrow heads mark the temporal border of the ring of increased signal where present. The yellow arrow heads mark areas of cystoid macular edema **(A, B, C)**. The orange arrow heads mark on the OCT scans the border of the residual ellipsoid zone (EZ). Bone spicules are visible in the mid periphery on FAF in cases **(A, B, D)**. **(A)** Affected individual from family NL1 (NL-SV1). **(B)** Affected individual from family UK13 (UK-SV6). Affected individuals shown in **(C)** and **(D)** are from UK1 (UK-SV2). Two consecutive generations are shown, mother **(C)** and daughter **(D)**. Note the slow structural disease progression indicated by these cases, with minimal change in the BCVA, due to sparing of the foveal EZ (orange arrow heads). The ring of increased signal decreases in size over time (blue arrow heads), with small areas of decreased signal (atrophy) developing and increasing in these same regions over time (green arrow heads). yr, years old; BCVA, best corrected visual acuity; LogMAR, Logarithm of the minimum angle of resolution.

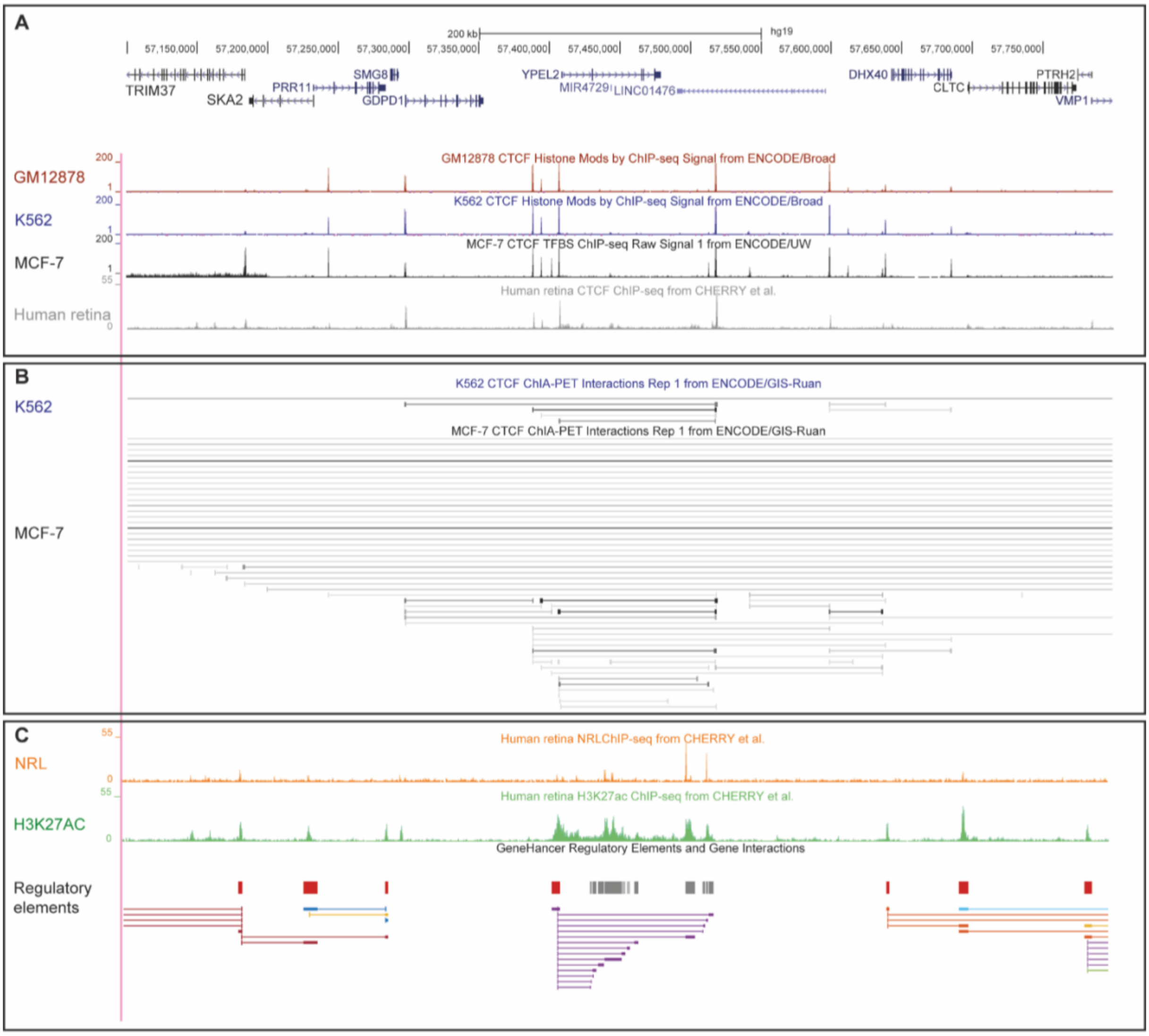

Figure S7: CTCF sites insulate contacts within the *YPEL2* TAD. (A) CTCF ChIP-seq data showed that *YPEL2* is located within an insulated TAD that is present in multiple cell and tissue types, including human retina. **(B)** ChIA-PET CTCF interaction data established in K562 and MCF-7 cells revealed strong interactions between the CTCF binding sites on the 5' side of the *YPEL2* TAD, and the single CTCF binding site on the 3' side of the *YPEL2* TAD. **(C)** The structured *YPEL2* TAD contains retina-specific enhancer elements as shown in Figure 3. These regulatory elements are also described in the Genehancer database[1] and interactions with the *YPEL2* promoter region were experimentally validated.

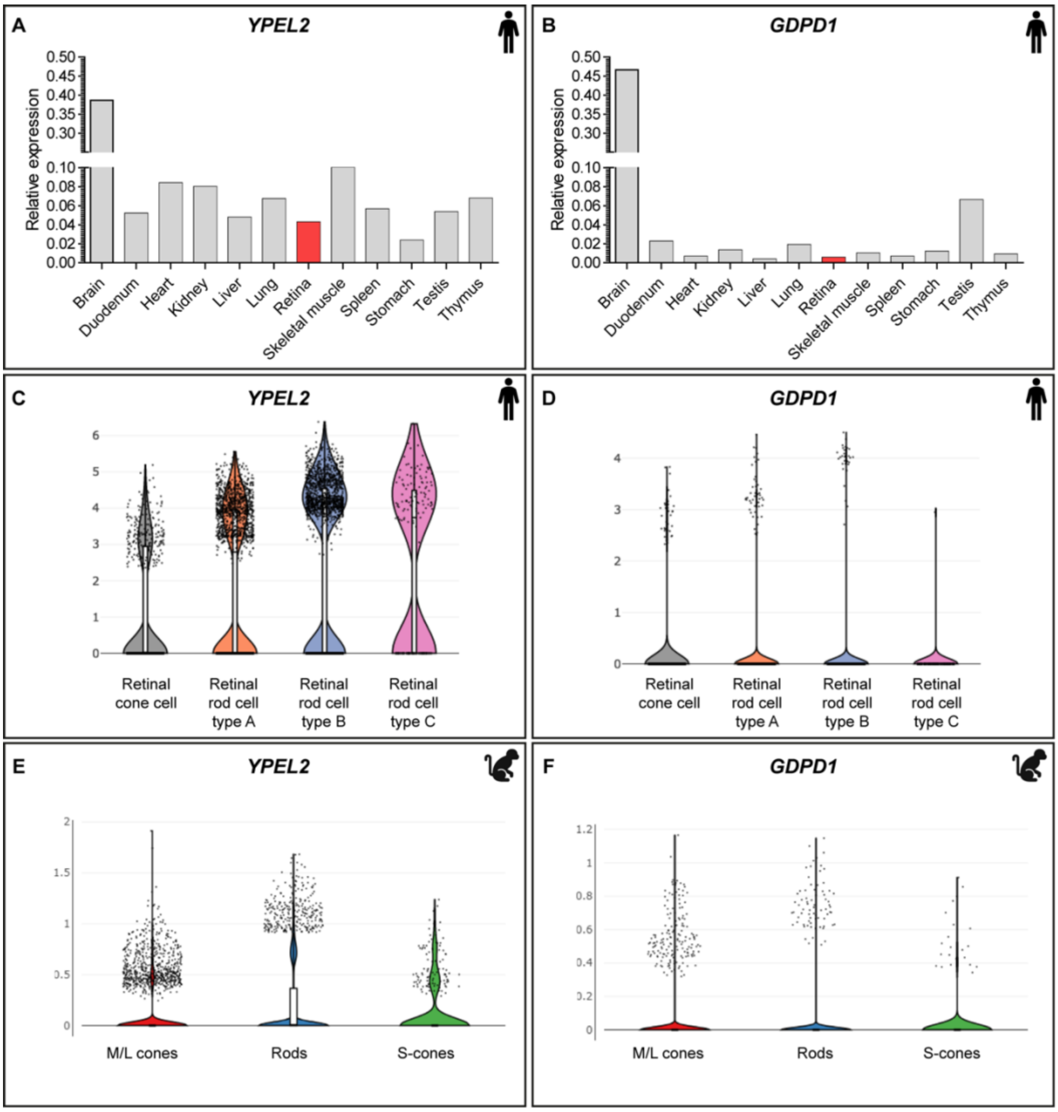

Figure S8: *YPEL2* and *GDPD1* expression across tissues and retinal cell types. (A) qPCR expression levels of *YPEL2* across healthy human tissues. *YPEL2* is ubiquitously expressed in the tissues studied, including retina, with highest expression in brain. **(B)** *GDPD1* is detected at low levels in all tissue types, with higher levels of expression in brain and testis. (**C-F)** *YPEL2* has higher levels of expression in rod photoreceptor cells compared to cones from single cell RNA sequence data. *GDPD1* has low levels of expression in all photoreceptor cells. Single cell expression levels and plots were obtained from the Broad Institute Single Cell Portal, and is based on single cell RNA sequencing results of human[2] (C-D) and macaque[3] (E-F) retinal cell types.

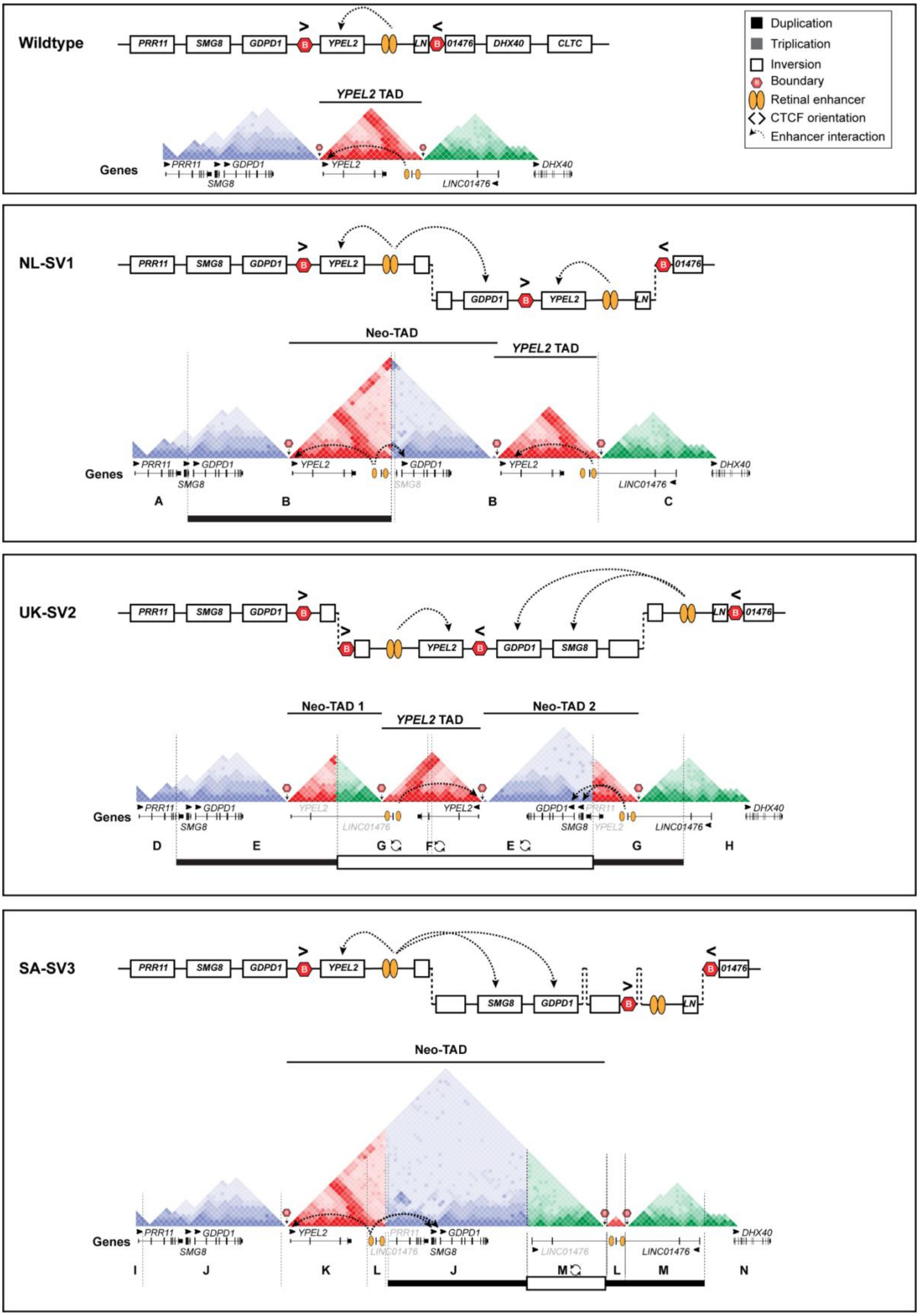

Figure continues on next page

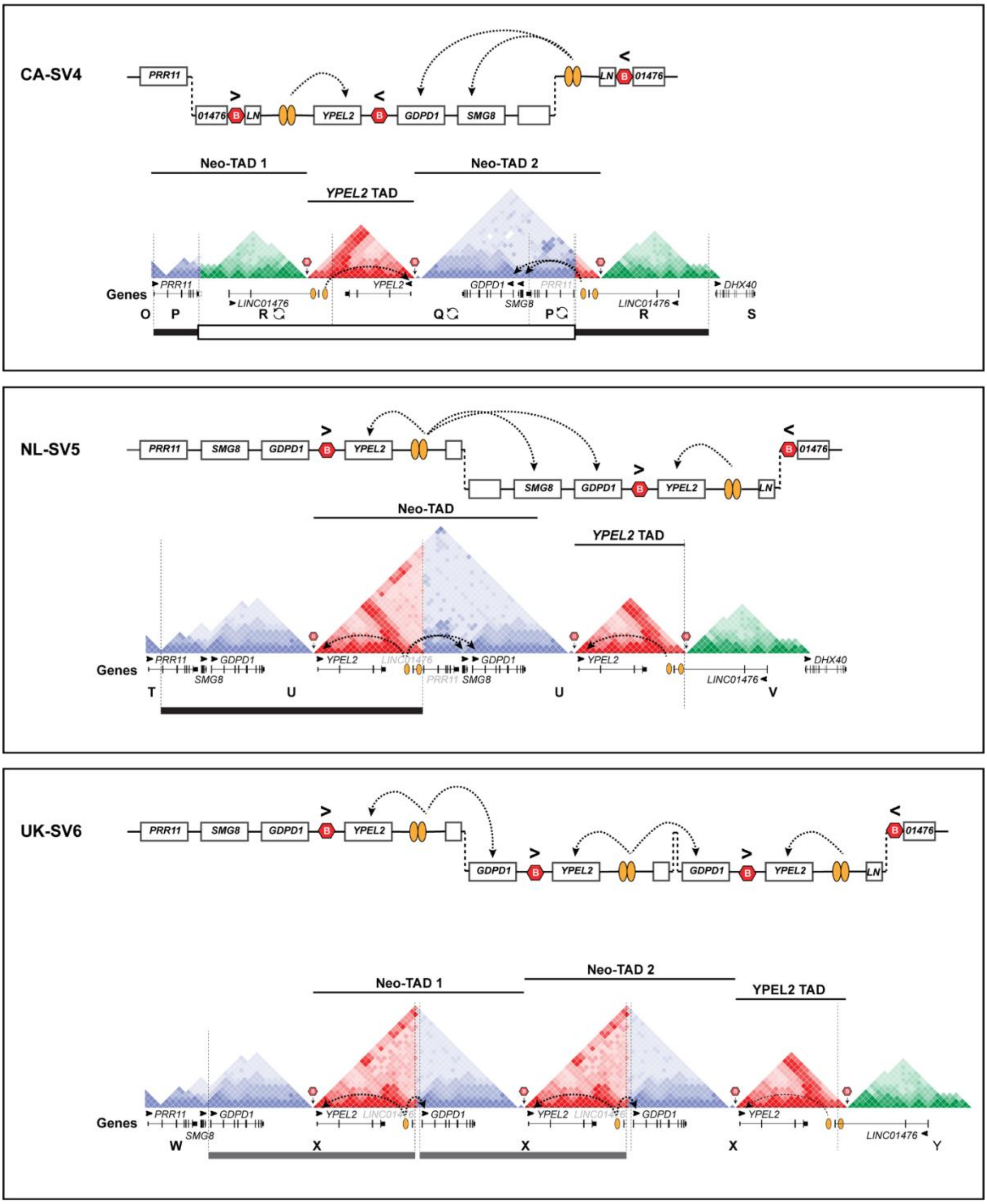

Figure continues on next page

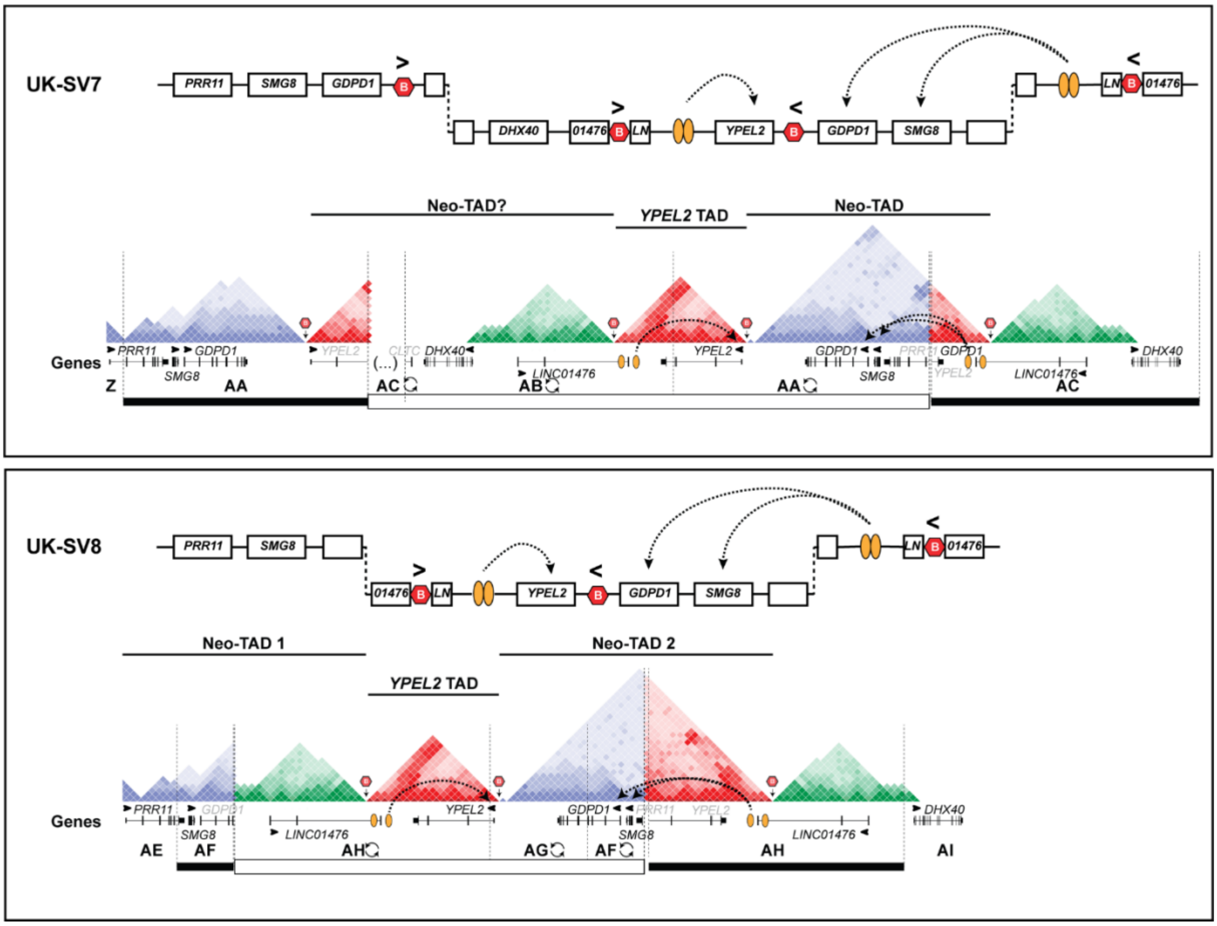

Figure S9: RP17-SVs are predicted to disrupt 3D chromatin organisation and create neo-TADs with ectopic retinal enhacer-gene contacts. Modelling of TAD boundaries, CTCF site orientation, gene position and orientation and retinal-specific enhancers for each unique RP17-SV is shown. Wild type chromatin organisation is depicted schematically, based on Hi-C maps. Schematic models of the genome architecture for each RP17-SV is shown above Hi-C map models (dotted vertical lines represent SV breakpoints). Shaded bars represent duplicated (black) or triplicated (grey) regions, whereas inversions are indicated by open bar below the TAD maps, with nomenclature corresponding to those described in Figure 2. In all RP17-SVs, new domains (neo-TADs) are created with ectopic contacts between retinal-specific enhancers and *GDPD1*. For NL-SV1, NL-SV5 and UK-SV6, an extra copy of *YPEL2* is also introduced into the neo-TAD. For UK-SV2, SA-SV3, CA-SV4, NL-SV5, UK-SV7 and UK-SV8, one copy of *SMG8* is introduced into the neo-TAD.

Table S1: Previously reported *CA4* variants

Genome	cDNA	Protein	Ethnicity	gnomAD AF all	gnomAD AF subpopulation	CADD_PHRED	Detection method	References
g.58227429G>A	c.4C>T	p.Ala12Thr	Chinese	0.000004471	- (other EAS)	6.280	Targeted sequencing of *CA4*	[4]
g.58227435C>T	c.40C>T	p.Arg14Trp	South-African	0.0002410	0.0001368 (AFR)	15.94	Locus gene sequencing (RP17)	This study (SA1-4), [5], [6]
g.58234014G>A	c.206G>A	p.Arg69His	Chinese	0.00004374	0.0001087 (other EAS)	0.005	Targeted sequencing of *CA4*	[7]
g.58235718C>A	c.655C>A	p.Arg219Ser	Northern European	0.00003186	0.0001163 (NWE)	26.6	Targeted sequencing of *CA4*	[6]
g.58235763G>A	c.700G>A	p.Val234Ile	Spanish	0.01015	0.01757 (NWE)	9.468	Targeted sequencing of 12 adRP genes	[8]

Overview of *CA4* (NM_000717.4) variants reported in literature. A CADD_PHRED score of ≥15 and allele frequency ≤0.0001 are considered as pathogenicity criteria. Values that meet these criteria are indicated in red. The p.Arg14Trp variant was found in families SA1-4 that are included in this study and carriers of SA-SV3. Genome, genomic position based on hg19; gnomad AF all, allele frequency in gnomAD v.2.1.1 database; gnomAD AF subpopulation, allele frequency in gnomAD v.2.1.1 based on subpopulation corresponding to the ethnicity of the affected individual in which the variant was reported in literature; CADD_PRED, Combined Annotation Dependent Depletion PHRED score; other EAS, other East Asian population; AFR, African population; NWE, Northwestern European population.

Table S2: Primer sequences utilized to validate and characterize breakpoints

SV	Breakpoint	Coordinates	F primer (5'-3')	R primer (5'-3')	Amplicon size (bp)
**NL-SV1**	A-B	57,291,905	GCCTGGGTGACTAAGAAAGACTCCATTCCC	CCACGGAGCACCTTGTAGCTCATTAACTGC	720
	**B-B**	**57,518,137-57,291,905**	**GGCACTAATGAAACCAGAAAGACACTTGGC**	**CCACGGAGCACCTTGTAGCTCATTAACTGC**	**839**
	B-C	57,518,137	GGCACTAATGAAACCAGAAAGACACTTGGC	TAGTCATAGTCCCTGATTCCCTTAAAGCGG	831
**UK-SV2**	D-E	57,275,839	CATGACAAAACCCTGTCTCC	CCTATCCAGTAAATGCCTCTTCC	881
	**E-[G]**	**57,456,098-57,559,114**	**ATCAGGCAACACGACACCAT**	**AGAGTGTTAACAAAGTAGACTCGAT**	**1262**
	**[G]-[F]**	57,468,960	GGAGCCTGAAGGAGTTGTCAAA	AATCCAACACATCTTCAGGGCA	999
	**[F]-[E]**	57,456,098	ATCAGGCAACACGACACCAT	TCTTCCACATGGGGACATAGG	894
	**[E]-G**	**57,275,839-57,468,960**	**CCTATCCAGTAAATGCCTCTTCC**	**AATCCAACACATCTTCAGGGCA**	**1427**
	G-H	57,559,114	AGAGTGTTAACAAAGTAGACTCGAT	ACTGGCCAAAGAAAGACCCT	989
**SA-SV3**	I-J	57,247,615	GGGTGCAGTCATTCATTCCT	TCTCTTGAGCCCAGGAAATC	513
	J-K	57,391,678	TCATGTGAAATGCCACCTTC	GAGTGTAACGGCATGGTCTC	1530
	K-L	57,499,214	TTCTTTTAAGGGGGACCTTG	AAGCCAAGATCATCCAAACC	694
	**L-J**	**57,516,678-57,247,615**	**TGCCACTTTCCATATGTGTG**	**TCTCTTGAGCCCAGGAAATC**	**657**
	**J-[M]**	**57,391,678-57,612,711**	**TTATGAATCTGCCCAAGATCAC**	**AATGATTTGCCTTGGCTTTC**	**1022**
	**[M]-L**	**[57,516,678]-57,499,214**	**GAATTTGCTTGAAGGGCTTG**	**AAGCCAAGATCATCCAAACC**	**486**
	L-M	57,516,678	TGCCACTTTCCATATGTGTG	GAATTTGCTTGAAGGGCTTG	702
	M-N	57,612,711	AATGATTTGCCTTGGCTTTC	CAATGCCATACTCTGGACACC	823
**CA-SV4**	O-P	57,233,035	GAAGAGCCAACCAATCACAC	AACAGGCCCAGCTACTCAAG	368
	**P-[R]**	**57,280,008-[57,634,900]**	**ATACAGGGAGACCCCGTTTC**	**CTGATCGAAGTGCAAAATGG**	**1801**
	[R]-[Q]	57,483,883	CTACACAGGGGACTGACACC	CAGCAGCAGCATTATCAACC	677
	**[**Q]-[P]	57,280,008	ATACAGGGAGACCCCGTTTC	AGATGAGTTCTTGCTCTGTTGC	653
	**[P]-R**	**[57,233,035]-57,483,883**	**CAGCAGCAGCATTATCAACC**	**AACAGGCCCAGCTACTCAAG**	**493**
	R-S	57,634,900	CTGATCGAAGTGCAAAATGG	TGGAGGGAAGGTTATCTTGG	2299
**NL-SV5**	T-U	57,260,511	TTCATCATCCACCACCTCCT	TCCATGGACTCCCTGAAACT	893
	**U-U**	**57,515,862-57,260,511**	**TTGCACCGCTGTTAAGAAAG**	**GAAGAGGAGACCCCAAAATG**	**648**
	U-V	57,515,862	CCGCTGTTAAGAAAGGCTCT	CCCACCTCAAGGAGCTTGTA	971
**UK-SV6**	W-X	57,295,969	TAAGGGATTCCAGGAACTTAAATG	AAAATTTGCCAGGGGTGG	767
	**X-X**	**57,510,765-57,295,969**	**TGAGAGAGCTGGAGGCTAGT**	**AAAATTTGCCAGGGGTGG**	**1295**
	X-Y	57,510,765	TGAGAGAGCTGGAGGCTAGT	AGCAACTGCAACTGAACTCCT	1013
**UK-SV7**	Z-AA	57,259,525	TCTTCCGTATCTCTGTCCTCAG	TGGGAGCTCAAGTGGACAAC	1097
	**AA-[AC]**	**57,453,630-57,710,821**	**GCTGGGACTCAGAGGGTGTT**	**AAGCATCTAGGGCACATCCT**	**1554**
	[AC]-[AB]	57,468,931	GGAGCCTGAAGGAGTTGTCAAA	AATCCAACACATCTTCAGGGCA	999
	[AB]-[AA]	57,453,630	GCTGGGACTCAGAGGGTGTT	AACAGTCATGGCTCACACTCA	1200
	**[AA]-AC**	**57,259,525-57,468,931**	**TGGGAGCTCAAGTGGACAAC**	**AATCCAACACATCTTCAGGGCA**	**1504**
	AC-AD	57,710,821	AAGCATCTAGGGCACATCCT	ACCCTATACTGAGGGACCTGC	990

Table continues on next page

SV	Breakpoint	Coordinates	F primer (5'-3')	R primer (5'-3')	Amplicon size (bp)
**UK-SV8**	AE-AF	57,277,347	GCTGACACTTTCCACCCC	CCAATGCAAAACCTGATACAGT	551
	AF-[AH]	**57,326,234-57,631,659**	**TGGGGATGTTCTGCTAAGGG**	**TGCCTGTAGTCCAATTTCTCAG**	458
	[AH]-[AG]	57,413,153	CAGTGGTGTGATCTGCTCA	CACCAAGCATTTTCAGCAGC	478
	[AG]-[AF]	57,326,234	TGGGGATGTTCTGCTAAGGG	TGTGCCCAGCCCTTTTCATT	525
	**[AF]-AH**	**57,277,347-57,413,153**	**CCAATGCAAAACCTGATACAGT**	**CACCAAGCATTTTCAGCAGC**	569
	AH-AI	57,631,659	TGCCTGTAGTCCAATTTCTCAG	GTGTGGGAAGGGTTGCTTAT	433

SV, Structural variant; Breakpoint, Breakpoints between genomic regions as illustrated in Figure 2; Coordinates, genomic positions of breakpoints according to hg19; F primer and R primer, primer sequences used for PCR amplification and Sanger sequencing; Amplicon size, size of amplified PCR product in base pairs (bp). [] indicates inverted segments. Allele-specific mutant breakpoint junctions are indicated in bold.

Table S3: qPCR primers

Target	Primer	Oligonucleotides (5'-3')
SMG8 exons 3-4, mRNA	Forward	ACTAATGCCTCAGGTTCAGC
	Reverse	ATCTCAAAACCCAAAGGCCA
GDPD1 exons 3-5, mRNA	Forward	ATACTGTGAGCTCCCACCTTAC
	Reverse	GGAGTGTTAGGAAAGGCCTCAA
YPEL2 exons 2-4, mRNA	Forward	TCACTGCAGAGCTCACTTGG
	Reverse	CCACAGCCCACATTAACTACTGA
TRIM37 exons 11-12, mRNA	Forward	GCGTCAGAGAGCAGATCC
	Reverse	GCACAACTCCATTTCCATCTG
NRL exons 3-3, mRNA	Forward	GGCTCCACACCTTACAGCTC
	Reverse	AGCCAGTACAGCTCCTCCAG
CRX exons 2-3, mRNA	Forward	GCCCCACTATTCTGTCAACG
	Reverse	CTTCAGAGCCACCTCCTCAC
ACTB exons 3-4, mRNA	Forward	CCAACCGCGAGAAGATGA
	Reverse	CCAGAGGCGTACAGGGATAG
GUSB exons 2-3, mRNA	Forward	AGAGTGGTGCTGAGGATTGG
	Reverse	CCCTCATGCTCTAGCGTGTC
Retinal enhancer, eRNA	Forward	ACCTGCCTGTACGAATCCAA
	Reverse	CTGGGAGGAGGCAAATTGTA
SMG8 exon 4	Forward	CCTGGAAAGAGAAGTGCGGT
Triplication qPCR 1	Reverse	AGGCCCAGAGCACATGAATC
GDPD1 intron 1	Forward	TGTGAATTGAGGGCTCTTCCG
Triplication qPCR 2	Reverse	ACCGTGTCTTTCCCGTTTCA
Downstream of *YPEL2*	Forward	AAGGTCAGCGTTCTCTCAGAAG
Triplication qPCR 3	Reverse	TGTTGAGTTCTGTCTGCCTCG
LINC01476 intron 2	Forward	CCTGCAACCTAACCCTAAGC
Triplication qPCR 4	Reverse	GCATGCCAGATCGCTGTTG

Table S4: Shared heterozygous variants (MAF ≤0.0001, 3 affected individuals) located within the Dutch RP17-locus (NL1)

Chr	Start	End	Ref	Var	gnomAD_G AF	Component	Gene name
chr17	55225642	55225642	G	C	.	intergenic	
chr17	55518925	55518925	C	T	.	intronic	*MSI2*
chr17	55625150	55625150	C	T	.	intronic	*MSI2*
chr17	55645018	55645018	G	A	.	intronic	*MSI2*
chr17	55774511	55774511	A	T	.	intergenic	
chr17	55774518	55774518	C	T	.	intergenic	
chr17	55815842	55815842	C	A	.	intergenic	
chr17	55875136	55875136	C	A	.	ncRNA_intronic	
chr17	56372707	56372707	T	A	.	intergenic	
chr17	56726598	56726598	G	C	.	intronic	*TEX14*
chr17	56769489	56769489	G	C	0.00008155	upstream	*TEX14*
chr17	56811950	56811950	T		.	UTR3	*RAD51C*
chr17	56878006	56878006	C	T	.	intronic	*PPM1E*
chr17	56970337	56970337	G	T	.	intronic	*PPM1E*
chr17	56970342	56970342	A	T	0.00006367	intronic	*PPM1E*
chr17	56970349	56970349	A	C	.	intronic	*PPM1E*
chr17	56970362	56970362	G	A	.	intronic	*PPM1E*
chr17	57232150	57232150	C	G	.	intronic	*SKA2*
chr17	57315768	57315774	TTATTTT		.	intronic	*GDPD1*
chr17	57333198	57333198		TG	.	intronic	*GDPD1*
chr17	57403137	57403137		T	.	intergenic	
chr17	57482817	57482817	A	T	.	upstream	AC091059.1
chr17	57510654	57510654	A	T	.	ncRNA_intronic	
chr17	57717126	57717126	C	T	.	intronic	*CLTC*
chr17	57788781	57788783	ACT		.	intronic	*VMP1*
chr17	57788784	57788784	C	T	.	intronic	*VMP1*
chr17	57812263	57812263		TT	.	intronic	*VMP1*
chr17	57827828	57827828		C	0.00003192	intronic	*VMP1*
chr17	58092315	58092315	T	C	.	ncRNA_intronic	
chr17	58093706	58093706	A	C	.	ncRNA_intronic	
chr17	58203846	58203846	G	A	.	upstream	AC025048.5
chr17	58691382	58691382	T	C	.	intronic	*PPM1D*
chr17	59551786	59551796	CTACCAGCATT		.	intronic	*TBX4*
chr17	59646261	59646261	G	T	.	intergenic	
chr17	59652939	59652939	A	T	.	intergenic	
chr17	59654667	59654667	A	T	.	intergenic	
chr17	59935737	59935737	T	G	.	intronic	*BRIP1*
chr17	59987185	59987192	TGTGTGTG		.	intronic	*INTS2*
chr17	60065110	60065110	A	T	.	intronic	*MED13*
chr17	60223074	60223074	G	C	.	intergenic	
chr17	60223078	60223078	G	T	.	intronic	

Chr; chromosome, Start, End; genomic positions based on hg19, Ref; reference allele, Var; variant, GnomAD_G AF; minor allele frequency according to gnomAD v.2.1.1, Component; genomic position.

Table S5: Shared heterozygous variants (MAF ≤0.0001, 3 affected individuals) located in the founder haplotype in family UK1

Chr	Start	End	Ref	Var	gnomAD_G AF	Component	Gene name
chr17	56059537	56059537	T	C	0.00003228	intronic	*VEZF1*
chr17	56122144	56122144	T	C	0.00003228	intergenic	
chr17	56293716	56293716	G	A	.	intronic	*MKS1*
chr17	56478605	56478605	T	C	.	intronic	*RNF43*
chr17	56731111	56731111	G	A	.	intronic	*TEX14*
chr17	56775478	56775478	T	A	.	intronic	*RAD51C*
chr17	56783547	56783547	T	C	.	intronic	*RAD51C*
chr17	56834462	56834462	C	A	.	intronic	*PPM1E*
chr17	57107553	57107553	G	A	.	intronic	*TRIM37*
chr17	57260755	57260755	A	G	0.0001	intronic	*PRR11*
chr17	57548764	57548764	T	C	.	ncRNA_intronic	*LINC01476*
chr17	57616479	57616479	A	G	0.00009681	intergenic	
chr17	57641653	57641653	G	A	0.000097	intergenic	
chr17	57688592	57688592	T	C	.	intergenic	
chr17	57918969	57918969	G	C	.	UTR3	*VMP1*
chr17	58024808	58024808	A	G	.	UTR3	*RPS6KB1*
chr17	58108605	58108605	G	A	.	intergenic	
chr17	58932373	58932373	C	T	.	intronic	*BCAS3*
chr17	59279276	59279276	C	T	0.00006532	intronic	*BCAS3*
chr17	59328755	59328755	C	A	.	intronic	*BCAS3*
chr17	59913924	59913924	C	T	.	intronic	*BRIP1*
chr17	60391209	60391209	G	A	0.0001	intergenic	
chr17	60404484	60404484	C	G	0.0001	intergenic	
chr17	60428327	60428327	G	T	.	intergenic	
chr17	61687237	61687237	C	T	0.00003228	intergenic	
chr17	61696765	61696765	T	A	0.00006906	intergenic	
chr17	62009718	62009718	C	T	0.00009688	upstream	*CD79B*
chr17	62075612	62075612	A	G	.	ncRNA_intronic	*PRR29-AS1*
chr17	62113494	62113494	C	G	.	intergenic	
chr17	62826064	62826064	A	C	.	ncRNA_intronic	*PLEKHM1P1*
chr17	62834157	62834157	C	G	.	upstream	*PLEKHM1P1*
chr17	62855508	62855508	G	A	0.0001	intronic	*LRRC37A3*

Chr; chromosome, Start, End; genomic positions based on hg19, Ref; reference allele, Var; variant, GnomAD_G AF; minor allele frequency according to gnomAD v.2.1.1, Component; genomic position.

Table S6: Genomic details of RP17-SVs

SV	Type	Chr	Start	End	Event	Size (Mb)	Genomic regions	Genes involved	Detection method
**NL-SV1**	Dup	17	57,291,905	57,518,137	Dup	0.23	B	*SMG8, GDPD1, YPEL2, MIR4729, LINC01476*	FreeC, Manta
**UK-SV2**	DupINVdup	17	57,275,839	57,456,098	Dup	0.18	E	*PRR11, SMG8, GDPD1, YPEL2, MIR4729*	Canvas
			57,275,839	57,559,114	Inv	0.28	E, F, G	*PRR11, SMG8, GDPD1, YPEL2, MIR4729, LINC01476*	Manta
			57,468,960	57,559,114	Dup	0.09	G	*YPEL2, LINC01476*	Canvas
**SA-SV3**	DupINVdup	17	57,247,615	57,391,678	Dup	0.14	J	*PRR11, SMG8, GDPD1*	FreeC
			57,516,678	57,612,711	Inv	0.10	M	*LINC01476*	Manta
			57,499,214	57,612,711	Dup	0.11	L, M	*LINC01476*	FreeC
**CA-SV4**	DupINVdup	17	57,233,035	57,280,008	Dup	0.05	P	*PRR11*	FreeC
			57,233,035	57,634,900	Inv	0.40	P, Q, R	*PRR11, SMG8, GDPD1, YPEL2, MIR4729, LINC01476*	Manta
			57,483,883	57,634,900	Dup	0.15	R	*LINC01476*	FreeC
**NL-SV5**	Dup	17	57,260,511	57,515,862	Dup	0.13	U	*PRR11, SMG8, GDPD1, YPEL2, MIR4729, LINC01476*	FreeC, Manta
**UK-SV6**	Trip	17	57,295,969	57,510,765	Trip	0.21	X	*GDPD1, YPEL2, MIR4729, LINC01476*	Canvas, Manta
**UK-SV7**	DupINVdup	17	57,259,525	57,453,630	Dup	0.16	AA	*PRR11, GDPD1, YPEL2, MIR4729*	Canvas
			57,259,525	57,710,821	Inv	0.42	AA, AB, AC	*PRR11, GDPD1, YPEL2, MIR4729, LINC01476, DHX40, CLTC*	Manta
			57,468,931	57,710,821	Dup	0.24	AC	*YPEL2, LINC01476, DHX40, CLTC*	Canvas
**UK-SV8**	DupINVdup	17	57,277,347	57,326,234	Dup	0.05	AF	*PRR11, SMG8, GDPD1*	Canvas
			57,277,347	57,631,659	Inv	0.35	AF, AG, AH	*PRR11, GDPD1, YPEL2, MIR4729, LINC01476*	Manta
			57,413,153	57,631,659	Dup	0.22	AH	*YPEL2, MIR4729, LINC01476*	Canvas

SV, Structural variant; Type, complex structural rearrangements specified as duplications (dup), inversions (inv), triplications (trip) rearrangements; Start, End, genomic positions of structural rearrangements according to GRCh37/hg19; Size, of structural rearrangement in Mb; Genomic regions annotated as illustrated in Figure 2; Detection method, specific tools employed for identification of structural rearrangements are described in materials and methods section.

Table S7: Repetitive elements identified in sequence flanking the breakpoints

SV	Breakpoint	Coordinates	Repetitive elements
**NL-SV1**	A-B	57,291,905	97.01% SINE/ALU
	B-C	57,518,137	24.92% SINE/MIR
**UK-SV2**	D-E	57,275,839	99.34% SINE/ALU
	E-F	57,456,098	16.28% SINE/ALU
	F-G	57,468,960	44.19% SINE/ALU
	G-H	57,559,114	46.18% SINE/ALU
**SA-SV3**	I-J	57,247,615	54.49% SINE/ALU, 39.53% DNA
	J-K	57,391,678	85.86% SINE/ALU
	K-L	57,499,214	22.92% SINE/ALU
	L-M	57,516,678	55.15% SINE/ALU, 14.29% DNA/hAT-Charlie
	M-N	57,612,711	50.17% SINE/ALU, 20.27% small RNA
**CA-SV4**	O-P	57,233,035	NP
	P-Q	57,280,008	25.58% SINE/ALU
	Q-R	57,483,883	NP
	R-S	57,634,900	96.01% LTR/ERV-class I
**NL-SV5**	T-U	57,260,511	NP
	U-V	57,515,862	50.17% SINE/ALU, 38.21% LINE/L1
**UK-SV6**	W-X	57,295,969	72.76% SINE/ALU
	X-Y	57,510,765	81.06% SINE/ALU
**UK-SV7**	Z-AA	57,259,525	95.68% SINE/ALU
	AA-AB	57,453,630	67.11% SINE/ALU
	AB-AC	57,468,931	33.22% SINE/ALU
	AC-AD	57,710,821	66.45% SINE/ALU
**UK-SV8**	AE-AF	57,277,347	NP
	AF-AG	57,326,234	NP
	AG-AH	57,413,153	68.44% SINE/ALU
	AH-AI	57,631,659	41.86% SINE/ALU, 56.48% LINE/L1

Presence of repetitive elements was assessed using RepeatMasker from the reference sequence, 150 bp reference sequences flanking each side of the breakpoint were used as input. SV, Structural variant; Breakpoint, Breakpoint annotation of genomic regions as illustrated in Figure 2; Coordinates, Genomic position of breakpoint according to GRCh37/hg19 coordinates; Repetitive elements. Percentage of repetitive elements present in input sequence per specified element (class/family); NP, Not present.

Table S8: Assessment of microhomology, insertions and deletions at allele-specific breakpoints

SV	Breakpoint junction	3' Coordinates	5' Coordinates	(Micro)homology	Insertion	Deletion
**NL-SV1**	B-B	57,518,137	57,291,905	5 bp (AGGCA)	-	-
**UK-SV2**	E-[G]	57,456,098	57,559,114	NP	9 bp (TTTTATGAC)	-
	[E]-G	57,275,839	57,468,960	NP	9 bp (AGGCTGGTC)	-
**SA-SV3**	L-J	57,516,678	57,247,615	NP	23 bp (AAAAAAAACTTGAAAAAGAAGTT)	-
	J-[M]	57,391,678	57,612,711	4 bp (TCAG)	-	-
	[M]-L	57,516,678	57,499,214	1 bp (C)	13 bp (GGTCCAGATTGTG)	4 bp (AGAG)
**CA-SV4**	P-[R]	57,280,008	57,634,900	1 bp (T)	-	-
	[P]-R	57,233,035	57,483,883	2 bp (GC)	5 bp (TAAGC)	-
**NL-SV5**	U-U	57,515,862	57,260,511	5 bp (ATCCT)	-	-
**UK-SV6**	X-X	57,510,765	57,295,969	>100 bp	-	-
**UK-SV7**	AA-[AC]	57,453,630	57,710,821	>100 bp	-	-
	[AA]-AC	57,259,525	57,468,931	NP	10 bp (GTAATTTTTC)	-
**UK-SV8**	AF-[AH]	57,326,234	57,631,659	NP	2 bp (CT)	-
	[AF]-AH	57,277,347	57,413,153	2 bp (CT)	-	-

SV, Structural variant; Breakpoint junction, Allele-specific breakpoint junction between genomic regions as illustrated in Figure 2; Coordinates, genomic position of breakpoints according to hg19; Microhomology, presence of microhomology was assessed using ClustalOmega; Insertion and deletion, presence of insertions or deletions as determined by Sanger sequencing. [ ] Indicate inverted segments, bp, base pairs; NP, not present.

Table S9: Clinical findings

SUPPLEMENTAL MATERIALS AND METHODS

SNP genotyping

The RP17-locus was previously established using polymorphic markers selected from the Généthon genetic map, that were genotyped in 23 individuals from index family NL1.[9] Subsequently, we collected DNA from 27 individuals (18 affected and 9 unaffected subjects) from the fourth generation of the family. SNP-genotyping was performed on these 27 DNA samples from generation four, and for 36 individuals (17 affected and 10 unaffected subjects and 9 spouses) from the second and third generation using the HumanCore-24V.1.0 array (Illumina). The RP17-locus was further refined by determining phase in a two-parent-sib dataset.

Exome and Genome sequencing

Index family NL1; Whole exome sequencing (WES) was performed for three affected individuals from different branches of the family. Exome enrichment was performed using the Aligent SureSelect Human All Exome V5 kit following manufacturer's instructions. Subsequently, WES was executed on an Illumina HiSeq2000TM system by BGI Europe (Copenhagen, Denmark). BWA V.0.78[10] and GATK HaplotypeCaller V.3.3[11] were used for read mapping along the hg19 reference genome (GRCh37/hg19) and variant calling, respectively. Variants were annotated using an in-house developed pipeline.

WGS was performed by BGI (Hongkong, China) on a BGISeq500 using a 2x 100 bp paired end module, with a minimal median coverage per genome of 30-fold. SVs were called using Manta Structural Variant Caller V.1.1.0 (Illumina; paired end and split read evidence for SVs) and copy number variants (CNVs) using Control-FREEC (detection of copy number changes and allelic imbalances based on read depth).[12] Variants were validated and visualized using the IGV software (V.2.4).[13] Shared single nucleotide variants (SNVs) or SVs located in or spanning the refined RP17-locus were assessed for putative pathogenicity. Variants were prioritized based on a minor allele frequency (MAF) ≤0.0001 in gnomAD.[14]

Index family UK1; WES was performed for one affected individual as previously described.[15] WGS was subsequently performed for four affected individuals from distant branches of the family by Edinburgh Genomics using TruSeq Nano with a minimal median coverage of 30-fold per genome. Variants were assessed and filtered using the Variant Annotation and Filter Tool (VarAFT).[16] Variants were prioritized based on a MAF ≤0.0001 in gnomAD. CNVs and SVs were analyzed from WES data using ExomeDepth[17] and WGS data using Canvas Copy Number Variant Caller[18] (Illumina; copy number gain or loss based on read depth) and Manta Structural Variant Caller.[19]

For additional unsolved adRP families, or families suspected to harbor RP17-SVs, WES or WGS was performed. Families of Canadian (CA) or South African (SA) origin were analyzed in the Netherlands with additional families of Dutch origin. WGS was performed as described for NL1. For UK families, WGS was executed as described for UK1 or through the NIHR-Bioresource and Genomics England pipelines as previously described.[15; 20]

Characterization and validation of structural variants

Primer sequences and coordinates are listed in Table S2 and PCR conditions for all breakpoint junctions are available upon request.

SV breakpoint regions were assessed for presence of microhomology and repetitive elements. Breakpoint regions and junctions were defined as 150 bp flanking sequence surrounding the breakpoint, which were used as input sequences for subsequent analyses. The presence of microhomology at the breakpoints was assessed using multiple sequence alignment between the junction fragment and the 5' and 3' breakpoint regions using Clustal Omega.[21] The presence of repetitive elements at the breakpoint regions was assessed using RepeatMasker.[22]

To validate the presence of a triplicated region for UK-SV6, a quantitative real-time PCR (qPCR) experiment was performed on genomic DNA from affected individuals from family UK13 (n=2), and unaffected controls (n=2). qPCR was performed using SYBR Green labTAQ Green mix (labTAQ) on a QuantStudio 6 Flex Real-Time PCR System (Applied Biosystems). Primer pairs were designed to amplify genes in the suspected triplicated regions and distal and proximal regions on Chr17 outside the triplicated areas as a reference for standard quantity. Primer sequences and chromosomal positions are listed in Table S3.

Each reaction was run in triplicate and was comprised of 2x labTAQ Green mix (labTAQ), 0.8 µl of each primer (10 mM) and 25 ng DNA in a final reaction volume of 20 µl. Cycling conditions were as follows: 95°C for 2 min, followed by 40 cycles at 95°C for 15 s and 60°C for 20s. Dissociation curves were generated by heat denaturation over a temperature gradient from 60–95°C to ensure no primer-dimers had formed and to check for a single amplicon. To verify the presence of a single PCR product, samples were also electrophoresed on a 2% agarose gel. Data were obtained using the QuantStudio™ Real-Time PCR Software (Applied Biosystems) to generate an amplification plot and a melting curve for each reaction. The fold difference of the target region was normalized to the wild type reference genomic region with respect to the calibrator sample, and was calculated using the ΔΔCt method.[23]

Interrogation of the genomic region

Available Hi-C, ChIP-seq and RNA-seq datasets were downloaded, analyzed and visualized using UCSC genome browser.[24] Human retina ChIP-seq and RNA seq datasets were obtained from Cherry et al. 2020.[25] CTCF ChIP-seq datasets for GM12878 and K562 were retrieved from the ENCODE project/Broad Institute[26] and for MCF-7 from the ENCODE project/University of Washington.[27] CTCF ChiA-PET libraries for K562 and MCF-7 (GSM970215) were obtained from the ENCODE/GIS-Ruan dataset.[28]

Reprogramming fibroblasts into iPSCs and differentiation into photoreceptor progenitor cells and 3D retinal organoids

Fibroblasts were cultured from skin biopsies of individuals with NL-SV1, UK-SV2, and anonymous control individuals. Cell lines were reprogrammed into iPSCs and differentiated in PPCs (NL-SV1) or ROs (UK-SV2).

For NL-SV1, fibroblasts of two affected and four anonymous control individuals were reprogrammed into iPSCs. Reprogramming into iPSCs was performed by lentiviral transduction as previously described[29], for one control cell line, reprogramming was performed using episomal vectors (Addgene).[30] iPSC lines for each affected and control individual were then differentiated into PPCs following the previously described 60-day protocol.[29; 31] For each iPSC line, differentiation was performed for two iPSC clonal lines in triplicate. Differentiation of PPCs was confirmed by RT-qPCR for neural (*PAX6*) and photoreceptor progenitor (*CRX* and *NRL*) markers (data not shown).

For one affected individual with UK-SV2 and one control individual, fibroblasts were reprogrammed into iPSCs using episomal vectors (Addgene), as described previously.[30] Retinal organoids were differentiated from iPSC, following a previously described protocol with slight modifications.[32] iPSCs were seeded on plates coated with Geltrex (ThermoFisher Scientific) until neuronal retinal vesicles (NRVs) appeared. NRVs were excised by a sterile scalpel and distributed in single wells in 25 wells low-attachment plates. NRVs were than cultured in Retinal differentiation media; 3:1 v/v of DMEM:F12, 2% B27 supplement, 1% Non-Essential Amino Acid, 1% Penicillin-Streptomycin (Gibco) for one week. Optic vesicles were then cultured in Retinal Maturation Medium 1 (3:1 v/v of DMEM : F12, 2% B27 supplement, 1% Non-Essential Amino Acid, 1% Penicillin-Streptomycin, 10% Fetal Bovine Serum (Labtech), 100 µM Taurine, 2 mM GlutaMAX) until day 70, then changed to Retinal Maturation Medium 2 (3:1 v/v of DMEM : F12, 1% N2 supplement, 1% Non-Essential Amino Acid, 1% Penicillin-Streptomycin, 10% Fetal Bovine Serum (Labtech), 100 µM Taurine, 2 mM GlutaMAX) until maturation

and collection of the ROs for experimental procedures. Media was supplemented with 1 µM retinoic acid from day 50 to day 70, then changed to 0.5 µM from day 70 to day 100. After day 100 no further supplement was added to the media.

Preparation of low input Hi-C libraries (Low-C)

Four UK-SV2 and four control 200-day old ROs were harvested and dissociated to single cells by gentle trituration in 150 µL PBS. Total volume was brought up to 500 µL with PBS before fixation with 2% PFA/PBS for 10 min while tumbling. Next, 100 µL of 1.425 M glycine were added and incubated in rotation for 5 min. To quench the cross-linking reaction, cells were placed on ice for 10 min. Then, cells were centrifuged for 8 min at 500 g and 4 ˚C, and supernatant was removed. The pellet was resuspended in cold lysis buffer (50 mM Tris pH 7.5, 150 mM NaCl, 5 mM EDTA, 0.5% NP-40, 1.15 Triton X-100, 5% Protease inhibitor cocktail) and incubated for 15 min on ice. Cells were centrifuged for 5 min at 500 g and 4 ˚C, and the supernatant was discarded. Finally, lysed cells were washed in 500 µL PBS and centrifuged for 2 min at 500 g and 4 ˚C. Cells were snap frozen in liquid N2 before restriction enzyme digestion. Next, RO fixed chromatin ($2x10^5$ cells) from UK-SV2 and controls was digested for 2h at 37 ˚C with a 4bp cutter (*DpnII*; New England Biolabs - NEB). The DNA overhangs generated by the restriction enzyme were marked with biotin-14-dATP (Thermo Fischer Scientific) and the proximity ligation step was performed for 4 h at 18 ˚C using T4 DNA ligase (NEB). Crosslink reversal was performed overnight at 65 ˚C with vigorous shaking (1,000 rpm). The DNA was precipitated by adding Phenol-Chloroform-Isoamyl alcohol mix (25:24:1) (Merck) and then sheared to fragments of 300-600 bp using Covaris S220 (2 cycles, each 50sec long; 10% duty; 4 intensity; 200 cycles/burst). The biotin-filled DNA fragments were pulled down using Dynabeads MyOne Streptavidin T1 beads (Thermo Fischer Scientific) and the products were prepared for Illumina short-reads sequencing using the NEBNext Ultra DNA Library Prep kit (NEB).

Quantitative real time PCR of genes and enhancer RNA within the RP17-locus

Expression of genes located in the RP17-locus was assessed using RT-qPCR in human tissues, affected individual and control PPCs and ROs. Commercially available RNA panels were used to determine the expression of *GDPD1* and *YPEL2* in healthy human adult tissues. RNA isolation and cDNA preparation were performed as previously described.[33] Single cell RNA sequencing data of human[2] and primate retinal cell types[3] was obtained and visualized using the Broad Institute Single Cell Portal.

For the PPCs, total RNA was extracted using a Nucleospin RNA kit (Machery-Nagel) and cDNA was synthesized using an iScript cDNA synthesis kit (Bio-Rad). qPCR analysis was performed using GoTaq qPCR Master Mix (Promega) following manufacturer's instructions. 100 day old ROs were harvested and RNA was extracted using RNeasy Mini Kits (Qiagen). cDNA was synthesized using Tetro cDNA Synthesis kits (Bioline) and qPCR analysis was performed using the SYBR Green labTAQ Green mix (labTAQ) following manufacturer's instructions on a QuantStudio 6 Flex Real-Time PCR System (Applied Biosystems).

Primers were designed to assess differential expression of genes implicated in the SVs, and control reference genes and retinal progenitor genes (Table S3). Primers to detect retinal enhancer expression were designed based on observed transcriptional activity of the enhancer RNA in the FANTOM5 Cap Analysis of Gene Expression (CAGE) human dataset (Table S3).[34] Relative gene expression levels, compared to the reference genes *GUSB* and *ACTB*, were determined with the ΔΔCt method.[23] Statistical analyses were performed using an unpaired Student t-test to test for significance between groups.

SUPPLEMENTAL TEXT

RESULTS

Refinement of the RP17-locus in two unrelated adRP families

Index family NL1; In total, 35 affected and 28 unaffected individuals were included. Assuming complete penetrance of the phenotype, a refined locus of 5.16 Mb was identified; chr17:g.55,112,092-60,271,924 (rs8078110-rs9910672) (Figure 1D), with a maximum LOD-score of 15.0. Next, WES was performed in three affected family members from different branches of the family. No rare coding or splice site heterozygous variants (MAF ≤0.0001) located within the defined locus were identified that were shared by all three individuals. In addition, no rare shared heterozygous variants were found in IRD-associated genes (RetNet). Subsequently, WGS was performed in three additional affected individuals. Shared variants within the locus between the three affected individuals were prioritized based on population frequency (MAF ≤0.0001), and coding, splice site, intronic and intergenic heterozygous variants were assessed (Table S4).

Index family UK1; WES was performed for an affected individual from a genetically unexplained UK adRP family (UK1). No rare coding or splice site heterozygous variants (MAF ≤0.0001) were identified in IRD-associated genes, so WGS was performed for four affected individuals (Figure 1B). Prioritization of rare heterozygous variants in genome data shared by affected individuals in this family failed to identify a candidate rare shared heterozygous variant in IRD-associated genes (MAF ≤0.0001); however, a disease associated haplotype on chromosome 17 spanning 8 Mb (17q22-17q24.1) was identified (Figure 1E). No shared rare (MAF ≤0.0001) coding or splice-site variants were identified within the haplotype (Table S5). A deep intronic shared rare (absent from gnomAD) variant (g.56293716G>A; c.262-112C>T; NM_001321269.1), in the ciliopathy gene *MKS1* (MIM: 609883), was initially considered a candidate. This variant was assessed for its potential to alter splicing using lymphoblast RNA extracted from affected individuals and controls; however, no difference in pre-mRNA splicing was observed (data not shown). This rare variant was used as a flag SNV to detect this

haplotype in other families. Twelve UK adRP families were found to carry the same founder haplotype (Figure 1B and Figure 1C). We then refined the adRP locus, by genotyping SNPs in the extended pedigrees, to a 4.4 Mb interval on Chr17q22 (chr17:55,139,138-59,536,883) (Figure 1E).

Identification of structural variants within the RP17-locus

We analyzed the genome and exome data for CNVs and SVs using Manta, Control-FREEC, Canvas and ExomeDepth. In all families, SVs within the RP17-locus were identified. Triplication was suspected from read-depth of SNVs observed in IGV for UK-SV6. To validate the presence of a triplicated region, qPCR was performed on affected and control genomic DNA for genes and genomic regions implicated in this SV, and proximal and distal genomic regions (as additional controls for copy number).

For all families that harbor SVs in the RP17-locus, reanalysis of sequencing data was performed to exclude other potentially pathogenic variants in IRD-associated genes. No pathogenic heterozygous coding or splice site variants were observed in genes that have been associated with IRDs (MAF ≤0.0001). NL2 consists of distantly related affected individuals, who were identified as having a common ancestor following the identification of the NL-SV5. In the middle branch of this pedigree, a plausible candidate variant in *ZNF513* (MIM: 613598) was described previously.[35] This variant was absent in WGS data of the other two affected individuals of this family, and therefore does not segregate with disease and is no longer a candidate variant.

A combination of mutational mechanisms created the RP17-SVs

Different mutational mechanisms have been described for the formation of complex SVs in the genome; including replication-based mechanisms, such as microhomology-mediated break-induced replication.[36; 37] Therefore, we analyzed all breakpoint junction sequences to investigate the potential

mechanism(s) that created RP17-SVs. Analysis of breakpoint sequences in the reference genome using the algorithm RepeatMasker identified an enrichment for long repetitive elements (e.g. *Alu*-elements) in all SVs (Table S6). In addition, breakpoint sequences revealed several DNA signatures that are indicative of distinct underlying mechanisms. For some SVs (e.g. NL-SV1), microhomology (2-5bp) was identified at the breakpoints, whereas longer stretches of homology (>100bp) were identified for breakpoints of UK-SV6 and UK-SV7. In these cases, (micro)homology-mediated repair is the likely mechanism giving rise to the SV. For other breakpoints (e.g. UK-SV1), small insertions and deletions were observed at breakpoint junctions, suggesting non-homologous end joining events (Table S8, Figure S4). In all SVs, there is a high content of repetitive elements, suggesting these play a role in both repair mechanisms.

CONSORTIA

UK Inherited Retinal Dystrophy Consortium

Alison Hardcastle, Michael Cheetham, Michel Michaelides, Andrew Webster, Nikolas Pontikos, Alessia Fiorentino, Gavin Arno, Chris Inglehearn, Carmel Toomes, Manir Ali, Martin McKibbin, Claire Smith, Susan Downes, Jing Yu, Stephanie Halford, Suzanne Broadgate, Graeme Black, Rachel Taylor, Panagiotis Sergouniotis

Genomics England Research Consortium

Ambrose J. C.[1], Arumugan P [1]. Baple E. L.[1], Bleda M.[1], Boardman-Pretty F.[1,2], Boissiere J. M.[1], Boustred C. R.[1], Brittain H. [1], Caulfield M. J.[1,2], Chan G. C.[1], Craig C. E. H.[1], Daugherty L. C.[1], de Burca A.[1], Devereau, A.[1], Elgar G.[1,2], Foulger R. E.[1], Fowler T.[1], Furió-Tarí P.[1], Hackett J. M.[1], Halai D.[1], Hamblin A. [1], Henderson S. [1], Holman J. E.[1], Hubbard T. J. P.[1], Ibanez Garikano K. [1], Jackson R.[1], Jones L. J. [1,2], Kasperaviciute D.[1,2], Kayikci M.[1], Lahnstein L.[1], Lawson K.[1], Leigh S. E. A.[1], Leong I. U. S.[1], Lopez F. J.[1], Maleady-Crowe F.[1], Mason J.[1], McDonagh E. M.[1,2], Moutsianas L.[1,2], Mueller M.[1,2], Murugaesu N.[1], Need A. C.[1,2], Odhams C. A.[1], Patch C.[1,2], Perez-Gil D.[1], Polychronopoulos D.[1], Pullinger J.[1], Rahim T.[1], Rendon A.[1], Riesgo-Ferreiro P.[1], Rogers T.[1], Ryten M.[1], Savage K.[1], Sawant K.[1], Scott R. H.[1], Siddiq A.[1], Sieghart A.[1], Smedley D.[1,2], Smith K. R.[1,2], Sosinsky A.[1,2], Spooner W.[1], Stevens H. E.[1], Stuckey A.[1], Sultana R.[1], Thomas E. R. A.[1,2], Thompson S. R.[1], Tregidgo C.[1], Tucci A.[1,2], Walsh E.[1], Watters, S. A.[1], Welland M. J.[1], Williams E.[1], Witkowska K.[1,2], Wood S. M.[1,2], Zarowiecki M.[1].

(1) Genomics England, London, UK.

(2) William Harvey Research Institute, Queen Mary University of London, London, EC1M 6BQ, UK.

SUPPLEMENTAL REFERENCES

1. Fishilevich, S., Nudel, R., Rappaport, N., Hadar, R., Plaschkes, I., Iny Stein, T., Rosen, N., Kohn, A., Twik, M., Safran, M., et al. (2017). GeneHancer: genome-wide integration of enhancers and target genes in GeneCards. Database (Oxford) 2017, bax028.
2. Lukowski, S.W., Lo, C.Y., Sharov, A.A., Nguyen, Q., Fang, L., Hung, S.S., Zhu, L., Zhang, T., Grünert, U., Nguyen, T., et al. (2019). A single-cell transcriptome atlas of the adult human retina. EMBO J 38, e100811.
3. Peng, Y.-R., Shekhar, K., Yan, W., Herrmann, D., Sappington, A., Bryman, G.S., van Zyl, T., Do, M.T.H., Regev, A., and Sanes, J.R. (2019). Molecular Classification and Comparative Taxonomics of Foveal and Peripheral Cells in Primate Retina. Cell 176, 1222-1237.e1222.
4. Tian, Y., Tang, L., Cui, J., and Zhu, X. (2010). Screening for the carbonic anhydrase IV gene mutations in Chinese retinitis pigmentosa patients. Curr Eye Res 35, 440-444.
5. Rebello, G., Ramesar, R., Vorster, A., Roberts, L., Ehrenreich, L., Oppon, E., Gama, D., Bardien, S., Greenberg, J., Bonapace, G., et al. (2004). Apoptosis-inducing signal sequence mutation in carbonic anhydrase IV identified in patients with the RP17 form of retinitis pigmentosa. Proceedings of the National Academy of Sciences of the United States of America 101, 6617-6622.
6. Yang, Z., Alvarez, B.V., Chakarova, C., Jiang, L., Karan, G., Frederick, J.M., Zhao, Y., Sauve, Y., Li, X., Zrenner, E., et al. (2005). Mutant carbonic anhydrase 4 impairs pH regulation and causes retinal photoreceptor degeneration. Human Molecular Genetics 14, 255 - 265.
7. Alvarez, B.V., Vithana, E.N., Yang, Z., Koh, A.H., Yeung, K., Yong, V., Shandro, H.J., Chen, Y., Kolatkar, P., Palasingam, P., et al. (2007). Identification and Characterization of a Novel Mutation in the Carbonic Anhydrase IV Gene that Causes Retinitis Pigmentosa. Investigative Ophthalmology & Visual Science 48, 3459-3468.
8. de Sousa Dias, M., Hernan, I., Pascual, B., Borràs, E., Mañé, B., Gamundi, M.J., and Carballo, M. (2013). Detection of novel mutations that cause autosomal dominant retinitis pigmentosa in candidate genes by long-range PCR amplification and next-generation sequencing. Mol Vis 19, 654-664.
9. den Hollander, A.I., van der Velde-Visser, S.D., Pinckers, A.J.L.G., Hoyng, C.B., Brunner, H.G., and Cremers, F.P.M. (1999). Refined mapping of the gene for autosomal dominant retinitis pigmentosa (RP17) on chromosome 17q22. Human Genetics 104, 73-76.
10. Li, H., and Durbin, R. (2009). Fast and accurate short read alignment with Burrows-Wheeler transform. Bioinformatics 25, 1754-1760.
11. McKenna, A., Hanna, M., Banks, E., Sivachenko, A., Cibulskis, K., Kernytsky, A., Garimella, K., Altshuler, D., Gabriel, S., Daly, M., et al. (2010). The Genome Analysis Toolkit: a MapReduce framework for analyzing next-generation DNA sequencing data. Genome research 20, 1297-1303.
12. Boeva, V., Popova, T., Bleakley, K., Chiche, P., Cappo, J., Schleiermacher, G., Janoueix-Lerosey, I., Delattre, O., and Barillot, E. (2012). Control-FREEC: a tool for assessing copy number and allelic content using next-generation sequencing data. Bioinformatics 28, 423-425.
13. Robinson, J.T., Thorvaldsdóttir, H., Winckler, W., Guttman, M., Lander, E.S., Getz, G., and Mesirov, J.P. (2011). Integrative genomics viewer. Nat Biotechnol 29, 24-26.
14. Karczewski, K.J., Francioli, L.C., Tiao, G., Cummings, B.B., Alföldi, J., Wang, Q., Collins, R.L., Laricchia, K.M., Ganna, A., Birnbaum, D.P., et al. (2020). The mutational constraint spectrum quantified from variation in 141,456 humans. Nature 581, 434-443.
15. Fiorentino, A., Fujinami, K., Arno, G., Robson, A.G., Pontikos, N., Arasanz Armengol, M., Plagnol, V., Hayashi, T., Iwata, T., Parker, M., et al. (2018). Missense variants in the X-linked gene PRPS1 cause retinal degeneration in females. Human Mutation 39, 80-91.
16. Desvignes, J.-P., Bartoli, M., Delague, V., Krahn, M., Miltgen, M., Béroud, C., and Salgado, D. (2018). VarAFT: a variant annotation and filtration system for human next generation sequencing data. Nucleic Acids Res 46, W545-W553.

17. Plagnol, V., Curtis, J., Epstein, M., Mok, K.Y., Stebbings, E., Grigoriadou, S., Wood, N.W., Hambleton, S., Burns, S.O., Thrasher, A.J., et al. (2012). A robust model for read count data in exome sequencing experiments and implications for copy number variant calling. Bioinformatics 28, 2747-2754.
18. Roller, E., Ivakhno, S., Lee, S., Royce, T., and Tanner, S. (2016). Canvas: versatile and scalable detection of copy number variants. Bioinformatics 32, 2375-2377.
19. Chen, X., Schulz-Trieglaff, O., Shaw, R., Barnes, B., Schlesinger, F., Källberg, M., Cox, A.J., Kruglyak, S., and Saunders, C.T. (2016). Manta: rapid detection of structural variants and indels for germline and cancer sequencing applications. Bioinformatics 32, 1220-1222.
20. Fiorentino, A., Yu, J., Arno, G., Pontikos, N., Halford, S., Broadgate, S., Michaelides, M., Carss, K.J., Raymond, F.L., Cheetham, M.E., et al. (2018). Novel homozygous splicing mutations in ARL2BP cause autosomal recessive retinitis pigmentosa. Mol Vis 24, 603-612.
21. Sievers, F., Wilm, A., Dineen, D., Gibson, T.J., Karplus, K., Li, W., Lopez, R., McWilliam, H., Remmert, M., Söding, J., et al. (2011). Fast, scalable generation of high-quality protein multiple sequence alignments using Clustal Omega. In Mol Syst Biol. p 539.
22. Smit, A., Hubley, R., and Green, P. (2013-2015). RepeatMasker Open-4.0. In. (<http://www.repeatmasker.org>.
23. Pfaffl, M.W. (2001). A new mathematical model for relative quantification in real-time RT-PCR. Nucleic Acids Res 29, e45-e45.
24. Kent, W.J., Sugnet, C.W., Furey, T.S., Roskin, K.M., Pringle, T.H., Zahler, A.M., Haussler, and David. (2002). The Human Genome Browser at UCSC. Genome Research 12, 996-1006.
25. Cherry, T.J., Yang, M.G., Harmin, D.A., Tao, P., Timms, A.E., Bauwens, M., Allikmets, R., Jones, E.M., Chen, R., De Baere, E., et al. (2020). Mapping the cis-regulatory architecture of the human retina reveals noncoding genetic variation in disease. Proceedings of the National Academy of Sciences, 201922501.
26. Ram, O., Goren, A., Amit, I., Shoresh, N., Yosef, N., Ernst, J., Kellis, M., Gymrek, M., Issner, R., Coyne, M., et al. (2011). Combinatorial patterning of chromatin regulators uncovered by genome-wide location analysis in human cells. Cell 147, 1628-1639.
27. Sabo, P.J., Hawrylycz, M., Wallace, J.C., Humbert, R., Yu, M., Shafer, A., Kawamoto, J., Hall, R., Mack, J., Dorschner, M.O., et al. (2004). Discovery of functional noncoding elements by digital analysis of chromatin structure. Proceedings of the National Academy of Sciences of the United States of America 101, 16837-16842.
28. Li, G., Ruan, X., Auerbach, R.K., Sandhu, K.S., Zheng, M., Wang, P., Poh, H.M., Goh, Y., Lim, J., Zhang, J., et al. (2012). Extensive promoter-centered chromatin interactions provide a topological basis for transcription regulation. Cell 148, 84-98.
29. Sangermano, R., Bax, N.M., Bauwens, M., van den Born, L.I., De Baere, E., Garanto, A., Collin, R.W.J., Goercharn-Ramlal, A.S.A., den Engelsman-van Dijk, A.H.A., Rohrschneider, K., et al. (2016). Photoreceptor Progenitor mRNA Analysis Reveals Exon Skipping Resulting from the ABCA4 c.5461-10T→C Mutation in Stargardt Disease. Ophthalmology 123, 1375-1385.
30. Schwarz, N., Carr, A.-J., Lane, A., Moeller, F., Chen, L.L., Aguilà, M., Nommiste, B., Muthiah, M.N., Kanuga, N., Wolfrum, U., et al. (2015). Translational read-through of the RP2 Arg120stop mutation in patient iPSC-derived retinal pigment epithelium cells. Human molecular genetics 24, 972-986.
31. Albert, S., Garanto, A., Sangermano, R., Khan, M., Bax, N.M., Hoyng, C.B., Zernant, J., Lee, W., Allikmets, R., Collin, R.W.J., et al. (2018). Identification and Rescue of Splice Defects Caused by Two Neighboring Deep-Intronic ABCA4 Mutations Underlying Stargardt Disease. Am J Hum Genet 102, 517-527.
32. Gonzalez-Cordero, A., Kruczek, K., Naeem, A., Fernando, M., Kloc, M., Ribeiro, J., Goh, D., Duran, Y., Blackford, S.J.I., Abelleira-Hervas, L., et al. (2017). Recapitulation of Human Retinal Development from Human Pluripotent Stem Cells Generates Transplantable Populations of Cone Photoreceptors. Stem Cell Reports 9, 820-837.

33. de Bruijn, S.E., Verbakel, S.K., de Vrieze, E., Kremer, H., Cremers, F.P.M., Hoyng, C.B., van den Born, L.I., and Roosing, S. (2018). Homozygous variants in KIAA1549, encoding a ciliary protein, are associated with autosomal recessive retinitis pigmentosa. Journal of Medical Genetics 55, 705.
34. Lizio, M., Abugessaisa, I., Noguchi, S., Kondo, A., Hasegawa, A., Hon, C.C., de Hoon, M., Severin, J., Oki, S., Hayashizaki, Y., et al. (2018). Update of the FANTOM web resource: expansion to provide additional transcriptome atlases. Nucleic Acids Res 47, D752-D758.
35. Astuti, G.D.N., van den Born, L.I., Khan, M.I., Hamel, C.P., Bocquet, B., Manes, G., Quinodoz, M., Ali, M., Toomes, C., McKibbin, M., et al. (2018). Identification of Inherited Retinal Disease-Associated Genetic Variants in 11 Candidate Genes. Genes (Basel) 9, 21.
36. Zhang, F., Khajavi, M., Connolly, A.M., Towne, C.F., Batish, S.D., and Lupski, J.R. (2009). The DNA replication FoSTeS/MMBIR mechanism can generate genomic, genic and exonic complex rearrangements in humans. Nature Genetics 41, 849.
37. Sen, S.K., Han, K., Wang, J., Lee, J., Wang, H., Callinan, P.A., Dyer, M., Cordaux, R., Liang, P., and Batzer, M.A. (2006). Human Genomic Deletions Mediated by Recombination between Alu Elements. The American Journal of Human Genetics 79, 41-53.
